# Anchored enrichment dataset for true flies (order Diptera) reveals insights into the phylogeny of flower flies (family Syrphidae)

**DOI:** 10.1186/s12862-016-0714-0

**Published:** 2016-06-29

**Authors:** Andrew Donovan Young, Alan R. Lemmon, Jeffrey H. Skevington, Ximo Mengual, Gunilla Ståhls, Menno Reemer, Kurt Jordaens, Scott Kelso, Emily Moriarty Lemmon, Martin Hauser, Marc De Meyer, Bernhard Misof, Brian M. Wiegmann

**Affiliations:** Canadian National Collection of Insects, Arachnids and Nematodes, Agriculture and Agri-Food Canada, K.W. Neatby Building, 960 Carling Avenue, Ottawa, ON K1A 0C6 Canada; Department of Biology, Carleton University, 1125 Colonel By Drive, Ottawa, ON K1S 5B6 Canada; Department of Scientific Computing, Florida State University, Dirac Science Library, Tallahassee, FL 32306-4102 USA; Zoologisches Forschungsmuseum Alexander Koenig, Leibniz Institute for Animal Biodiversity, Adenauerallee 160, Bonn, D-53113 Germany; Finnish Museum of Natural History, University of Helsinki, Zoology unit, P.O.Box 17, FIN-00014 Helsinki, Finland; Naturalis Biodiversity Center, EIS, P.O. Box 9517, 2300 RA Leiden, The Netherlands; Invertebrates Section, Royal Museum for Central Africa, Leuvensesteenweg 13, 3080 Tervuren, Belgium; Department of Biological Science, Florida State University, 319 Stadium Dr., P.O. Box 3064295, Tallahassee, FL 32306-4295 USA; Plant Pest Diagnostics Branch, California Department of Food & Agriculture, 3294 Meadowview Road, Sacramento, CA 95832-1448 USA; Zoologisches Forschungsmuseum Alexander Koenig, Zentrum für molekulare Biodiversitätsforschung, Adenauerallee 160, Bonn, D-53113 Germany; Department of Entomology, North Carolina State University, Raleigh, NC 27695 USA

**Keywords:** Anchored phylogenetics, Hybrid enrichment, Syrphinae, Microdontinae, Eristalinae, Pipizinae, Flower flies, Hoverflies

## Abstract

**Background:**

Anchored hybrid enrichment is a form of next-generation sequencing that uses oligonucleotide probes to target conserved regions of the genome flanked by less conserved regions in order to acquire data useful for phylogenetic inference from a broad range of taxa. Once a probe kit is developed, anchored hybrid enrichment is superior to traditional PCR-based Sanger sequencing in terms of both the amount of genomic data that can be recovered and effective cost. Due to their incredibly diverse nature, importance as pollinators, and historical instability with regard to subfamilial and tribal classification, Syrphidae (flower flies or hoverflies) are an ideal candidate for anchored hybrid enrichment-based phylogenetics, especially since recent molecular phylogenies of the syrphids using only a few markers have resulted in highly unresolved topologies. Over 6200 syrphids are currently known and uncovering their phylogeny will help us to understand how these species have diversified, providing insight into an array of ecological processes, from the development of adult mimicry, the origin of adult migration, to pollination patterns and the evolution of larval resource utilization.

**Results:**

We present the first use of anchored hybrid enrichment in insect phylogenetics on a dataset containing 30 flower fly species from across all four subfamilies and 11 tribes out of 15. To produce a phylogenetic hypothesis, 559 loci were sampled to produce a final dataset containing 217,702 sites. We recovered a well resolved topology with bootstrap support values that were almost universally >95 %. The subfamily Eristalinae is recovered as paraphyletic, with the strongest support for this hypothesis to date. The ant predators in the Microdontinae are sister to all other syrphids. Syrphinae and Pipizinae are monophyletic and sister to each other. Larval predation on soft-bodied hemipterans evolved only once in this family.

**Conclusions:**

Anchored hybrid enrichment was successful in producing a robustly supported phylogenetic hypothesis for the syrphids. Subfamilial reconstruction is concordant with recent phylogenetic hypotheses, but with much higher support values. With the newly designed probe kit this analysis could be rapidly expanded with further sampling, opening the door to more comprehensive analyses targeting problem areas in syrphid phylogenetics and ecology.

**Electronic supplementary material:**

The online version of this article (doi:10.1186/s12862-016-0714-0) contains supplementary material, which is available to authorized users.

## Background

Thanks in part to modern molecular techniques, the field of biological systematics has made great advances in assembling the Tree of Life. Well-supported phylogenetic hypotheses, based partly or entirely on phylogenomic datasets, now exist for many major animal groups, including (holometabolous) insects [1, 2], birds [3–5], mammals [6], and squamates [7]. Phylogenomic analyses have been made possible by the dramatically decreasing costs of genome/transcriptome sequencing of non-model organisms [8]. However, for many phylogenetic questions, a dense, comprehensive sampling of genomes/transcriptomes is a still prohibitively expensive enterprise. In order to generate these comprehensive phylogenomic data sets, several cost-effective alternatives to whole genome or transcriptome sequencing have been proposed.

One such method is hybrid enrichment [9, 10], which uses oligonucleotide probes or “baits” targeting specific areas of the genome in question. These probes hybridize to genomic fragments containing the loci of interest, allowing them to be amplified and sequenced using high-throughput sequencing. Originally developed for medical research on human diseases [10, 11], hybrid enrichment is a flexible technique for which applications in phylogenomic research are just beginning to be realized [4, 12–14]. Unlike traditional polymerase chain reaction (PCR), hybrid enrichment techniques can be used to isolate and amplify many loci in a single reaction, and thus greatly improve the representation of single species in terms of gene coverage in phylogenomic analyses. Furthermore, once a probe kit is developed the cost of a project increases primarily by the number of taxa added (unlike Sanger sequencing which increases by the number of taxa and loci added) [13].

Two major hybrid enrichment methods are currently used for phylogenetic studies: the ultraconserved element (UCE) approach [12] and anchored hybrid enrichment (AHE) [13]. The UCE approach targets highly conserved noncoding regions of the genome [12] while AHE targets highly conserved regions primarily in the coding portion of the genome; specifically, it targets these regions flanked by less conserved regions in an attempt to acquire more data useful for phylogenetic inference [13]. AHE probe kits are also designed to target a wide range of taxonomic groups: the initial probe kit was designed for use across all vertebrate taxa. This was accomplished by comparing the complete genome of five model organisms [15]. While recent studies have used the UCE approach to study ants [16] and a related exon-capture method to study brittle stars [17], the present study is the first invertebrate project conducted using the AHE technique, utilizing the first iteration of insect-specific probes to construct a phylogenetic hypothesis of the dipteran family Syrphidae.

Syrphidae is a large and relatively well-known family of Diptera with over 6200 described species worldwide [18]. The family has traditionally been divided into three subfamilies: Syrphinae, Microdontinae, and Eristalinae [19]. However, Pipizini, a tribe of historically uncertain placement, has recently been elevated to subfamilial level (i.e. Pipizinae) [20]. In addition, latest phylogenetic studies using molecular sequence data [21] and combined molecular and morphological data [22] recover Eristalinae as paraphyletic. Finally, the Microdontinae have been alternately placed within what would now be considered Eristalinae [23, 24], within Syrphinae [25], or as a separate family [26–28] but are currently considered a subfamily [22, 29–35]. In summary, there is no phylogenetic consensus of subfamilial relationships.

The current tribal division of the family is based mostly on adult morphological characters and larval biology [36]. A total of 15 tribes are recognized: Microdontini and Spheginobacchini, in Microdontinae; Brachyopini, Callicerini, Cerioidini, Eristalini, Merodontini, Milesiini, Rhingiini, Sericomyiini, and Volucellini, in Eristalinae; and Bacchini, Paragini, Syrphini, and Toxomerini, in Syrphinae [20]. The subfamily Pipizinae has no tribal subdivision. The classification into tribes has not been generally accepted, and the relationships among them have never been studied in detail for the entire family [27, 37–39]. Some of the genera have been placed in different tribes and some tribes have even been placed in different subfamilies. For instance, Spheginobacchini has been placed within eristalines, syrphines and microdontines [22, 40, 41] as well as “Pipizini” [20]. Moreover, some tribes are not supported by the last molecular phylogenetic studies, such as Brachyopini, Bacchini or Toxomerini, or their placement within a subfamily is uncertain or unresolved as there is no agreement among different works, e.g. Paragini, Volucellini, Merodontini, and Callicerini [20, 39, 42, 43].

Adults of most species of flower flies are conspicuous flower visitors, where they feed on both pollen and nectar [44]. This behaviour has earned the family the common name “flower flies” (also known as “hoverflies”), and has also generated a large amount of interest in the family as pollinators in both natural ecosystems and agricultural crops [45–50]. The only exception are the microdontines, whose adults are rarely seen on flowers, and in some species they do not feed at all [51]. In contrast to the relatively uniform behaviour of the adults, syrphid larvae display an extraordinary diversity of life histories for a single family, including terrestrial and aquatic predators, inquilines in ant, wasp and bumblebee nests, saprophages, mycophages, root borers, stem miners, leaf miners, and wood borers in decaying logs [40, 52, 53]. Larvae of Microdontinae are inquilines in ants’ nests feeding on eggs, larvae and pupae [54], but also may parasite ant pupae [55]. Immature stages of Eristalinae include saprophages in a wide range of decaying organic media from dung to dead wood, some phytophages in various plants, and some predaceous species, i.e. species of the genus *Volucella* Geoffrey, 1762 are wasp- and bee-brood predators, and larvae of *Nepenthosyrphus* Meijere, 1932 are sit-and-wait aquatic predators in the phytotelmata of pitcher plants in SE Asia [40, 53, 56–59]. Larvae of Pipizinae and Syrphinae share a similar feeding mode, but while known pipizine larvae are predatory mostly on woolly or root aphids with waxy secretions and gall-forming hemipterans, the majority of syrphine larvae prey on a broader range of soft-bodied arthropods such as aphids, coccids and psyllids, but also on Thysanoptera, immature Coleoptera, and Lepidoptera caterpillars [60]. The larvae of some Neotropical syrphines develop as stem borers and leaf miners in plants or as pollen feeders [61–64]. This high diversity of natural histories makes syrphid immatures interesting and economically important as they can be biological control agents of plant pests and invasive weeds, re-cyclers of dead plant and animal matter, and pests of some ornamental plants [40, 53, 65, 66].

Hence, a robust phylogeny of syrphids is crucial to tackle the evolution of mimicry [67], to test the coevolution of microdontines and their ant hosts [54], to infer the evolution of larval life histories and the biology of the common ancestor, and to study the evolution of migratory behaviour.

The aim of the current study was to develop a set of AHE probes for use in Diptera, and to use the newly developed probe set to address the systematic position of the more problematic (e.g. unstable placements, unique morphology) taxa within Syrphidae, especially at the subfamilial and tribal level. Due to their high level of diversity, myriad of larval life histories, historical intractability of a robust subfamilial phylogenetic hypothesis, and economic and ecological significance, Syrphidae are an attractive model organism to test the utility of AHE. The project was accomplished by utilizing AHE to obtain genomic data from 559 nuclear gene regions (374 used in the final analyses). Although the main goal of this study was to elucidate phylogenetic relationships within the family Syrphidae, sequence data from a total of 12 cyclorrhaphan Diptera families were captured, illustrating the flexibility of the technique.

Although the current study includes all major clades of Syrphidae, the phylogeny proposed here will eventually form the basis for a much larger and more thoroughly sampled phylogenetic study (http://www.canacoll.org/Diptera/Staff/Skevington/Syrphidae/Syrphidae_World_Phylogeny.htm). This initiative is being conducted by a large group of entomologists and promises to be the largest phylogenetic collaboration attempted on a single family of insects.

## Methods

### Anchored hybrid enrichment laboratory data collection

Data were collected following the general methods of Lemmon et al. [13] through the Center for Anchored Phylogenomics at Florida State University (www.anchoredphylogeny.com). Briefly, 50ul of each genomic DNA sample, with quantity ranging from 11.5 to 985.3 ng) was sonicated to a fragment size of ~150-350 base pairs (bp) using a Covaris E220 Focused-ultrasonicator with Covaris microTUBES. Subsequently, library preparation and indexing were performed on a Beckman-Coulter Biomek FXp liquid-handling robot following a protocol modified from Meyer and Kirschner [68]. One important modification is a size-selection step after blunt-end repair using SPRIselect beads (Beckman-Coulter Inc.; 0.9× ratio of bead to sample volume). Indexed samples were then pooled at equal quantities (typically 12–16 samples per pool), and enrichments were performed on each multi-sample pool using an Agilent Custom SureSelect kit (Agilent Technologies), designed as specified above. After enrichment, the three enrichment pools were pooled in equal quantities for sequencing in one PE150 Illumina HiSeq2000 lane. Sequencing was performed in the Translational Science Laboratory in the College of Medicine at Florida State University.

## Probe development

We began with nucleotide alignments of 4485 protein coding genes for 13 insect species identified by Niehuis et al. [69]. Each alignment contained up to 11 members of Holometabola from five orders (Diptera, Hymenoptera, Lepidoptera, Strepsiptera, and Coleoptera) and two non-holometabolous insects (used as outgroup) from two orders (Anoplura and Hemiptera). A full list of the species and their higher taxonomy is given in Table 1. We then selected a preliminary set of loci containing > =6 taxa and at least one consecutive 120 bp region with >50 % pairwise sequence identity. Sequences for each species were extracted, and exon boundaries were then identified using published genomes (see Table 1 for details) and custom scripts that identified matches between the transcript sequences (Table 2) and the genomes using 40-mers.

**Table 1:** Voucher specimens used to determine exon boundaries for initial probe site selection

	Order	Family	Genus	Specific Epithet	Number of loci
Outgroup	Hemiptera	Aphididae	*Acyrthosiphon*	*pisum*	865
Holometabola	Diptera	Culicidae	*Aedes*	*aegypti*	874
Holometabola	Hymenoptera	Apidae	*Apis*	*mellifera*	937
Holometabola	Lepidoptera	Bombycidae	*Bombyx*	*mori*	962
Holometabola	Diptera	Culicidae	*Culex*	*quinquefasciatus*	874
Holometabola	Diptera	Drosophilidae	*Drosophila*	*melanogaster*	855
Holometabola	Hymenoptera	Formicidae	*Harpegnathos*	*saltator*	927
Holometabola	Strepsiptera	Mengenillidae	*Mengenilla*	*moldrzyki*	959
Holometabola	Hymenoptera	Pteromalidae	*Nasonia*	*vitripennis*	916
Outgroup	Anoplura	Pediculidae	*Pediculus*	*humanus*	954
Holometabola	Hymenoptera	Formicidae	*Pogonomyrmex*	*barbatus*	937
Holometabola	Coleoptera	Cupedidae	*Priacma*	*serrata*	597
Holometabola	Coleoptera	Tenebrionidae	*Tribolium*	*castaneum*	946

**Table 2:** Diptera genomes and transcriptomes used to develop probe kit

Analysis Name	Genus	Specific Epithet	Type	Source	Accession	
aedAeg	*Aedes*	*aegypti*	Genome	NCBI	AAGE02000001	http://www.ncbi.nlm.nih.gov/genome/44
anoGam	*Anopheles*	*gambiae*	Genome	NCBI	CM000360	http://www.ncbi.nlm.nih.gov/genome/46
culQui	*Culex*	*quinquefasciatus*	Genome	NCBI	AAWU01000001	http://www.ncbi.nlm.nih.gov/genome/393
droMel	*Drosophila*	*melanogaster*	Genome	NCBI	AABU01000001	http://www.ncbi.nlm.nih.gov/genome/47
lutLon	*Lutzomyia*	*longipalpis*	Genome	HGSC	AJWK01000001	ftp://ftp.hgsc.bcm.edu/Llongipalpis/
mayDes	*Mayetiola*	*destructor*	Genome	NCBI	AEGA01000001	http://www.ncbi.nlm.nih.gov/genome/2619
phlPap	*Phlebotomus*	*papatasi*	Genome	WUSTL	AJVK01000001	http://genome.wustl.edu/genomes/view/phlebotomus_papatasi
Anabarhynchus	*Anabarhynchus*	*dentiphallus*	Transcriptome	1kite.org	unpublished	http://1kite.org project ID# INSswpTBHRAAPEI-35
Bibio	*Bibio*	*marci*	Transcriptome	1kite.org	GATJ02	http://www.ncbi.nlm.nih.gov/Traces/wgs/?val=GATJ02
Bombylius	*Bombylius*	*major*	Transcriptome	1kite.org	GATI02	http://www.ncbi.nlm.nih.gov/Traces/wgs//?val=GATI02
Chrysosoma	*Heteropsilopus*	*ingenuus*	Transcriptome	1kite.org	unpublished	http://1kite.org project ID# INSswpTAIRAAPEI-19
Episyrphus	*Episyrphus*	*balteatus*	Transcriptome	1kite.org	unpublished	http://1kite.org project ID# INSnfrTAWRAAPEI-11
Exaireta	*Exaireta*	*spinigera*	Transcriptome	1kite.org	unpublished	http://1kite.org project ID# INSswpTAERAAPEI-15
Lipara	*Lipara*	*lucens*	Transcriptome	1kite.org	GAZD02	http://www.ncbi.nlm.nih.gov/Traces/wgs//?val=GAZD02
Meroplius	*Meroplius*	*fasciculatus*	Transcriptome	1kite.org	unpublished	http://1kite.org project ID# INSytvTAARAAPEI-9
Sicus	*Sicus*	*ferrugineus*	Transcriptome	1kite.org	unpublished	http://1kite.org project ID# INShkeTARRAAPEI-46
Triarthria	*Triarthria*	*setipennis*	Transcriptome	1kite.org	GAVA02	http://www.ncbi.nlm.nih.gov/Traces/wgs//?val=GAVA02
Trichocera	*Trichocera*	*saltator*	Transcriptome	1kite.org	GAXZ02	http://www.ncbi.nlm.nih.gov/Traces/wgs//?val=GAXZ02
Chrysops	*Chrysops*	*vittatus*	Transcriptome	Wiegmann	unpublished	Wiegmann Lab, NCSU; Pers. Comm..
Empis	*Empis*	*snoddyi*	Transcriptome	Wiegmann	unpublished	Wiegmann Lab, NCSU; Pers. Comm..
Muscidae	*Musca*	*domestica*	Transcriptome	Wiegmann	unpublished	Wiegmann Lab, NCSU; Pers. Comm..

Together with the alignments, the exon boundaries were used to identify suitable candidate regions (exons) to target using an Anchored Phylogenomics approach, as described by Lemmon et al. [13]. The following requirements were used to select 962 insect-wide targets: 1) the region was at least 150 bp in length, 2) the region contained no exon boundaries, and 3) the region contained no indels. Details of these targets are given in Additional file 1: Table S1. Concatenated alignments have been uploaded to the NCBI Sequence Read Archive (http://www.ncbi.nlm.nih.gov/sra), with accession numbers (Biosample #) available in Table 3. The lengths of these targets ranged from 150 to 863 bp (mean = 187 bp) whereas the pairwise sequences similarity ranged from 45 to 84 % (mean = 66 %).

**Table 3:** Voucher specimens used in phylogenetic analysis. JSS = Jeff Skevington Specimen. All vouchers deposited in CNC

Family	Subfamily	Tribe	Taxon	Accession Number	Genbank #	Biosample #	Locality
Pipunculidae			*Chalarus spurius*	JSS 22746	KU687412	SAMN03352425	Spain, Extremadura
Pipunculidae			*Pipunculus sp. ON12*	JSS 24663	KR260235	SAMN03352426	Canada, Ontario
Platypezidae			*Platypeza sp.*	JSS 24755	KR260237	SAMN03352427	Canada, Ontario
Sepsidae			*Themira nigricornis*	JSS 26210	KR260243	SAMN03352428	Canada, Ontario
Tachinidae			*Epalpus signifer*	JSS 23233	KR260213	SAMN03352424	Canada, Quebec
Syrphidae	Eristalinae	Brachyopini	*Sphegina rufiventris*	JSS 24645	KR260242	SAMN03352330	Canada, Ontario
Syrphidae	Eristalinae	Callicerini	*Callicera montensis*	JSS 23232	KR260209	SAMN03352268	U.S.A., California
Syrphidae	Eristalinae	Eristalini	*Helophilus fasciatus*	JSS 23235	KR260219	SAMN03352282	Canada, Ontario
Syrphidae	Eristalinae	Merodontini	*Eumerus sp.*	JSS 22745	KR260216	SAMN03352286	Spain, Extremadura
Syrphidae	Eristalinae	Merodontini	*Merodon aberrans*	JSS 23236	KR260228	SAMN03352303	Serbia
Syrphidae	Eristalinae	Milesiini	*Brachypalpus oarus*	JSS 17666	KR260208	SAMN03352284	Canada, Quebec
Syrphidae	Eristalinae	Milesiini	*Xylota bicolor*	JSS 26331	KR260244	SAMN03352423	U.S.A., Mississippi
Syrphidae	Eristalinae	Rhingiini	*Cheilosia soror*	JSS 22751	KR260210	SAMN03352305	Serbia
Syrphidae	Eristalinae	Rhingiini	*Ferdinandea buccata*	JSS 26304	KR260217	SAMN03352384	U.S.A., Tennessee
Syrphidae	Eristalinae	Rhingiini	*Rhingia nasica*	JSS 24659	KR260238	SAMN03352342	Canada, Ontario
Syrphidae	Eristalinae	Volucellini	*Copestylum caudatum*	JSS 17391	KR260212	SAMN03352283	U.S.A., New Mexico
Syrphidae	Eristalinae	Volucellini	*Graptomyza sp.*	JSS 25866	KR260218	SAMN03352378	Malaysia, Sabah
Syrphidae	Microdontinae	Microdontini	*Microdon tristis*	JSS 22763	KR260229	SAMN03352280	Canada, Ontario
Syrphidae	Pipizinae		*Heringia calcarata*	JSS 22754	KR260220	SAMN03352265	Canada, Quebec
Syrphidae	Pipizinae		*Pipiza crassipes*	JSS 22759	KR260233	SAMN03352271	U.S.A., Alaska
Syrphidae	Pipizinae		*Pipiza nigripilosa*	JSS 22762	KR260234	SAMN03352277	U.S.A., North Carolina
Syrphidae	Syrphinae	Bacchini	*Baccha elongata*	JSS 22758	KR260206	SAMN03352270	U.S.A., Alaska
Syrphidae	Syrphinae	Bacchini	*Melanostoma mellinum*	JSS 24699	KR260227	SAMN03352376	Canada, Ontario
Syrphidae	Syrphinae	Bacchini	*Platycheirus sp.*	JSS 24698	KR260236	SAMN03352343	Canada, Ontario
Syrphidae	Syrphinae	Paragini	*Paragus haemorrhous*	JSS 26268	KR260231	SAMN03352381	Republic of Korea
Syrphidae	Syrphinae	Syrphini	*Allograpta obliqua*	JSS 26309	KR260202	SAMN03352377	U.S.A., Mississippi
Syrphidae	Syrphinae	Syrphini	*Betasyrphus serarius*	JSS 25987	KR260207	SAMN03352269	Malaysia, Sabah
Syrphidae	Syrphinae	Syrphini	*Citrogramma circumdatus*	JSS 25726	KR260211	SAMN03352288	Indonesia, West Papua
Syrphidae	Syrphinae	Syrphini	*Epistrophe grossulariae*	JSS 18561	KR260214	SAMN03352306	Canada, Ontario
Syrphidae	Syrphinae	Syrphini	*Episyrphus balteatus*	JSS 26269	KR260215	SAMN03352382	Republic of Korea
Syrphidae	Syrphinae	Syrphini	*Leucozona americanum*	JSS 23231	KR260224	SAMN03352264	Canada, Quebec
Syrphidae	Syrphinae	Syrphini	*Ocyptamus fuscipennis*	JSS 26326	KR260230	SAMN03352421	U.S.A., Mississippi
Syrphidae	Syrphinae	Syrphini	*Parasyrphus annulatus*	JSS 22749	KR260232	SAMN03352289	Serbia
Syrphidae	Syrphinae	Syrphini	*Scaeva dignota*	JSS 19737	KR260239	SAMN03352304	Serbia
Syrphidae	Syrphinae	Syrphini	*Sphaerophoria scripta*	JSS 22750	KR260241	SAMN03352292	Serbia

In order to develop an enrichment kit efficient for Diptera, we developed a reference database based on the *Drosophila melanogaster* sequences contained within the 962 target locus alignments, plus 13 established loci provided by Brian Wiegmann [70]. The database contained spaced k-mers derived from conserved sites within each locus. These were used to scan for homologous loci in seven Diptera genomes and 14 Diptera transcriptomes (see Table 2 for complete list). After the sequence best matching to the references was identified for each species x locus combination, alignments were estimated for each locus using MAFFT (Katoh and Standley, 2013; v7.023b with -genafpair and -maxiterate 1000 flags) [71]. Geneious v5.6.4 (Biomatters, available from http://www.geneious.com) was then used to select well-aligned regions that overlapped with the core insect regions, contained high taxon representation (>10 of 21 lineages), and contained low gaps. The 546 chosen anchor locus alignments contained 121–1497 sites (average of 588 sites) and 48 %-84 % pairwise sequence similarity (average = 69 %). The 13 functional locus alignments contained 185–3035 sites (average of 1758 sites) and 50 %-79 % pairwise sequence similarity (average = 66 %).

Finally, in order to ensure efficient enrichment, we checked for high-copy regions (e.g. microsatellites and transposable elements) in each of the seven genome-derived references as follows. First, a database was constructed for each species using all 15-mers found in the trimmed alignments for that species. We also added to the database all 15-mers that were 1 bp removed from the observed 15-mers. The genome for the species was then exhaustively scanned for the presence of these 15-mers and matches were tallied at the alignment positions at which the 15-mer was found. Alignment regions containing > 100,000 counts in any of the seven species were masked to prevent probe tiling across these regions. Probes of 120 bp were tiled uniformly at 1.72× tiling density (57,681 probes total). Final probe regions and the final probe sequences are available as Additional file 2: Table S2 and Additional file 3: Table S3. Scripts used for locus selection and design and alignments are available upon request from ARL.

In essence, the process for choosing probes for the Diptera kit was fundamentally the same as for choosing probes for the vertebrate kit (V1, Lemmon et al. 2012 [13]). The only difference was that alignments containing only genomes formed the basis of the vertebrate kit, whereas alignments containing both genomes and transcriptomes formed the basis of the Diptera kit.

### Anchored hybrid enrichment bioinformatic data analysis

#### Paired-read merging

Typically, between 50 and 75 % of sequenced library fragments had an insert size between 150 and 300 bp. Since 150 bp paired-end sequencing was performed, this means that the majority of the paired reads overlap and thus should be merged prior to assembly. The overlapping reads were identified and merged following Rokyta [72]. In short, for each degree of overlap for each read we computed the probability of obtaining the observed number of matches by chance, and selected degree of overlap that produced the lowest probability, with a *p*-value less than 10^−10^ required to merge reads. When reads are merged, mismatches are reconciled using base-specific quality scores, which were combined to form the new quality scores for the merged read (see [72] for details). Reads failing to meet the probability criterion were kept separate in the assembly. The merging process produces three files one containing merged reads and two containing the unmerged reads.

#### Assembly

The reads were assembled into contigs using an assembler that makes use of both a divergent reference assembly approach to map reads to the probe regions and a *de-novo* assembly approach to extend the assembly into the flanks. The reference assembler uses a library of spaced 20-mers derived from the conserved sites of the alignments used during probe design. A preliminary match was called if at least 17 of 20 matches exist between a spaced k-mer and the corresponding positions in a read. Reads obtaining a preliminary match were then compared to an appropriate reference sequence used for probe design to determine the maximum number of matches out of 100 consecutive bases (all possible gap-free alignments between the read and the reference were considered). The read was considered mapped to the given locus if at least 55 matches were found. Once a read was mapped, an approximate alignment position was estimated using the position of the spaced 20-mer, and all 60-mers existing in the read were stored in a hash table used by the de-novo assembler. The de-novo assembler identified exact matches between a read and one of the 60-mers found in the hash table. Simultaneously using the two levels of assembly described above, the three read files were traversed repeatedly until an entire pass through the reads produced no additional mapped reads.

A list of all 60-mers found in the mapped reads was compiled, the 60-mers were clustered if found together in at least two reads. The 60-mer clusters were then used to separate the reads into clusters for contig estimation. Relative alignment positions of reads within each cluster were then refined in order to increase the agreement across the reads. Up to one gap was also inserted per read if needed to improve the alignment. Note that given sufficient coverage and an absence of contamination, each single-copy locus should produce a single assembly cluster. Low coverage (leading to a break in the assembly), contamination, and gene duplication, can all lead to an increased number of assembly clusters. A whole genome duplication, for example, would increase the number of clusters to two per locus.

Consensus bases were called from assembly clusters as follows. For each site an unambiguous base was called if the bases present were identical or if the polymorphism of that site could be explained as sequencing error, assuming a binomial probability model with the probability of error equal to 0.1 and alpha equal to 0.05. If the polymorphism could not be explained as sequencing error, the ambiguous base was called that corresponded to the IUPAC code. Called bases were soft-masked (made lowercase) for sites with coverage lower than five. A summary of the assembly results is presented in Additional file 4: Table S4.

#### Contamination filtering

In order to filter out possible low-level contaminants, consensus sequences derived from very low coverage assembly clusters (<10 reads) were removed from further analysis. After filtering, consensus sequences were grouped by locus (across individuals) in order to produce sets of homologs.

#### Orthology

Orthology was determined for each locus as follows. First, a pairwise distance measure was computed for pairs of homologs. To compute the pairwise distance between two sequences, we computed the percent of 20-mers observed in the two sequences that were found in both sequences. Note that the list of 20-mers was constructed from consecutive 20-mers as well as spaced 20-mers (every third base), in order to allow increased levels of sequence divergence. Using the distance matrix, we clustered the sequences using a Neighbor-Joining algorithm as follows: Pairwise distances were ranked from smallest to largest. Starting with the smallest value, pairs of sequences from the set of homologs (representing the next distance in the list) were joined into the same cluster. If one of the two sequences was already in a cluster, the clusters were merged. Clusters containing homologs originating from the same individual were not joined, such that when clustering was complete, each cluster contained at most one homolog per species. Sequence clusters containing fewer than 50 % of the species were removed from downstream processing.

#### **Alignment** (MAFFT)

Sequences in each orthologous set were aligned using MAFFT v7.023b [71], with --genafpair and --maxiterate 1000 flags.

#### Alignment trimming

In order to reduce the error in the data, the alignment for each locus was then trimmed/masked using the following procedure. First, each alignment site was identified as "conserved" if the most common character observed was present in > 40 % of the sequences. This step identified sites for which we were confident were aligned correctly for a sufficient portion of the taxa (typically third codon potions would not be included here). Second, 20 bp regions of each sequence that contained < 10 stable sites were masked. This step identified regions of each sequence that were not well aligned to the majority of the sequences and thus should be masked. Third, sites with fewer than 12 unmasked bases were removed from the alignment. This step identified large regions of the alignments that should be removed entirely from the alignment because they contain large quantities of missing data [73].

### Taxon sampling

Representatives of all four Syrphidae subfamilies and 11 tribes were analysed. We also included taxa of another four dipteran families, i.e. Platypezidae [*Platypeza* sp.], Pipunculidae [*Chalarus spurius* (Fallén, 1816) and *Pipunculus* sp.ON12], Sepsidae [*Themira nigricornis* (Meigen, 1826)], and Tachinidae [*Epalpus signifer* (Walker, 1849)]. A total of 30 flower fly species were sampled (Table 3). Syrphid taxa come from four different Biogeographical Regions, but the majority are Nearctic specimens. Morphological identification of syrphids and pipunculids were provided by A.D.Y and J.H.S., other outgroup taxa were morphologically identified by colleagues at the Canadian National Collection of Insects, Arachnids, and Nematodes (CNC).

### DNA extraction

Genomic DNA extractions were obtained with the QIAGEN DNeasy kit (Qiagen Inc., Santa Clara, CA, USA). Full specimens were extracted overnight at 56 °C, and total DNA was purified the following day following the manufacturer’s protocol. Following extraction, specimens were critical-point dried with the EM CPD300 (Leica Microsystems, Vienna, Austria) and deposited at CNC.

### Vouchers

Specimens for the study were collected by Malaise trap or hand-collecting, preserved in 95-100 % ethanol, and placed in a −80 °C freezer until extraction. The voucher data and unique identifiers for the specimens used for the molecular study are presented in Table 3. Specimens have since been critical point dried, mounted, labeled and deposited in the Canadian National Collection of Insects, Arachnids and Nematodes.

The 5' region of the mitochondrial Cytochrome *c* Oxidase Subunit I (COI) gene was sequenced for each specimen in order to act as a surrogate voucher and allow linkage of the exemplars to a large molecular dataset being assembled. Amplification, purification, sequencing and contig assembly were carried out as described in Gibson et al. [74].

COI sequence alignment was straightforward as no indels (insertions or deletions) were found. The alignment was made by hand using Mesquite v2.74 [75] and translated into amino acids to ensure that there were no stop codons. Sequences were submitted to BOLD and uploaded from there to GenBank (Table 3).

### Phylogeny estimation

A maximum likelihood (ML) tree (with 100 bootstrap replicates) for a single concatenated matrix was estimated using RAxML v7.2.6 [76], with the GTR + G substitution model partitioned by locus under default parameters. *Platypeza* was used to root the tree.

## Results

**Fig. 1:**
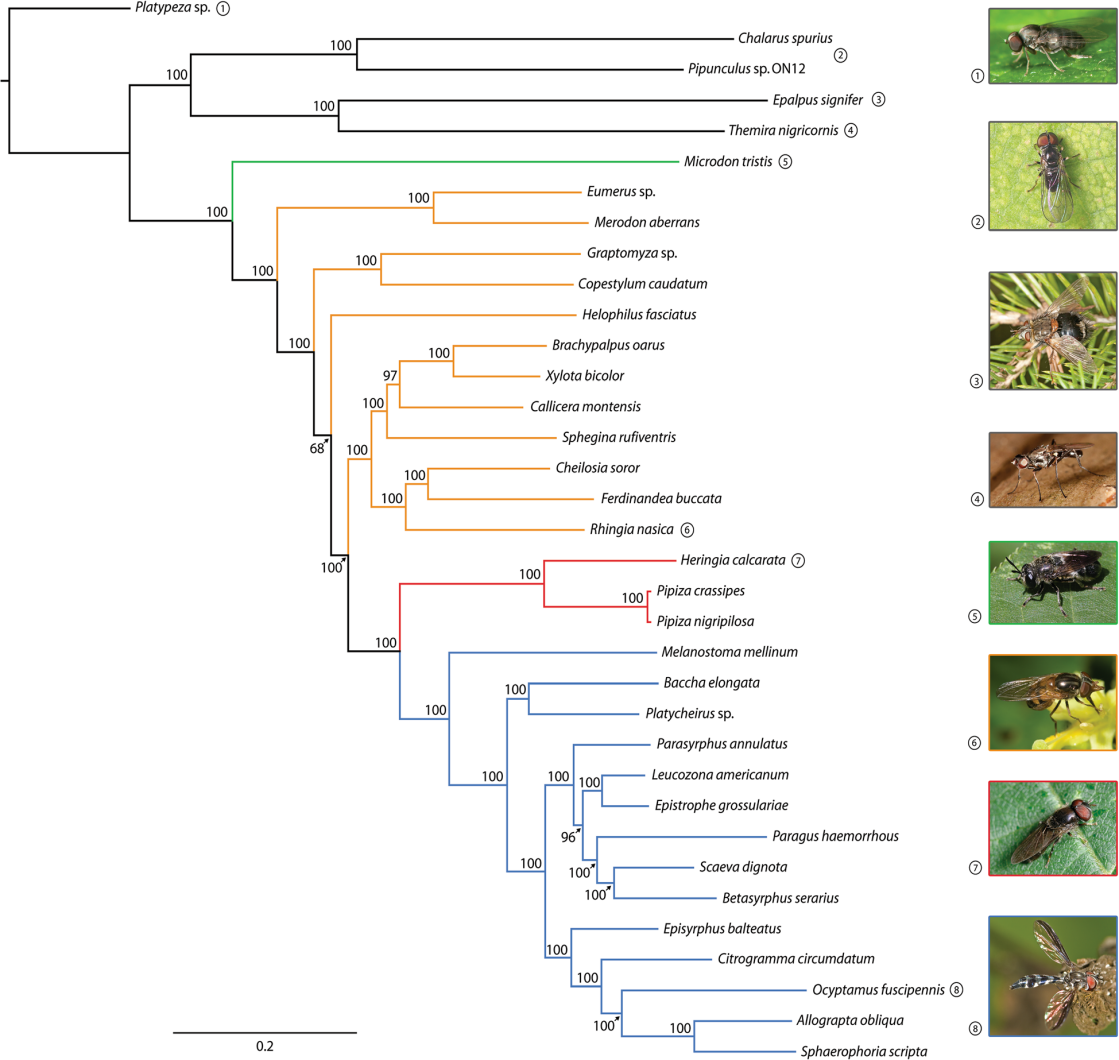
The ML phylogenetic tree based on the sequenced taxa using RAxML under the model GTR + G. Bootstrap support values are depicted above the nodes. Legend: black: outgroups; green: Microdontinae; orange: Eristalinae; red: Pipizinae; and blue: Syrphinae

Trimmed alignments contained 35 taxa and 217,702 sites (across 343 chosen loci), of which 89,534 sites were informative. The concatenated dataset was largely complete, with only 6 % missing data. Maximum Likelihood estimation (Fig. 1) of the present concatenated dataset produced a fully resolved tree, with 31/32 nodes (97 %) supported by >95 % bootstrap support (BS) values. As expected, Syrphidae was recovered as a monophyletic group with *Microdon* Meigen, 1803 as the sister to other lineages (BS = 100 %). The sister clade to the Syrphidae included Pipunculidae + Schizophora. The subfamilies Pipizinae and Syrphinae were resolved as clades. The potential monophyly of the subfamily Microdontinae could not be established (only one taxon included) and Eristalinae was resolved as non-monophyletic. A paraphyletic Eristalinae was placed sister to Syrphinae + Pipizinae. Within the eristalines, several tribes were resolved monophyletic based on the studied taxa. Merodontini (*Eumerus* Meigen, 1822 + *Merodon* Meigen, 1803) was recovered as a clade sister to the remainder of the Eristalinae. Volucellini (*Graptomyza* Weidemann, 1820 + *Copestylum* Macquart, 1846), Rhingiini (*Rhingia* Scopoli, 1763 + *Cheilosia* Meigen, 1822 + *Ferdinandea* Rondani, 1844) and Milesiini (*Brachypalpus* Macquart, 1834 + *Xylota* Meigen, 1822) were also found to be monophyletic. The three remaining tribes that were included in the analysis (Eristalini, Brachyopini, and Callicerini) only had a single member included, so potential monophyly could not be established. Within Syrphinae, three of the four tribes were included, i.e. Syrphini, Bacchini, and Paragini, but not Toxomerini. Bacchini was recovered as paraphyletic, with *Melanostoma* Schiner, 1860 placed as sister to the remainder of the Syrphinae, and a clade consisting of *Baccha* Fabricius, 1805 + *Platycheirus* Lepeletier & Serville, 1828 sister to Syrphinae excluding *Melanostoma*. Syrphini is a large tribe comprised of the majority of the syrphine genera, and formed a single clade with *Paragus* (the sole member of Paragini) resolved within it.

## Discussion

This analysis represents the first iteration of newly developed Diptera probes for AHE. While the probes were developed by analysing the genome of only 21 insect species*,* they were successfully used to extract sequence data from 18 additional Dipteran families (data not shown). Furthermore, while 559 loci were targeted designed, only 343 loci were included in the final analysis in order to minimize missing data. As more invertebrate genomes become available and probe kits are refined, ever larger datasets will be attainable from a broad spectrum of invertebrate taxa for a fraction of the cost of traditional Sanger sequencing methods [13].

The ML analysis produced a fully-resolved phylogram, with only one node with low bootstrap support (BS = 68 %) (see Fig. 1). While previous analyses have recovered similar phylogenies [20–22], no previous works have recovered a fully-resolved tree with high support. A possible explanation for this surprising result is the high number of loci and bp included in our analysis bases on the newly-designed probes, which might allow fully resolved phylogenies for other dipteran families to be produced. The present analysis includes the largest genomic dataset ever created for the phylogenetic analysis of an insect/Diptera family, with 343 loci and 217,702 bp.

The two Pipunculidae taxa were recovered as sister to *Epalpus signifer* (Tachinidae) and *Themira nigricornis* (Sepsidae), both schizophoran flies. Although traditional morphological analyses [77–80] have supported a sister group relationship between Syrphoidea (Pipunculidae + Syrphidae) and Schizophora, more recent morphological [81] and molecular [70] analyses suggest a sister group relationship between Pipunculidae and Schizophora, rendering Syrphoidea paraphyletic.

Placement of Microdontinae has a chequered history as pointed out in the introduction. The “ant flies” are morphologically very distinct from the remaining Syrphidae and all species with known larval histories are associated with ants. Larvae are either predatory or parasitoids in ant nests and have developed elaborate pheromone mimicry to carry out this feat [40, 55, 82, 83]. Strong morphological and ecological specializations within the group have made microdontines very difficult to place into phylogenetic context. Thompson [26] was the first to provide quantitative evidence that they are sister to all other Syrphidae species (based on adult morphology). Despite this, other contradictory hypotheses have continued to be proposed. For example, in their study of larval characters and evolution, Rotheray and Gilbert [84] presented a hypothesis supporting a sister-group relationship between Microdontinae and pipizines and syrphines. This hypothesis assumed a single predatory larval lineage within Syrphidae. Our study refutes this and supports Thompson [26] and several recent molecular studies using Sanger sequence data [20–22, 35]. Proposals as per Thompson [27] and Speight (1987, 2014) [28, 85] have been made to elevate the ant flies to family status and although our present results do not refute this, it remains an argument largely based on the perceived level of morphological and ecological difference of ant flies from other syrphids. Microdontinae is a highly diverse clade and still understudied taxonomically and biologically [35, 41, 54, 55]. Only one species was available for the present study, but the inclusion of members of the Spheginobacchini as well as other taxa not closely related to the genus *Microdon* [41] will allow testing the relationships among the taxa of this subfamily and having a larger support on its placement among flower flies.

Eristalinae was recovered as paraphyletic in the present study. The monophyly of Eristalinae is supported by several studies and the currently followed classification follows this line of reasoning [23, 36, 86, 87]. In contrast, evidence from more recent surveys using adult morphological and/or molecular characters, with a very limited number of loci, resolve Eristalinae as paraphyletic [20–22, 42]. Our analysis is the first to use AHE data from hundreds of loci, and ML analysis of the data provides support for a non-monophyletic Eristalinae (Fig. 1). In the present study, Merodontini was resolved as sister group of the other eristalines + syrphines + pipizines, and Volucellini and *Helophilus* Meigen, 1822 (Eristalini) were recovered in different nodes, with the other included eristaline tribes forming a clade, i.e. Rhingiini, *Sphegina* Meigen, 1822 (Brachyopini), Callicerini and Milesiini. Our taxon sampling is not enough to make conclusions about the tribal relationships within this subfamily. Consequently, a larger and broader taxon sampling is still required, including tribes that were not available for the present study such as Cerioidini and Sericomyiini, to understand how eristaline tribes are related. The only weakly supported node on the maximum likelihood tree is within the Eristalinae. Eristalinae is the subfamily with the highest number of species and larval biology diversity, and it is reflected in the classification with the recognition of nine tribes and several subtribes. Addition of more taxa in future studies will address the question of the monophyly of the subfamily and the tribes, and will also help to better understand larval evolution within this incredibly diverse group of flies.

Syrphinae and Pipizinae were reciprocally monophyletic and sister groups to each other. The placement of Pipizinae as sister to Syrphinae is a phylogenetic hypothesis that has gained increasing support in recent years, and last phylogenetic works have recovered Pipizinae either within Syrphinae [39, 88], or sister to it [20–22, 84]. The frequent placement of Pipizinae within Eristalinae owes much to the fact that many early classification schemes were based largely or entirely on adult morphological characters. The present results strongly suggest a common origin of these two groups, which implies that predatory larvae feeding on soft-bodied arthropods have evolved only once in the evolution of the Syrphidae, and they corroborate previous surveys and the recent elevation of Pipizinae to subfamilial level [20]. Future studies will explore the interrelationships of the members of this subfamily and will test the hypothesis exposed by Vujić et al. [89].

Finally, the resolution of Syrphinae as a monophyletic group was not unexpected as virtually all existing flower fly phylogenies hypothesize that Syrphinae is a clade. In contrast, the current tribal classification within Syrphinae is not supported in our analyses in concordance with the last phylogenetic studies [20, 22, 39, 84, 90]. Bacchini was found to be paraphyletic, and its members (*Melanostoma*, *Platycheirus* and *Baccha*) were resolved in two groups, partly in agreement with previous studies [20, 22, 39]. Paragini, a syrphine tribe of historically uncertain placement, was resolved as sister to *Scaeva* Fabricius, 1805 + *Betasyrphus* Matsumura, 1917, making the current tribe Syrphini paraphyletic. Our results corroborate the hypothesis by Rotheray and Gilbert [38], using larval morphological characters, and by Mengual [91] and Mengual et al. [20], using molecular data alone or in combination with adult morphological characters respectively. Addition of more taxa and the inclusion of the tribe Toxomerini will help to understand the tribal classification of Syrphinae, to define new tribal groups, and, the most important, to study the evolution of predation within this group to answer why and how some taxa became phytophagous secondarily.

The scenario recovered in the present analysis using AHE data shows that predation evolved at least three times in different groups with distinct feeding strategies, viz. Pipizinae + Syrphinae, Microdontinae and Volucellini (although the genus *Volucella* was not studied). A key piece into this puzzle is the unknown biology of the immatures of Spheginobacchini, which would help to understand the relation between microdontines and the rest of flower flies. Excellent mimics of wasps and bumblebees appear in several groups, especially within Eristalinae in genera like *Temnostoma* Lepeletier and Serville, 1828, *Spilomyia* Meigen, 1803 or *Volucella*. The existence of a broad spectrum from non-mimics, through partial or imperfect mimics, to perfect mimics might indicate a multiple origin for mimicry. The same scenario is found when migratory species are taken into consideration based in our results. Species like *Episyrphus balteatus* (De Geer, 1776), *Sphaerophoria scripta* (Linnaeus, 1758) or members of *Scaeva*, *Platycheirus* and *Helophilus* are well-known migrants but little has been studied about the characteristics, origin and mechanisms of these migrations. A fully resolved exhaustively sampled phylogeny based on AHE has the potential to resolve these questions.

## Conclusions

This is the first time that AHE technique is used on an extended and very diverse group of insects and represents the largest dataset assembled to bear on the phylogeny of a dipteran group. The price and repeatability using the present probe kit makes this technique a reliable methodology for future research using large output sequence datasets. Present results corroborate a number of earlier findings and hypotheses, although this dataset should be considered preliminary due to the small taxon sample.

The next step, that is building upon a framework with more thorough taxon sampling of the many morphologically highly diverse groups, will create the most comprehensive hypothesis ever made for a large lineage of flies. With such a high level of ecological and morphological diversity, a detailed phylogeny of Syrphidae will support future work in fields such as pollination biology and biological control, and will help to answer major challenging questions that remain open, such as the evolution of inquiline-host associations in myrmecophilic flies, the evolution of larval feeding behaviour, the development of perfect and imperfect mimicry, the origin and biogeography of the different taxon groups, as well as the patterns of migratory behaviour. As it stands, this study provides a test for previous phylogenetic work on syrphids and illustrates that anchored hybrid enrichment is a useful technique for rapidly assembling comprehensive, large datasets for phylogenetic hypothesis testing. Current anchored data collection and analysis pipelines allow 96 samples to be processed in as little as 3 weeks, from DNA extracts to trimmed alignments and preliminary phylogeny estimates (www.anchoredphylogeny.com).

## Abbreviations

AHE, anchored hybrid enrichment; bp, base pairs; BS, bootstrap support; CNC, Canadian National Collection of Insects, Arachnids, and Nematodes; COI, Cytochrome *c* Oxidase Subunit I; indel, insertion or deletion; ML, maximum likelihood; PCR, polymerase chain reaction; UCE, ultraconserved element.

## Additional files

Additional file 1: Table S1. Preliminary loci for enrichment across Insecta. Complete listing of all insect loci used to determine initial probe site selection. (DOCX 165 kb) Additional file 2: Table S2. Final Probe Regions. Complete listing of all regions used in alignments to generate probes. (XLSX 1446 kb) Additional file 3: Table S3. Final Probe Sequences. Complete listing of all final probes used for Diptera genome amplification. (XLSX 3101 kb) Additional file 4: Table S4. Assembly Results. Complete listing of assemblies produced from the raw reads. (XLSX 18 kb)
