# Supplementary material for: Anchored enrichment dataset for true flies (order Diptera) reveals insights into the phylogeny of flower flies (family Syrphidae)
Source: BMC Evol Biol. 2016 Jun 29;16:143. doi: 10.1186/s12862-016-0714-0 (PMC4928351; doi:10.1186/s12862-016-0714-0)
Supplement: Additional file 1: Table S1. — Preliminary loci for enrichment across Insecta. Complete listing of all insect loci used to determine initial probe site selection. (DOCX 165 kb) [file 12862_2016_714_MOESM1_ESM.docx]

**Additional file 1: Table S1:** Preliminary loci for enrichment across Insecta. Prelim Locus ID = preliminary locus ID, BegPos = beginning position, EndPos = ending position, Target ID = final locus ID, Target Length = targeted locus length, AvgPairSeqSim = average pair sequence similarity, *Tri* = *Tribolium*, *Pri* = *Priacma*, *Api* = *Apis*, *Nas* = *Nasonia*, *Bom* = *Bombyx*, *Dro* = *Drosophila*, *Aed* = *Aedes*, *Cul* = *Culex*, *Har* = *Harpegnathos*, *Pog* = *Pogonomyrmex*, *Men* = *Mengenilla*, *Acy* = *Acyrthosiphon*, *Ped* = *Pediculus*, N Taxa = Number of taxa each locus could be enriched for.

| Prelim Locus ID | BegPos | EndPos | Target ID | Target Length | AvgPairSeqSim | *Tri* | *Pri* | *Api* | *Nas* | *Bom* | *Dro* | *Aed* | *Cul* | *Har* | *Pog* | *Men* | *Acy* | *Ped* | N Taxa |
| --- | --- | --- | --- | --- | --- | --- | --- | --- | --- | --- | --- | --- | --- | --- | --- | --- | --- | --- | --- |
| 3 | 1160 | 1352 | 1 | 193 | 57.88140472 | 1 | 0 | 1 | 1 | 1 | 1 | 0 | 1 | 1 | 0 | 1 | 1 | 1 | 10 |
| 3 | 1549 | 1708 | 2 | 160 | 51.48611111 | 1 | 0 | 1 | 1 | 1 | 1 | 0 | 1 | 1 | 0 | 1 | 1 | 1 | 10 |
| 4 | 492 | 716 | 3 | 225 | 59.73015873 | 0 | 0 | 1 | 0 | 1 | 1 | 1 | 0 | 1 | 1 | 1 | 0 | 1 | 8 |
| 4 | 1451 | 1608 | 4 | 158 | 56.08047016 | 0 | 0 | 1 | 0 | 1 | 1 | 1 | 0 | 1 | 1 | 1 | 0 | 1 | 8 |
| 6 | 2590 | 2749 | 5 | 160 | 68.01388889 | 1 | 0 | 1 | 1 | 1 | 0 | 1 | 1 | 1 | 0 | 1 | 1 | 1 | 10 |
| 8 | 869 | 1041 | 6 | 173 | 74.24242424 | 1 | 1 | 1 | 1 | 1 | 0 | 1 | 1 | 1 | 1 | 1 | 1 | 1 | 12 |
| 9 | 4338 | 4519 | 7 | 182 | 70.63936064 | 1 | 0 | 1 | 1 | 1 | 0 | 1 | 1 | 1 | 1 | 1 | 1 | 1 | 11 |
| 18 | 345 | 505 | 8 | 161 | 57.81918565 | 1 | 0 | 1 | 1 | 1 | 0 | 1 | 0 | 1 | 1 | 1 | 1 | 1 | 10 |
| 18 | 561 | 722 | 9 | 162 | 60.80932785 | 1 | 0 | 1 | 1 | 1 | 0 | 1 | 0 | 1 | 1 | 1 | 1 | 1 | 10 |
| 18 | 724 | 911 | 10 | 188 | 66.47754137 | 1 | 0 | 1 | 1 | 1 | 0 | 1 | 0 | 1 | 1 | 1 | 1 | 1 | 10 |
| 18 | 1957 | 2132 | 11 | 176 | 68.51010101 | 1 | 0 | 1 | 1 | 1 | 0 | 1 | 0 | 1 | 1 | 1 | 1 | 1 | 10 |
| 22 | 360 | 524 | 12 | 165 | 71.71717172 | 1 | 1 | 1 | 1 | 1 | 1 | 1 | 1 | 1 | 1 | 1 | 1 | 1 | 13 |
| 24 | 224 | 513 | 13 | 290 | 73.63113898 | 1 | 1 | 1 | 1 | 1 | 0 | 1 | 1 | 1 | 1 | 1 | 1 | 1 | 12 |
| 24 | 689 | 983 | 14 | 295 | 66.83102209 | 1 | 1 | 1 | 1 | 1 | 0 | 1 | 1 | 1 | 1 | 1 | 1 | 1 | 12 |
| 25 | 1649 | 1804 | 15 | 156 | 76.91336441 | 1 | 1 | 1 | 1 | 1 | 1 | 0 | 1 | 1 | 1 | 1 | 1 | 1 | 12 |
| 27 | 2947 | 3116 | 16 | 170 | 69.7540107 | 1 | 0 | 1 | 1 | 1 | 0 | 1 | 1 | 1 | 1 | 1 | 1 | 1 | 11 |
| 27 | 3150 | 3342 | 17 | 193 | 66.32124352 | 1 | 0 | 1 | 1 | 1 | 0 | 1 | 1 | 1 | 1 | 1 | 1 | 1 | 11 |
| 28 | 511 | 721 | 18 | 211 | 69.97367035 | 1 | 1 | 1 | 1 | 1 | 0 | 0 | 1 | 0 | 1 | 1 | 1 | 1 | 10 |
| 31 | 1661 | 1821 | 19 | 161 | 72.25672878 | 1 | 1 | 1 | 1 | 1 | 0 | 0 | 0 | 1 | 1 | 1 | 1 | 1 | 10 |
| 33 | 3719 | 3902 | 20 | 184 | 62.39130435 | 1 | 0 | 1 | 1 | 1 | 1 | 0 | 0 | 1 | 1 | 1 | 1 | 1 | 10 |
| 35 | 6268 | 6431 | 21 | 164 | 72.22838137 | 1 | 0 | 1 | 1 | 1 | 0 | 1 | 1 | 1 | 1 | 1 | 1 | 1 | 11 |
| 36 | 2836 | 3063 | 22 | 228 | 72.81920078 | 1 | 1 | 1 | 0 | 1 | 0 | 0 | 0 | 1 | 1 | 1 | 1 | 1 | 9 |
| 36 | 3065 | 3230 | 23 | 166 | 74.9665328 | 1 | 1 | 1 | 0 | 1 | 0 | 0 | 0 | 1 | 1 | 1 | 1 | 1 | 9 |
| 39 | 1639 | 1871 | 24 | 233 | 71.25013756 | 1 | 1 | 1 | 1 | 1 | 1 | 1 | 1 | 1 | 1 | 1 | 1 | 1 | 13 |
| 44 | 240 | 428 | 25 | 189 | 70.08177008 | 1 | 0 | 1 | 1 | 1 | 1 | 1 | 1 | 1 | 1 | 1 | 1 | 1 | 12 |
| 47 | 307 | 458 | 26 | 152 | 67.03548644 | 1 | 1 | 1 | 1 | 1 | 1 | 1 | 1 | 0 | 1 | 1 | 1 | 1 | 12 |
| 48 | 5894 | 6068 | 27 | 175 | 64.83116883 | 1 | 0 | 1 | 1 | 1 | 1 | 1 | 1 | 1 | 1 | 1 | 1 | 1 | 12 |
| 49 | 2595 | 2747 | 28 | 153 | 60.34080299 | 1 | 1 | 0 | 0 | 1 | 0 | 1 | 1 | 0 | 0 | 1 | 1 | 1 | 8 |
| 51 | 519 | 726 | 29 | 208 | 64.41579254 | 1 | 0 | 1 | 1 | 1 | 1 | 1 | 1 | 1 | 1 | 1 | 1 | 1 | 12 |
| 57 | 681 | 850 | 30 | 170 | 67.20588235 | 0 | 0 | 1 | 0 | 1 | 1 | 1 | 0 | 1 | 1 | 1 | 1 | 1 | 9 |
| 58 | 0 | 155 | 31 | 156 | 73.3974359 | 1 | 1 | 1 | 1 | 1 | 1 | 1 | 1 | 1 | 1 | 1 | 1 | 1 | 13 |
| 60 | 720 | 869 | 32 | 150 | 69.69047619 | 1 | 0 | 1 | 1 | 1 | 0 | 0 | 0 | 1 | 1 | 1 | 0 | 1 | 8 |
| 60 | 871 | 1036 | 33 | 166 | 65.16781411 | 1 | 0 | 1 | 1 | 1 | 0 | 0 | 0 | 1 | 1 | 1 | 0 | 1 | 8 |
| 61 | 1389 | 1584 | 34 | 196 | 72.06864564 | 1 | 1 | 1 | 0 | 1 | 1 | 1 | 0 | 1 | 1 | 1 | 1 | 1 | 11 |
| 62 | 1087 | 1313 | 35 | 227 | 67.81693588 | 1 | 0 | 0 | 1 | 1 | 0 | 0 | 1 | 1 | 1 | 1 | 1 | 1 | 9 |
| 62 | 1548 | 1721 | 36 | 174 | 66.61877395 | 1 | 0 | 0 | 1 | 1 | 0 | 0 | 1 | 1 | 1 | 1 | 1 | 1 | 9 |
| 64 | 679 | 842 | 37 | 164 | 61.78861789 | 1 | 0 | 1 | 1 | 1 | 1 | 0 | 0 | 1 | 1 | 1 | 1 | 1 | 10 |
| 64 | 1036 | 1213 | 38 | 178 | 69.57553059 | 1 | 0 | 1 | 1 | 1 | 1 | 0 | 0 | 1 | 1 | 1 | 1 | 1 | 10 |
| 66 | 916 | 1079 | 39 | 164 | 59.48817443 | 1 | 0 | 1 | 1 | 1 | 1 | 1 | 1 | 1 | 1 | 1 | 1 | 1 | 12 |
| 66 | 1081 | 1321 | 40 | 241 | 73.20507984 | 1 | 0 | 1 | 1 | 1 | 1 | 1 | 1 | 1 | 1 | 1 | 1 | 1 | 12 |
| 66 | 1524 | 1685 | 41 | 162 | 67.09689487 | 1 | 0 | 1 | 1 | 1 | 1 | 1 | 1 | 1 | 1 | 1 | 1 | 1 | 12 |
| 69 | 412 | 569 | 42 | 158 | 79.97698504 | 1 | 0 | 1 | 1 | 1 | 1 | 1 | 1 | 1 | 1 | 1 | 1 | 1 | 12 |
| 71 | 3980 | 4133 | 43 | 154 | 64.53102453 | 1 | 0 | 1 | 1 | 1 | 0 | 0 | 1 | 1 | 1 | 1 | 1 | 1 | 10 |
| 71 | 4641 | 4819 | 44 | 179 | 66.51769088 | 1 | 0 | 1 | 1 | 1 | 0 | 0 | 1 | 1 | 1 | 1 | 1 | 1 | 10 |
| 73 | 1905 | 2090 | 45 | 186 | 62.37373737 | 1 | 0 | 1 | 1 | 1 | 1 | 1 | 1 | 1 | 1 | 1 | 1 | 1 | 12 |
| 75 | 2436 | 2693 | 46 | 258 | 59.49260042 | 0 | 0 | 1 | 1 | 1 | 1 | 1 | 1 | 1 | 1 | 1 | 1 | 1 | 11 |
| 80 | 2529 | 2745 | 47 | 217 | 73.69920402 | 1 | 1 | 1 | 1 | 1 | 0 | 1 | 1 | 1 | 1 | 1 | 0 | 1 | 11 |
| 80 | 2747 | 2936 | 48 | 190 | 74.04784689 | 1 | 1 | 1 | 1 | 1 | 0 | 1 | 1 | 1 | 1 | 1 | 0 | 1 | 11 |
| 80 | 2940 | 3206 | 49 | 267 | 73.32652366 | 1 | 1 | 1 | 1 | 1 | 0 | 1 | 1 | 1 | 1 | 1 | 0 | 1 | 11 |
| 80 | 3210 | 3380 | 50 | 171 | 70.52631579 | 1 | 1 | 1 | 1 | 1 | 0 | 1 | 1 | 1 | 1 | 1 | 0 | 1 | 11 |
| 82 | 508 | 671 | 51 | 164 | 65.96452328 | 1 | 1 | 1 | 1 | 1 | 1 | 1 | 1 | 0 | 1 | 1 | 1 | 1 | 12 |
| 84 | 231 | 446 | 52 | 216 | 66.40550807 | 1 | 1 | 1 | 1 | 1 | 1 | 1 | 1 | 1 | 1 | 1 | 1 | 1 | 13 |
| 85 | 279 | 452 | 53 | 174 | 68.8958551 | 1 | 0 | 1 | 1 | 1 | 1 | 1 | 1 | 1 | 1 | 1 | 1 | 1 | 12 |
| 86 | 1162 | 1379 | 54 | 218 | 65.57130942 | 1 | 1 | 1 | 1 | 1 | 1 | 0 | 0 | 1 | 1 | 1 | 1 | 1 | 11 |
| 91 | 939 | 1157 | 55 | 219 | 67.36405147 | 1 | 1 | 1 | 1 | 1 | 1 | 0 | 1 | 1 | 0 | 1 | 1 | 1 | 11 |
| 92 | 1356 | 1545 | 56 | 190 | 70.66985646 | 1 | 1 | 1 | 1 | 1 | 0 | 1 | 1 | 1 | 1 | 1 | 1 | 1 | 12 |
| 93 | 5988 | 6158 | 57 | 171 | 66.72869041 | 1 | 0 | 1 | 1 | 1 | 1 | 1 | 1 | 1 | 1 | 1 | 1 | 1 | 12 |
| 94 | 826 | 1004 | 58 | 179 | 62.08566108 | 0 | 0 | 1 | 1 | 1 | 1 | 1 | 1 | 1 | 1 | 1 | 1 | 0 | 10 |
| 94 | 1404 | 1562 | 59 | 159 | 69.825297 | 0 | 0 | 1 | 1 | 1 | 1 | 1 | 1 | 1 | 1 | 1 | 1 | 0 | 10 |
| 94 | 1939 | 2148 | 60 | 210 | 71.33333333 | 0 | 0 | 1 | 1 | 1 | 1 | 1 | 1 | 1 | 1 | 1 | 1 | 0 | 10 |
| 94 | 2698 | 2848 | 61 | 151 | 65.32744665 | 0 | 0 | 1 | 1 | 1 | 1 | 1 | 1 | 1 | 1 | 1 | 1 | 0 | 10 |
| 98 | 2230 | 2448 | 62 | 219 | 76.06837607 | 1 | 1 | 1 | 1 | 1 | 1 | 1 | 1 | 1 | 1 | 1 | 1 | 1 | 13 |
| 98 | 3747 | 3902 | 63 | 156 | 75.3287311 | 1 | 1 | 1 | 1 | 1 | 1 | 1 | 1 | 1 | 1 | 1 | 1 | 1 | 13 |
| 98 | 3904 | 4072 | 64 | 169 | 74.91275982 | 1 | 1 | 1 | 1 | 1 | 1 | 1 | 1 | 1 | 1 | 1 | 1 | 1 | 13 |
| 100 | 696 | 846 | 65 | 151 | 76.98173791 | 1 | 1 | 1 | 1 | 1 | 1 | 1 | 0 | 1 | 1 | 1 | 1 | 1 | 12 |
| 103 | 1818 | 1981 | 66 | 164 | 57.40418118 | 0 | 0 | 0 | 0 | 1 | 1 | 1 | 1 | 0 | 0 | 1 | 1 | 1 | 7 |
| 103 | 2251 | 2433 | 67 | 183 | 67.21311475 | 0 | 0 | 0 | 0 | 1 | 1 | 1 | 1 | 0 | 0 | 1 | 1 | 1 | 7 |
| 103 | 2437 | 2639 | 68 | 203 | 64.46164673 | 0 | 0 | 0 | 0 | 1 | 1 | 1 | 1 | 0 | 0 | 1 | 1 | 1 | 7 |
| 104 | 6672 | 6911 | 69 | 240 | 63.65530303 | 1 | 0 | 1 | 1 | 1 | 1 | 1 | 1 | 1 | 1 | 1 | 1 | 1 | 12 |
| 104 | 8328 | 8479 | 70 | 152 | 64.35406699 | 1 | 0 | 1 | 1 | 1 | 1 | 1 | 1 | 1 | 1 | 1 | 1 | 1 | 12 |
| 105 | 2195 | 2409 | 71 | 215 | 73.32225914 | 1 | 0 | 1 | 1 | 1 | 0 | 0 | 0 | 0 | 1 | 1 | 1 | 1 | 8 |
| 106 | 1589 | 1751 | 72 | 163 | 70.68847989 | 1 | 0 | 1 | 0 | 1 | 1 | 0 | 1 | 1 | 1 | 1 | 0 | 1 | 9 |
| 109 | 2874 | 3041 | 73 | 168 | 61.47186147 | 1 | 1 | 1 | 0 | 1 | 0 | 1 | 1 | 1 | 1 | 1 | 1 | 1 | 11 |
| 110 | 102 | 310 | 74 | 209 | 66.43468175 | 1 | 0 | 1 | 1 | 1 | 1 | 1 | 1 | 1 | 1 | 1 | 1 | 1 | 12 |
| 112 | 2416 | 2566 | 75 | 151 | 65.23178808 | 1 | 0 | 1 | 1 | 1 | 0 | 0 | 1 | 1 | 1 | 1 | 0 | 1 | 9 |
| 114 | 1120 | 1348 | 76 | 229 | 72.57306013 | 1 | 1 | 1 | 1 | 1 | 1 | 1 | 1 | 1 | 1 | 1 | 1 | 1 | 13 |
| 114 | 1456 | 1613 | 77 | 158 | 73.96137618 | 1 | 1 | 1 | 1 | 1 | 1 | 1 | 1 | 1 | 1 | 1 | 1 | 1 | 13 |
| 118 | 437 | 610 | 78 | 174 | 66.98595147 | 0 | 0 | 1 | 1 | 1 | 1 | 1 | 1 | 0 | 0 | 1 | 1 | 1 | 9 |
| 118 | 856 | 1021 | 79 | 166 | 65.26104418 | 0 | 0 | 1 | 1 | 1 | 1 | 1 | 1 | 0 | 0 | 1 | 1 | 1 | 9 |
| 118 | 1023 | 1184 | 80 | 162 | 64.47187929 | 0 | 0 | 1 | 1 | 1 | 1 | 1 | 1 | 0 | 0 | 1 | 1 | 1 | 9 |
| 123 | 2253 | 2453 | 81 | 201 | 78.55168601 | 1 | 0 | 1 | 0 | 1 | 1 | 1 | 1 | 0 | 1 | 1 | 1 | 1 | 10 |
| 123 | 3065 | 3230 | 82 | 166 | 75.90361446 | 1 | 0 | 1 | 0 | 1 | 1 | 1 | 1 | 0 | 1 | 1 | 1 | 1 | 10 |
| 123 | 3436 | 3613 | 83 | 178 | 75.31835206 | 1 | 0 | 1 | 0 | 1 | 1 | 1 | 1 | 0 | 1 | 1 | 1 | 1 | 10 |
| 123 | 3626 | 3784 | 84 | 159 | 76.38015374 | 1 | 0 | 1 | 0 | 1 | 1 | 1 | 1 | 0 | 1 | 1 | 1 | 1 | 10 |
| 124 | 660 | 856 | 85 | 197 | 77.55422243 | 1 | 1 | 1 | 1 | 1 | 0 | 1 | 0 | 1 | 1 | 1 | 1 | 1 | 11 |
| 124 | 1135 | 1305 | 86 | 171 | 73.89686337 | 1 | 1 | 1 | 1 | 1 | 0 | 1 | 0 | 1 | 1 | 1 | 1 | 1 | 11 |
| 127 | 1108 | 1285 | 87 | 178 | 74.76951887 | 1 | 1 | 1 | 1 | 1 | 1 | 1 | 1 | 1 | 1 | 1 | 1 | 1 | 13 |
| 128 | 1626 | 1779 | 88 | 154 | 69.63793782 | 1 | 0 | 1 | 1 | 1 | 1 | 1 | 1 | 1 | 1 | 1 | 1 | 1 | 12 |
| 131 | 39 | 249 | 89 | 211 | 65.58512577 | 1 | 1 | 1 | 1 | 1 | 1 | 1 | 1 | 1 | 1 | 1 | 1 | 1 | 13 |
| 131 | 321 | 713 | 90 | 393 | 65.39766425 | 1 | 1 | 1 | 1 | 1 | 1 | 1 | 1 | 1 | 1 | 1 | 1 | 1 | 13 |
| 132 | 1996 | 2165 | 91 | 170 | 62.69518717 | 1 | 0 | 1 | 1 | 1 | 0 | 1 | 1 | 1 | 1 | 1 | 1 | 1 | 11 |
| 133 | 1039 | 1260 | 92 | 222 | 76.6994267 | 1 | 0 | 1 | 1 | 1 | 1 | 1 | 1 | 1 | 1 | 1 | 0 | 1 | 11 |
| 134 | 1314 | 1545 | 93 | 232 | 67.93364681 | 1 | 0 | 1 | 1 | 1 | 1 | 1 | 1 | 1 | 1 | 1 | 1 | 1 | 12 |
| 134 | 1549 | 1724 | 94 | 176 | 65.60778237 | 1 | 0 | 1 | 1 | 1 | 1 | 1 | 1 | 1 | 1 | 1 | 1 | 1 | 12 |
| 134 | 1783 | 1957 | 95 | 175 | 66.74458874 | 1 | 0 | 1 | 1 | 1 | 1 | 1 | 1 | 1 | 1 | 1 | 1 | 1 | 12 |
| 134 | 2006 | 2212 | 96 | 207 | 71.45366711 | 1 | 0 | 1 | 1 | 1 | 1 | 1 | 1 | 1 | 1 | 1 | 1 | 1 | 12 |
| 134 | 2214 | 2425 | 97 | 212 | 70.31160663 | 1 | 0 | 1 | 1 | 1 | 1 | 1 | 1 | 1 | 1 | 1 | 1 | 1 | 12 |
| 134 | 2637 | 2795 | 98 | 159 | 61.3874595 | 1 | 0 | 1 | 1 | 1 | 1 | 1 | 1 | 1 | 1 | 1 | 1 | 1 | 12 |
| 134 | 3493 | 3696 | 99 | 204 | 68.5828877 | 1 | 0 | 1 | 1 | 1 | 1 | 1 | 1 | 1 | 1 | 1 | 1 | 1 | 12 |
| 134 | 3698 | 3871 | 100 | 174 | 69.58376872 | 1 | 0 | 1 | 1 | 1 | 1 | 1 | 1 | 1 | 1 | 1 | 1 | 1 | 12 |
| 138 | 1011 | 1160 | 101 | 150 | 69.79259259 | 1 | 0 | 0 | 1 | 1 | 0 | 1 | 1 | 1 | 1 | 1 | 1 | 1 | 10 |
| 138 | 1242 | 1416 | 102 | 175 | 73.6 | 1 | 0 | 0 | 1 | 1 | 0 | 1 | 1 | 1 | 1 | 1 | 1 | 1 | 10 |
| 139 | 304 | 517 | 103 | 214 | 70.66259286 | 1 | 1 | 1 | 1 | 1 | 1 | 1 | 1 | 1 | 1 | 1 | 1 | 1 | 13 |
| 140 | 1230 | 1394 | 104 | 165 | 76.83760684 | 1 | 1 | 1 | 1 | 1 | 1 | 1 | 1 | 1 | 1 | 1 | 1 | 1 | 13 |
| 148 | 799 | 951 | 105 | 153 | 68.12240048 | 1 | 1 | 1 | 1 | 1 | 1 | 1 | 1 | 1 | 1 | 1 | 0 | 1 | 12 |
| 148 | 953 | 1121 | 106 | 169 | 70.21696252 | 1 | 1 | 1 | 1 | 1 | 1 | 1 | 1 | 1 | 1 | 1 | 0 | 1 | 12 |
| 149 | 4855 | 5053 | 107 | 199 | 64.32805051 | 1 | 1 | 1 | 1 | 1 | 1 | 1 | 1 | 1 | 1 | 1 | 1 | 1 | 13 |
| 152 | 949 | 1113 | 108 | 165 | 75.27272727 | 1 | 0 | 1 | 1 | 1 | 0 | 1 | 1 | 1 | 1 | 1 | 1 | 1 | 11 |
| 152 | 1139 | 1421 | 109 | 283 | 76.35078702 | 1 | 0 | 1 | 1 | 1 | 0 | 1 | 1 | 1 | 1 | 1 | 1 | 1 | 11 |
| 154 | 3468 | 3658 | 110 | 191 | 65.27843884 | 1 | 1 | 1 | 1 | 1 | 1 | 0 | 1 | 1 | 1 | 1 | 1 | 1 | 12 |
| 160 | 694 | 935 | 111 | 242 | 64.23500742 | 1 | 1 | 1 | 1 | 1 | 1 | 1 | 1 | 1 | 1 | 1 | 1 | 1 | 13 |
| 160 | 1419 | 1658 | 112 | 240 | 61.44230769 | 1 | 1 | 1 | 1 | 1 | 1 | 1 | 1 | 1 | 1 | 1 | 1 | 1 | 13 |
| 161 | 171 | 337 | 113 | 167 | 73.07203774 | 1 | 1 | 1 | 1 | 1 | 1 | 1 | 1 | 1 | 1 | 1 | 0 | 1 | 12 |
| 164 | 1890 | 2045 | 114 | 156 | 53.82834758 | 1 | 0 | 1 | 1 | 1 | 0 | 1 | 1 | 0 | 1 | 1 | 0 | 1 | 9 |
| 165 | 246 | 437 | 115 | 192 | 76.77083333 | 1 | 1 | 1 | 0 | 1 | 1 | 0 | 0 | 1 | 1 | 1 | 1 | 1 | 10 |
| 165 | 439 | 600 | 116 | 162 | 77.11934156 | 1 | 1 | 1 | 0 | 1 | 1 | 0 | 0 | 1 | 1 | 1 | 1 | 1 | 10 |
| 165 | 699 | 863 | 117 | 165 | 75.19191919 | 1 | 1 | 1 | 0 | 1 | 1 | 0 | 0 | 1 | 1 | 1 | 1 | 1 | 10 |
| 167 | 1207 | 1631 | 118 | 425 | 73.3368984 | 1 | 1 | 1 | 1 | 1 | 1 | 1 | 1 | 1 | 1 | 1 | 0 | 1 | 12 |
| 170 | 2144 | 2318 | 119 | 175 | 71.50649351 | 1 | 0 | 1 | 1 | 1 | 1 | 1 | 1 | 1 | 1 | 1 | 1 | 1 | 12 |
| 170 | 2991 | 3149 | 120 | 159 | 77.14884696 | 1 | 0 | 1 | 1 | 1 | 1 | 1 | 1 | 1 | 1 | 1 | 1 | 1 | 12 |
| 170 | 3151 | 3385 | 121 | 235 | 74.12637008 | 1 | 0 | 1 | 1 | 1 | 1 | 1 | 1 | 1 | 1 | 1 | 1 | 1 | 12 |
| 171 | 1365 | 1528 | 122 | 164 | 67.69647696 | 1 | 1 | 1 | 1 | 1 | 0 | 1 | 0 | 1 | 1 | 1 | 0 | 1 | 10 |
| 173 | 204 | 373 | 123 | 170 | 70.15837104 | 1 | 1 | 1 | 1 | 1 | 1 | 1 | 1 | 1 | 1 | 1 | 1 | 1 | 13 |
| 174 | 2493 | 2662 | 124 | 170 | 73.92156863 | 1 | 0 | 0 | 1 | 1 | 0 | 1 | 1 | 1 | 1 | 1 | 1 | 1 | 10 |
| 174 | 3090 | 3244 | 125 | 155 | 65.29032258 | 1 | 0 | 0 | 1 | 1 | 0 | 1 | 1 | 1 | 1 | 1 | 1 | 1 | 10 |
| 175 | 1633 | 1827 | 126 | 195 | 75.4001554 | 1 | 1 | 1 | 1 | 1 | 1 | 1 | 1 | 1 | 1 | 1 | 0 | 1 | 12 |
| 177 | 2719 | 2901 | 127 | 183 | 62.48551085 | 1 | 0 | 1 | 1 | 1 | 1 | 1 | 1 | 1 | 1 | 1 | 1 | 1 | 12 |
| 177 | 3733 | 3900 | 128 | 168 | 66.09848485 | 1 | 0 | 1 | 1 | 1 | 1 | 1 | 1 | 1 | 1 | 1 | 1 | 1 | 12 |
| 177 | 3903 | 4111 | 129 | 209 | 63.16514427 | 1 | 0 | 1 | 1 | 1 | 1 | 1 | 1 | 1 | 1 | 1 | 1 | 1 | 12 |
| 177 | 4330 | 4529 | 130 | 200 | 67.5 | 1 | 0 | 1 | 1 | 1 | 1 | 1 | 1 | 1 | 1 | 1 | 1 | 1 | 12 |
| 177 | 4709 | 4881 | 131 | 173 | 70.46768261 | 1 | 0 | 1 | 1 | 1 | 1 | 1 | 1 | 1 | 1 | 1 | 1 | 1 | 12 |
| 182 | 399 | 599 | 132 | 201 | 66.98326549 | 0 | 0 | 1 | 1 | 1 | 1 | 1 | 1 | 1 | 1 | 1 | 1 | 1 | 11 |
| 182 | 601 | 785 | 133 | 185 | 62.37837838 | 0 | 0 | 1 | 1 | 1 | 1 | 1 | 1 | 1 | 1 | 1 | 1 | 1 | 11 |
| 184 | 2928 | 3129 | 134 | 202 | 66.62916292 | 1 | 0 | 1 | 1 | 1 | 1 | 1 | 1 | 1 | 1 | 1 | 1 | 1 | 12 |
| 184 | 4152 | 4331 | 135 | 180 | 55.55555556 | 1 | 0 | 1 | 1 | 1 | 1 | 1 | 1 | 1 | 1 | 1 | 1 | 1 | 12 |
| 184 | 4333 | 4567 | 136 | 235 | 55.17085751 | 1 | 0 | 1 | 1 | 1 | 1 | 1 | 1 | 1 | 1 | 1 | 1 | 1 | 12 |
| 185 | 679 | 830 | 137 | 152 | 69.46172249 | 1 | 0 | 1 | 1 | 1 | 0 | 1 | 1 | 1 | 1 | 1 | 1 | 1 | 11 |
| 187 | 669 | 829 | 138 | 161 | 66.49944259 | 1 | 1 | 1 | 1 | 1 | 1 | 1 | 1 | 1 | 1 | 1 | 1 | 1 | 13 |
| 190 | 1144 | 1397 | 139 | 254 | 66.48495861 | 1 | 1 | 1 | 1 | 1 | 1 | 1 | 1 | 1 | 1 | 1 | 1 | 1 | 13 |
| 190 | 1422 | 1673 | 140 | 252 | 68.38624339 | 1 | 1 | 1 | 1 | 1 | 1 | 1 | 1 | 1 | 1 | 1 | 1 | 1 | 13 |
| 197 | 127 | 281 | 141 | 155 | 66.02932551 | 1 | 1 | 1 | 1 | 1 | 1 | 0 | 1 | 1 | 1 | 1 | 0 | 1 | 11 |
| 201 | 2343 | 2536 | 142 | 194 | 72.53045923 | 1 | 0 | 1 | 1 | 1 | 0 | 1 | 1 | 1 | 1 | 1 | 1 | 1 | 11 |
| 202 | 679 | 877 | 143 | 199 | 68.28841176 | 1 | 1 | 1 | 1 | 1 | 0 | 1 | 1 | 1 | 1 | 1 | 1 | 1 | 12 |
| 202 | 879 | 1040 | 144 | 162 | 68.91133558 | 1 | 1 | 1 | 1 | 1 | 0 | 1 | 1 | 1 | 1 | 1 | 1 | 1 | 12 |
| 207 | 222 | 390 | 145 | 169 | 76.11895008 | 1 | 1 | 1 | 1 | 1 | 1 | 1 | 1 | 1 | 1 | 1 | 1 | 1 | 13 |
| 211 | 1296 | 1516 | 146 | 221 | 63.57466063 | 1 | 0 | 1 | 1 | 1 | 1 | 1 | 1 | 1 | 1 | 1 | 1 | 1 | 12 |
| 211 | 1526 | 1716 | 147 | 191 | 68.38013644 | 1 | 0 | 1 | 1 | 1 | 1 | 1 | 1 | 1 | 1 | 1 | 1 | 1 | 12 |
| 213 | 4243 | 4449 | 148 | 207 | 72.8151076 | 1 | 0 | 1 | 1 | 1 | 1 | 0 | 1 | 1 | 1 | 1 | 1 | 1 | 11 |
| 216 | 568 | 744 | 149 | 177 | 60.99126862 | 1 | 0 | 1 | 1 | 1 | 1 | 1 | 1 | 1 | 1 | 1 | 1 | 1 | 12 |
| 216 | 4320 | 4478 | 150 | 159 | 60.55841433 | 1 | 0 | 1 | 1 | 1 | 1 | 1 | 1 | 1 | 1 | 1 | 1 | 1 | 12 |
| 218 | 2445 | 2609 | 151 | 165 | 54.15977961 | 1 | 0 | 1 | 1 | 1 | 1 | 1 | 1 | 1 | 1 | 1 | 1 | 1 | 12 |
| 219 | 4163 | 4355 | 152 | 193 | 68.05987334 | 1 | 0 | 0 | 1 | 1 | 0 | 1 | 1 | 1 | 1 | 1 | 1 | 1 | 10 |
| 225 | 1296 | 1449 | 153 | 154 | 73.11097993 | 1 | 1 | 1 | 0 | 1 | 1 | 1 | 1 | 1 | 1 | 1 | 1 | 1 | 12 |
| 225 | 1458 | 1639 | 154 | 182 | 73.31002331 | 1 | 1 | 1 | 0 | 1 | 1 | 1 | 1 | 1 | 1 | 1 | 1 | 1 | 12 |
| 231 | 465 | 617 | 155 | 153 | 61.60625867 | 1 | 1 | 1 | 1 | 1 | 0 | 1 | 1 | 1 | 1 | 1 | 1 | 1 | 12 |
| 231 | 619 | 775 | 156 | 157 | 59.776105 | 1 | 1 | 1 | 1 | 1 | 0 | 1 | 1 | 1 | 1 | 1 | 1 | 1 | 12 |
| 232 | 2139 | 2297 | 157 | 159 | 64.20738591 | 1 | 1 | 1 | 1 | 1 | 1 | 1 | 1 | 1 | 1 | 1 | 1 | 1 | 13 |
| 234 | 8316 | 8475 | 158 | 160 | 77.875 | 1 | 0 | 1 | 1 | 1 | 0 | 1 | 1 | 1 | 1 | 1 | 1 | 1 | 11 |
| 234 | 8622 | 8816 | 159 | 195 | 69.02564103 | 1 | 0 | 1 | 1 | 1 | 0 | 1 | 1 | 1 | 1 | 1 | 1 | 1 | 11 |
| 238 | 7035 | 7236 | 160 | 202 | 62.82628263 | 1 | 0 | 1 | 1 | 1 | 0 | 1 | 1 | 1 | 1 | 1 | 1 | 1 | 11 |
| 238 | 9027 | 9194 | 161 | 168 | 64.27489177 | 1 | 0 | 1 | 1 | 1 | 0 | 1 | 1 | 1 | 1 | 1 | 1 | 1 | 11 |
| 238 | 9490 | 9740 | 162 | 251 | 66.28033321 | 1 | 0 | 1 | 1 | 1 | 0 | 1 | 1 | 1 | 1 | 1 | 1 | 1 | 11 |
| 239 | 1421 | 1688 | 163 | 268 | 70.33921303 | 1 | 1 | 1 | 1 | 1 | 0 | 1 | 1 | 1 | 1 | 1 | 0 | 1 | 11 |
| 243 | 2226 | 2399 | 164 | 174 | 72.0585162 | 1 | 1 | 1 | 1 | 1 | 0 | 1 | 1 | 1 | 1 | 1 | 0 | 1 | 11 |
| 244 | 2940 | 3114 | 165 | 175 | 71.16883117 | 1 | 1 | 1 | 1 | 1 | 0 | 1 | 1 | 1 | 1 | 1 | 0 | 1 | 11 |
| 245 | 619 | 827 | 166 | 209 | 69.35140883 | 1 | 0 | 0 | 1 | 1 | 0 | 1 | 1 | 1 | 1 | 1 | 0 | 1 | 9 |
| 246 | 18 | 221 | 167 | 204 | 69.47586727 | 1 | 1 | 1 | 1 | 1 | 1 | 1 | 1 | 1 | 1 | 1 | 1 | 1 | 13 |
| 250 | 111 | 290 | 168 | 180 | 74.12393162 | 1 | 1 | 1 | 1 | 1 | 1 | 1 | 1 | 1 | 1 | 1 | 1 | 1 | 13 |
| 252 | 180 | 387 | 169 | 208 | 71.66958042 | 1 | 1 | 0 | 1 | 1 | 0 | 1 | 1 | 1 | 1 | 1 | 1 | 1 | 11 |
| 253 | 12 | 188 | 170 | 177 | 73.66362451 | 1 | 1 | 1 | 1 | 1 | 1 | 1 | 1 | 1 | 1 | 1 | 1 | 1 | 13 |
| 256 | 552 | 765 | 171 | 214 | 68.05437553 | 1 | 0 | 1 | 1 | 1 | 1 | 1 | 1 | 1 | 1 | 1 | 1 | 1 | 12 |
| 257 | 1838 | 2077 | 172 | 240 | 73.10897436 | 1 | 1 | 1 | 1 | 1 | 1 | 1 | 1 | 1 | 1 | 1 | 1 | 1 | 13 |
| 260 | 2299 | 2459 | 173 | 161 | 51.74101261 | 1 | 0 | 1 | 1 | 1 | 1 | 1 | 1 | 1 | 1 | 1 | 1 | 1 | 12 |
| 260 | 4611 | 4807 | 174 | 197 | 60.20612214 | 1 | 0 | 1 | 1 | 1 | 1 | 1 | 1 | 1 | 1 | 1 | 1 | 1 | 12 |
| 263 | 0 | 152 | 175 | 153 | 75.73319926 | 1 | 1 | 1 | 1 | 1 | 1 | 1 | 1 | 1 | 1 | 1 | 1 | 1 | 13 |
| 265 | 1044 | 1212 | 176 | 169 | 73.18988704 | 1 | 0 | 1 | 1 | 1 | 1 | 1 | 1 | 1 | 1 | 1 | 0 | 1 | 11 |
| 265 | 1241 | 1395 | 177 | 155 | 68.77419355 | 1 | 0 | 1 | 1 | 1 | 1 | 1 | 1 | 1 | 1 | 1 | 0 | 1 | 11 |
| 265 | 1612 | 1781 | 178 | 170 | 66.32085561 | 1 | 0 | 1 | 1 | 1 | 1 | 1 | 1 | 1 | 1 | 1 | 0 | 1 | 11 |
| 267 | 310 | 464 | 179 | 155 | 78.82697947 | 1 | 0 | 1 | 1 | 1 | 1 | 1 | 1 | 1 | 1 | 1 | 1 | 1 | 12 |
| 267 | 1123 | 1287 | 180 | 165 | 83.95775941 | 1 | 0 | 1 | 1 | 1 | 1 | 1 | 1 | 1 | 1 | 1 | 1 | 1 | 12 |
| 268 | 2643 | 2816 | 181 | 174 | 60.00522466 | 1 | 0 | 1 | 1 | 1 | 1 | 1 | 1 | 1 | 1 | 1 | 1 | 1 | 12 |
| 269 | 215 | 399 | 182 | 185 | 78.89434889 | 1 | 0 | 1 | 1 | 1 | 1 | 1 | 1 | 1 | 1 | 1 | 1 | 1 | 12 |
| 270 | 3523 | 3696 | 183 | 174 | 69.60654288 | 1 | 1 | 1 | 1 | 1 | 1 | 1 | 1 | 1 | 1 | 1 | 1 | 1 | 13 |
| 272 | 867 | 1026 | 184 | 160 | 58.89423077 | 1 | 1 | 1 | 1 | 1 | 1 | 1 | 1 | 1 | 1 | 1 | 1 | 1 | 13 |
| 273 | 1999 | 2194 | 185 | 196 | 65.13605442 | 1 | 1 | 1 | 0 | 1 | 0 | 1 | 1 | 1 | 1 | 1 | 0 | 1 | 10 |
| 275 | 1582 | 1739 | 186 | 158 | 66.75297277 | 1 | 1 | 1 | 1 | 1 | 1 | 1 | 1 | 1 | 1 | 1 | 0 | 1 | 12 |
| 275 | 2169 | 2352 | 187 | 184 | 66.91370224 | 1 | 1 | 1 | 1 | 1 | 1 | 1 | 1 | 1 | 1 | 1 | 0 | 1 | 12 |
| 275 | 2860 | 3058 | 188 | 199 | 63.62874981 | 1 | 1 | 1 | 1 | 1 | 1 | 1 | 1 | 1 | 1 | 1 | 0 | 1 | 12 |
| 276 | 663 | 894 | 189 | 232 | 77.0846395 | 1 | 0 | 1 | 1 | 1 | 1 | 1 | 1 | 1 | 1 | 1 | 0 | 1 | 11 |
| 277 | 2586 | 2744 | 190 | 159 | 52.60148656 | 1 | 0 | 1 | 1 | 1 | 0 | 1 | 1 | 1 | 1 | 1 | 1 | 1 | 11 |
| 280 | 635 | 984 | 191 | 350 | 76.46753247 | 1 | 1 | 1 | 0 | 1 | 1 | 1 | 1 | 1 | 1 | 1 | 0 | 1 | 11 |
| 284 | 1808 | 1985 | 192 | 178 | 75.1174668 | 1 | 0 | 1 | 1 | 1 | 1 | 1 | 0 | 1 | 1 | 1 | 1 | 1 | 11 |
| 289 | 7158 | 7316 | 193 | 159 | 66.39031828 | 1 | 1 | 1 | 1 | 1 | 1 | 1 | 1 | 1 | 0 | 1 | 1 | 1 | 12 |
| 290 | 1259 | 1416 | 194 | 158 | 67.46835443 | 1 | 1 | 1 | 1 | 1 | 1 | 0 | 1 | 1 | 1 | 1 | 0 | 1 | 11 |
| 290 | 1788 | 1967 | 195 | 180 | 66.45454545 | 1 | 1 | 1 | 1 | 1 | 1 | 0 | 1 | 1 | 1 | 1 | 0 | 1 | 11 |
| 290 | 1970 | 2147 | 196 | 178 | 64.09601634 | 1 | 1 | 1 | 1 | 1 | 1 | 0 | 1 | 1 | 1 | 1 | 0 | 1 | 11 |
| 296 | 946 | 1211 | 197 | 266 | 79.40874915 | 1 | 0 | 1 | 1 | 1 | 1 | 1 | 1 | 1 | 1 | 1 | 1 | 1 | 12 |
| 301 | 1147 | 1322 | 198 | 176 | 69.74067599 | 1 | 1 | 1 | 1 | 1 | 1 | 1 | 1 | 1 | 1 | 1 | 1 | 1 | 13 |
| 301 | 1672 | 1841 | 199 | 170 | 61.77224736 | 1 | 1 | 1 | 1 | 1 | 1 | 1 | 1 | 1 | 1 | 1 | 1 | 1 | 13 |
| 306 | 1077 | 1247 | 200 | 171 | 72.59709102 | 1 | 1 | 1 | 1 | 1 | 1 | 1 | 1 | 1 | 1 | 1 | 1 | 1 | 13 |
| 306 | 1650 | 1888 | 201 | 239 | 73.2700354 | 1 | 1 | 1 | 1 | 1 | 1 | 1 | 1 | 1 | 1 | 1 | 1 | 1 | 13 |
| 316 | 870 | 1028 | 202 | 159 | 63.75584583 | 1 | 1 | 1 | 1 | 1 | 1 | 1 | 1 | 1 | 1 | 1 | 1 | 1 | 13 |
| 316 | 1073 | 1256 | 203 | 184 | 60.52118172 | 1 | 1 | 1 | 1 | 1 | 1 | 1 | 1 | 1 | 1 | 1 | 1 | 1 | 13 |
| 317 | 15 | 231 | 204 | 217 | 77.45480326 | 1 | 1 | 1 | 1 | 1 | 1 | 1 | 1 | 1 | 1 | 1 | 1 | 1 | 13 |
| 317 | 435 | 605 | 205 | 171 | 76.4957265 | 1 | 1 | 1 | 1 | 1 | 1 | 1 | 1 | 1 | 1 | 1 | 1 | 1 | 13 |
| 326 | 1120 | 1277 | 206 | 158 | 69.92048036 | 1 | 1 | 1 | 1 | 1 | 1 | 1 | 1 | 1 | 1 | 1 | 1 | 1 | 13 |
| 331 | 4368 | 4523 | 207 | 156 | 60.92074592 | 1 | 0 | 1 | 1 | 1 | 1 | 1 | 0 | 1 | 1 | 1 | 1 | 1 | 11 |
| 332 | 852 | 1052 | 208 | 201 | 63.03331826 | 1 | 0 | 1 | 1 | 1 | 1 | 1 | 1 | 1 | 1 | 1 | 1 | 1 | 12 |
| 338 | 618 | 784 | 209 | 167 | 76.28379604 | 1 | 0 | 1 | 1 | 1 | 1 | 1 | 1 | 1 | 1 | 1 | 1 | 1 | 12 |
| 338 | 814 | 989 | 210 | 176 | 79.0719697 | 1 | 0 | 1 | 1 | 1 | 1 | 1 | 1 | 1 | 1 | 1 | 1 | 1 | 12 |
| 339 | 1052 | 1217 | 211 | 166 | 52.71997079 | 1 | 0 | 1 | 1 | 1 | 1 | 1 | 1 | 1 | 1 | 1 | 1 | 1 | 12 |
| 340 | 1285 | 1452 | 212 | 168 | 71.51515152 | 1 | 0 | 1 | 1 | 1 | 0 | 1 | 1 | 1 | 1 | 1 | 1 | 1 | 11 |
| 341 | 1791 | 1942 | 213 | 152 | 70.24521531 | 1 | 1 | 1 | 1 | 1 | 0 | 1 | 1 | 1 | 1 | 1 | 1 | 1 | 12 |
| 341 | 2235 | 2409 | 214 | 175 | 66.05194805 | 1 | 1 | 1 | 1 | 1 | 0 | 1 | 1 | 1 | 1 | 1 | 1 | 1 | 12 |
| 341 | 2414 | 2566 | 215 | 153 | 70.33075857 | 1 | 1 | 1 | 1 | 1 | 0 | 1 | 1 | 1 | 1 | 1 | 1 | 1 | 12 |
| 341 | 2569 | 2730 | 216 | 162 | 57.36999626 | 1 | 1 | 1 | 1 | 1 | 0 | 1 | 1 | 1 | 1 | 1 | 1 | 1 | 12 |
| 342 | 711 | 862 | 217 | 152 | 68.11740891 | 1 | 1 | 1 | 1 | 1 | 1 | 1 | 1 | 1 | 1 | 1 | 1 | 1 | 13 |
| 346 | 157 | 313 | 218 | 157 | 53.73480023 | 1 | 1 | 1 | 0 | 1 | 1 | 1 | 1 | 1 | 1 | 1 | 1 | 1 | 12 |
| 346 | 564 | 719 | 219 | 156 | 60.53807304 | 1 | 1 | 1 | 0 | 1 | 1 | 1 | 1 | 1 | 1 | 1 | 1 | 1 | 12 |
| 350 | 319 | 506 | 220 | 188 | 77.49032882 | 1 | 1 | 1 | 1 | 1 | 0 | 1 | 1 | 1 | 1 | 1 | 1 | 1 | 12 |
| 350 | 509 | 692 | 221 | 184 | 78.86198946 | 1 | 1 | 1 | 1 | 1 | 0 | 1 | 1 | 1 | 1 | 1 | 1 | 1 | 12 |
| 350 | 990 | 1166 | 222 | 177 | 75.84317754 | 1 | 1 | 1 | 1 | 1 | 0 | 1 | 1 | 1 | 1 | 1 | 1 | 1 | 12 |
| 353 | 1081 | 1268 | 223 | 188 | 72.59026435 | 1 | 0 | 1 | 1 | 1 | 1 | 1 | 1 | 1 | 1 | 1 | 1 | 1 | 12 |
| 353 | 1572 | 1760 | 224 | 189 | 64.75068142 | 1 | 0 | 1 | 1 | 1 | 1 | 1 | 1 | 1 | 1 | 1 | 1 | 1 | 12 |
| 353 | 2766 | 2915 | 225 | 150 | 60.90909091 | 1 | 0 | 1 | 1 | 1 | 1 | 1 | 1 | 1 | 1 | 1 | 1 | 1 | 12 |
| 355 | 1658 | 1807 | 226 | 150 | 63.43589744 | 1 | 1 | 1 | 1 | 1 | 1 | 1 | 1 | 1 | 1 | 1 | 1 | 1 | 13 |
| 359 | 399 | 572 | 227 | 174 | 56.8338558 | 1 | 1 | 1 | 1 | 1 | 1 | 0 | 1 | 1 | 1 | 1 | 0 | 1 | 11 |
| 362 | 852 | 1026 | 228 | 175 | 66.68398268 | 1 | 1 | 1 | 1 | 1 | 1 | 0 | 1 | 1 | 1 | 1 | 1 | 1 | 12 |
| 365 | 564 | 755 | 229 | 192 | 68.27590812 | 1 | 1 | 1 | 1 | 1 | 1 | 1 | 1 | 1 | 1 | 1 | 1 | 1 | 13 |
| 365 | 772 | 921 | 230 | 150 | 61.55555556 | 1 | 1 | 1 | 1 | 1 | 1 | 1 | 1 | 1 | 1 | 1 | 1 | 1 | 13 |
| 368 | 1230 | 1553 | 231 | 324 | 58.7962963 | 1 | 0 | 1 | 1 | 1 | 1 | 1 | 1 | 1 | 1 | 1 | 1 | 1 | 12 |
| 368 | 2520 | 3382 | 232 | 863 | 79.85357632 | 1 | 0 | 1 | 1 | 1 | 1 | 1 | 1 | 1 | 1 | 1 | 1 | 1 | 12 |
| 369 | 3184 | 3362 | 233 | 179 | 61.66412731 | 1 | 1 | 1 | 1 | 1 | 1 | 0 | 1 | 1 | 1 | 1 | 1 | 1 | 12 |
| 369 | 3364 | 3586 | 234 | 223 | 66.80255469 | 1 | 1 | 1 | 1 | 1 | 1 | 0 | 1 | 1 | 1 | 1 | 1 | 1 | 12 |
| 369 | 3590 | 3788 | 235 | 199 | 59.98172682 | 1 | 1 | 1 | 1 | 1 | 1 | 0 | 1 | 1 | 1 | 1 | 1 | 1 | 12 |
| 373 | 1050 | 1211 | 236 | 162 | 60.33554922 | 1 | 1 | 1 | 1 | 1 | 1 | 1 | 1 | 1 | 1 | 1 | 1 | 1 | 13 |
| 373 | 2409 | 2561 | 237 | 153 | 61.92391487 | 1 | 1 | 1 | 1 | 1 | 1 | 1 | 1 | 1 | 1 | 1 | 1 | 1 | 13 |
| 374 | 431 | 608 | 238 | 178 | 66.31767109 | 1 | 0 | 1 | 1 | 1 | 1 | 1 | 1 | 1 | 1 | 1 | 1 | 1 | 12 |
| 375 | 5884 | 6042 | 239 | 159 | 64.47493806 | 1 | 0 | 1 | 1 | 1 | 1 | 1 | 1 | 1 | 1 | 1 | 1 | 1 | 12 |
| 375 | 11945 | 12113 | 240 | 169 | 68.59422629 | 1 | 0 | 1 | 1 | 1 | 1 | 1 | 1 | 1 | 1 | 1 | 1 | 1 | 12 |
| 380 | 2121 | 2279 | 241 | 159 | 51.88679245 | 1 | 0 | 1 | 1 | 1 | 1 | 1 | 1 | 1 | 1 | 1 | 1 | 1 | 12 |
| 383 | 1992 | 2165 | 242 | 174 | 60.1271334 | 1 | 0 | 1 | 1 | 1 | 1 | 1 | 1 | 1 | 1 | 1 | 1 | 1 | 12 |
| 385 | 444 | 673 | 243 | 230 | 68.64295125 | 1 | 0 | 1 | 1 | 1 | 1 | 1 | 1 | 1 | 1 | 1 | 1 | 1 | 12 |
| 386 | 2076 | 2246 | 244 | 171 | 61.208577 | 1 | 1 | 1 | 1 | 1 | 1 | 1 | 1 | 1 | 1 | 1 | 1 | 1 | 13 |
| 387 | 264 | 803 | 245 | 540 | 61.1965812 | 1 | 1 | 1 | 1 | 1 | 1 | 1 | 1 | 1 | 1 | 1 | 1 | 1 | 13 |
| 387 | 911 | 1119 | 246 | 209 | 66.42743222 | 1 | 1 | 1 | 1 | 1 | 1 | 1 | 1 | 1 | 1 | 1 | 1 | 1 | 13 |
| 391 | 630 | 866 | 247 | 237 | 64.02683112 | 1 | 1 | 1 | 1 | 1 | 1 | 1 | 1 | 1 | 1 | 1 | 1 | 1 | 13 |
| 394 | 88 | 251 | 248 | 164 | 73.83592018 | 1 | 0 | 1 | 1 | 1 | 1 | 1 | 1 | 1 | 1 | 1 | 1 | 1 | 12 |
| 396 | 1359 | 1535 | 249 | 177 | 64.63131972 | 1 | 1 | 1 | 1 | 1 | 1 | 1 | 1 | 1 | 1 | 1 | 1 | 1 | 13 |
| 401 | 1752 | 1982 | 250 | 231 | 71.86934278 | 1 | 0 | 1 | 1 | 1 | 0 | 1 | 1 | 1 | 1 | 1 | 1 | 1 | 11 |
| 405 | 1275 | 1448 | 251 | 174 | 68.3908046 | 1 | 1 | 1 | 1 | 1 | 1 | 1 | 1 | 1 | 1 | 1 | 1 | 1 | 13 |
| 418 | 1564 | 1731 | 252 | 168 | 61.3546176 | 1 | 0 | 1 | 1 | 1 | 1 | 1 | 1 | 1 | 1 | 1 | 1 | 1 | 12 |
| 418 | 1746 | 1916 | 253 | 171 | 61.79337232 | 1 | 0 | 1 | 1 | 1 | 1 | 1 | 1 | 1 | 1 | 1 | 1 | 1 | 12 |
| 419 | 70 | 230 | 254 | 161 | 63.17523057 | 1 | 0 | 1 | 1 | 1 | 1 | 1 | 1 | 1 | 1 | 1 | 1 | 1 | 12 |
| 419 | 490 | 656 | 255 | 167 | 63.66358193 | 1 | 0 | 1 | 1 | 1 | 1 | 1 | 1 | 1 | 1 | 1 | 1 | 1 | 12 |
| 420 | 3 | 152 | 256 | 150 | 67.51282051 | 1 | 1 | 1 | 1 | 1 | 1 | 1 | 1 | 1 | 1 | 1 | 1 | 1 | 13 |
| 423 | 57 | 263 | 257 | 207 | 72.45303274 | 1 | 0 | 1 | 1 | 1 | 1 | 0 | 1 | 0 | 1 | 1 | 1 | 1 | 10 |
| 425 | 4506 | 4688 | 258 | 183 | 66.13677761 | 1 | 0 | 1 | 1 | 1 | 1 | 1 | 1 | 1 | 1 | 1 | 1 | 1 | 12 |
| 426 | 9828 | 10024 | 259 | 197 | 65.00538379 | 1 | 0 | 1 | 1 | 1 | 1 | 1 | 1 | 1 | 1 | 1 | 1 | 1 | 12 |
| 428 | 3250 | 3461 | 260 | 212 | 68.18181818 | 1 | 1 | 1 | 1 | 1 | 0 | 0 | 1 | 1 | 1 | 1 | 1 | 1 | 11 |
| 428 | 3598 | 3771 | 261 | 174 | 71.74503657 | 1 | 1 | 1 | 1 | 1 | 0 | 0 | 1 | 1 | 1 | 1 | 1 | 1 | 11 |
| 430 | 1251 | 1403 | 262 | 153 | 47.14795009 | 1 | 0 | 1 | 1 | 1 | 1 | 1 | 1 | 1 | 1 | 1 | 1 | 1 | 12 |
| 431 | 1467 | 1646 | 263 | 180 | 63.65319865 | 1 | 0 | 1 | 1 | 1 | 1 | 1 | 1 | 1 | 1 | 1 | 1 | 1 | 12 |
| 432 | 1174 | 1397 | 264 | 224 | 63.65613553 | 1 | 1 | 1 | 1 | 1 | 1 | 1 | 1 | 1 | 1 | 1 | 1 | 1 | 13 |
| 439 | 2544 | 2837 | 265 | 294 | 70.06802721 | 1 | 1 | 1 | 0 | 1 | 1 | 1 | 1 | 1 | 1 | 1 | 0 | 1 | 11 |
| 440 | 835 | 1000 | 266 | 166 | 68.64184009 | 1 | 1 | 1 | 1 | 1 | 0 | 1 | 1 | 1 | 1 | 1 | 0 | 1 | 11 |
| 440 | 1140 | 1324 | 267 | 185 | 68.77641278 | 1 | 1 | 1 | 1 | 1 | 0 | 1 | 1 | 1 | 1 | 1 | 0 | 1 | 11 |
| 444 | 59 | 231 | 268 | 173 | 78.34593153 | 1 | 1 | 1 | 1 | 1 | 1 | 1 | 1 | 1 | 1 | 1 | 1 | 1 | 13 |
| 445 | 221 | 392 | 269 | 172 | 74.92952784 | 1 | 0 | 1 | 1 | 1 | 1 | 1 | 1 | 1 | 1 | 1 | 1 | 1 | 12 |
| 446 | 55 | 229 | 270 | 175 | 72.64935065 | 1 | 1 | 1 | 1 | 1 | 1 | 1 | 1 | 1 | 0 | 1 | 1 | 1 | 12 |
| 446 | 293 | 449 | 271 | 157 | 73.12294924 | 1 | 1 | 1 | 1 | 1 | 1 | 1 | 1 | 1 | 0 | 1 | 1 | 1 | 12 |
| 446 | 652 | 809 | 272 | 158 | 70.64633679 | 1 | 1 | 1 | 1 | 1 | 1 | 1 | 1 | 1 | 0 | 1 | 1 | 1 | 12 |
| 447 | 103 | 263 | 273 | 161 | 72.53543558 | 1 | 1 | 1 | 1 | 1 | 1 | 1 | 1 | 1 | 1 | 1 | 1 | 1 | 13 |
| 447 | 525 | 797 | 274 | 273 | 71.87001033 | 1 | 1 | 1 | 1 | 1 | 1 | 1 | 1 | 1 | 1 | 1 | 1 | 1 | 13 |
| 448 | 0 | 173 | 275 | 174 | 70.94218042 | 1 | 1 | 1 | 1 | 1 | 1 | 0 | 1 | 1 | 1 | 1 | 1 | 1 | 12 |
| 449 | 630 | 803 | 276 | 174 | 55.61650993 | 1 | 0 | 1 | 1 | 1 | 1 | 1 | 1 | 1 | 1 | 1 | 1 | 1 | 12 |
| 449 | 1888 | 2039 | 277 | 152 | 50.9569378 | 1 | 0 | 1 | 1 | 1 | 1 | 1 | 1 | 1 | 1 | 1 | 1 | 1 | 12 |
| 453 | 1285 | 1450 | 278 | 166 | 68.44651333 | 1 | 0 | 1 | 1 | 1 | 1 | 1 | 1 | 1 | 1 | 1 | 1 | 1 | 12 |
| 454 | 1434 | 1634 | 279 | 201 | 76.55313178 | 1 | 1 | 1 | 1 | 1 | 1 | 1 | 1 | 1 | 1 | 1 | 1 | 1 | 13 |
| 455 | 864 | 1064 | 280 | 201 | 66.78147723 | 1 | 1 | 1 | 1 | 1 | 1 | 1 | 1 | 1 | 1 | 1 | 1 | 1 | 13 |
| 456 | 1325 | 1493 | 281 | 169 | 67.22803823 | 1 | 1 | 1 | 1 | 1 | 1 | 1 | 1 | 1 | 1 | 1 | 1 | 1 | 13 |
| 458 | 3780 | 3950 | 282 | 171 | 60.88959773 | 1 | 0 | 1 | 1 | 1 | 1 | 1 | 1 | 1 | 1 | 1 | 1 | 1 | 12 |
| 459 | 258 | 439 | 283 | 182 | 55.94533671 | 1 | 1 | 1 | 1 | 1 | 1 | 1 | 1 | 1 | 1 | 1 | 1 | 1 | 13 |
| 461 | 1713 | 1864 | 284 | 152 | 75.78748006 | 1 | 1 | 1 | 1 | 1 | 0 | 1 | 1 | 1 | 1 | 1 | 1 | 1 | 12 |
| 464 | 252 | 410 | 285 | 159 | 62.48185776 | 1 | 1 | 1 | 1 | 1 | 1 | 1 | 1 | 1 | 1 | 1 | 1 | 1 | 13 |
| 464 | 827 | 995 | 286 | 169 | 69.7921408 | 1 | 1 | 1 | 1 | 1 | 1 | 1 | 1 | 1 | 1 | 1 | 1 | 1 | 13 |
| 466 | 591 | 746 | 287 | 156 | 65.98679099 | 1 | 1 | 1 | 1 | 1 | 1 | 1 | 0 | 1 | 1 | 1 | 1 | 1 | 12 |
| 466 | 965 | 1148 | 288 | 184 | 71.37681159 | 1 | 1 | 1 | 1 | 1 | 1 | 1 | 0 | 1 | 1 | 1 | 1 | 1 | 12 |
| 466 | 1161 | 1371 | 289 | 211 | 69.06505816 | 1 | 1 | 1 | 1 | 1 | 1 | 1 | 0 | 1 | 1 | 1 | 1 | 1 | 12 |
| 471 | 1227 | 1386 | 290 | 160 | 67.8125 | 1 | 1 | 1 | 1 | 1 | 1 | 1 | 1 | 1 | 1 | 1 | 1 | 1 | 13 |
| 472 | 569 | 743 | 291 | 175 | 56.32900433 | 1 | 1 | 1 | 1 | 1 | 1 | 1 | 1 | 0 | 1 | 1 | 1 | 1 | 12 |
| 473 | 883 | 1068 | 292 | 186 | 62.11306615 | 1 | 0 | 1 | 1 | 1 | 1 | 1 | 1 | 1 | 1 | 1 | 1 | 1 | 12 |
| 476 | 1206 | 1413 | 293 | 208 | 56.08974359 | 1 | 1 | 0 | 1 | 1 | 1 | 1 | 1 | 1 | 1 | 1 | 1 | 1 | 12 |
| 476 | 1706 | 1865 | 294 | 160 | 70.3030303 | 1 | 1 | 0 | 1 | 1 | 1 | 1 | 1 | 1 | 1 | 1 | 1 | 1 | 12 |
| 476 | 2032 | 2213 | 295 | 182 | 65.60939061 | 1 | 1 | 0 | 1 | 1 | 1 | 1 | 1 | 1 | 1 | 1 | 1 | 1 | 12 |
| 478 | 2458 | 2655 | 296 | 198 | 64.74594429 | 1 | 0 | 1 | 1 | 1 | 1 | 1 | 1 | 1 | 1 | 1 | 1 | 1 | 12 |
| 483 | 1257 | 1426 | 297 | 170 | 69.64555053 | 1 | 1 | 1 | 1 | 1 | 1 | 1 | 1 | 1 | 1 | 1 | 1 | 1 | 13 |
| 492 | 896 | 1047 | 298 | 152 | 59.94816587 | 1 | 1 | 1 | 1 | 1 | 0 | 1 | 1 | 1 | 1 | 1 | 1 | 1 | 12 |
| 494 | 3555 | 3773 | 299 | 219 | 68.72146119 | 1 | 0 | 1 | 1 | 1 | 1 | 1 | 1 | 1 | 1 | 1 | 1 | 1 | 12 |
| 495 | 2166 | 2321 | 300 | 156 | 56.31313131 | 1 | 1 | 1 | 1 | 1 | 1 | 1 | 1 | 1 | 1 | 1 | 0 | 1 | 12 |
| 496 | 351 | 544 | 301 | 194 | 72.99762094 | 1 | 1 | 1 | 1 | 1 | 1 | 1 | 1 | 1 | 1 | 1 | 1 | 1 | 13 |
| 496 | 554 | 813 | 302 | 260 | 74.92110454 | 1 | 1 | 1 | 1 | 1 | 1 | 1 | 1 | 1 | 1 | 1 | 1 | 1 | 13 |
| 496 | 816 | 1026 | 303 | 211 | 68.23429335 | 1 | 1 | 1 | 1 | 1 | 1 | 1 | 1 | 1 | 1 | 1 | 1 | 1 | 13 |
| 500 | 285 | 438 | 304 | 154 | 65.11821512 | 1 | 1 | 1 | 1 | 1 | 1 | 1 | 1 | 1 | 1 | 1 | 1 | 1 | 13 |
| 501 | 366 | 529 | 305 | 164 | 62.33924612 | 1 | 1 | 1 | 1 | 1 | 1 | 0 | 0 | 1 | 1 | 1 | 1 | 1 | 11 |
| 501 | 678 | 841 | 306 | 164 | 55.42128603 | 1 | 1 | 1 | 1 | 1 | 1 | 0 | 0 | 1 | 1 | 1 | 1 | 1 | 11 |
| 506 | 814 | 1019 | 307 | 206 | 66.24346527 | 1 | 1 | 1 | 1 | 1 | 1 | 1 | 1 | 1 | 1 | 1 | 1 | 1 | 13 |
| 508 | 829 | 1004 | 308 | 176 | 75.34435262 | 1 | 1 | 1 | 1 | 1 | 1 | 1 | 0 | 1 | 1 | 1 | 1 | 1 | 12 |
| 514 | 972 | 1141 | 309 | 170 | 70.61085973 | 1 | 1 | 1 | 1 | 1 | 1 | 1 | 1 | 1 | 1 | 1 | 1 | 1 | 13 |
| 516 | 328 | 479 | 310 | 152 | 70.63933873 | 1 | 1 | 1 | 1 | 1 | 1 | 1 | 1 | 1 | 1 | 1 | 1 | 1 | 13 |
| 517 | 843 | 1038 | 311 | 196 | 67.22265829 | 1 | 1 | 1 | 1 | 1 | 1 | 1 | 1 | 1 | 1 | 1 | 1 | 1 | 13 |
| 517 | 1043 | 1210 | 312 | 168 | 67.52899878 | 1 | 1 | 1 | 1 | 1 | 1 | 1 | 1 | 1 | 1 | 1 | 1 | 1 | 13 |
| 517 | 1212 | 1410 | 313 | 199 | 74.42339905 | 1 | 1 | 1 | 1 | 1 | 1 | 1 | 1 | 1 | 1 | 1 | 1 | 1 | 13 |
| 525 | 1906 | 2108 | 314 | 203 | 56.85923272 | 1 | 1 | 1 | 1 | 1 | 1 | 1 | 0 | 1 | 1 | 1 | 1 | 1 | 12 |
| 526 | 965 | 1118 | 315 | 154 | 66.2042503 | 1 | 1 | 1 | 1 | 1 | 1 | 1 | 0 | 1 | 1 | 1 | 1 | 1 | 12 |
| 527 | 733 | 935 | 316 | 203 | 55.47842962 | 1 | 0 | 1 | 1 | 1 | 1 | 1 | 1 | 1 | 1 | 1 | 1 | 1 | 12 |
| 529 | 1478 | 1631 | 317 | 154 | 68.44746163 | 1 | 1 | 1 | 1 | 1 | 1 | 1 | 0 | 1 | 1 | 1 | 1 | 1 | 12 |
| 529 | 1803 | 1953 | 318 | 151 | 61.9205298 | 1 | 1 | 1 | 1 | 1 | 1 | 1 | 0 | 1 | 1 | 1 | 1 | 1 | 12 |
| 533 | 124 | 278 | 319 | 155 | 58.76832845 | 1 | 0 | 1 | 1 | 1 | 1 | 1 | 1 | 1 | 1 | 1 | 1 | 1 | 12 |
| 533 | 339 | 532 | 320 | 194 | 53.49109653 | 1 | 0 | 1 | 1 | 1 | 1 | 1 | 1 | 1 | 1 | 1 | 1 | 1 | 12 |
| 533 | 914 | 1064 | 321 | 151 | 56.35159542 | 1 | 0 | 1 | 1 | 1 | 1 | 1 | 1 | 1 | 1 | 1 | 1 | 1 | 12 |
| 537 | 750 | 938 | 322 | 189 | 65.0657984 | 1 | 1 | 1 | 1 | 1 | 1 | 1 | 1 | 1 | 1 | 1 | 1 | 1 | 13 |
| 537 | 1321 | 1475 | 323 | 155 | 70.85194376 | 1 | 1 | 1 | 1 | 1 | 1 | 1 | 1 | 1 | 1 | 1 | 1 | 1 | 13 |
| 538 | 2893 | 3077 | 324 | 185 | 65.88697789 | 1 | 0 | 1 | 1 | 1 | 1 | 1 | 0 | 1 | 1 | 1 | 1 | 1 | 11 |
| 539 | 546 | 722 | 325 | 177 | 77.50253513 | 1 | 1 | 1 | 1 | 1 | 1 | 1 | 1 | 1 | 1 | 1 | 1 | 1 | 13 |
| 540 | 1048 | 1198 | 326 | 151 | 72.55051791 | 1 | 1 | 1 | 1 | 1 | 1 | 1 | 1 | 1 | 1 | 1 | 1 | 1 | 13 |
| 540 | 1489 | 1672 | 327 | 184 | 65.64938685 | 1 | 1 | 1 | 1 | 1 | 1 | 1 | 1 | 1 | 1 | 1 | 1 | 1 | 13 |
| 545 | 1947 | 2127 | 328 | 181 | 66.37368157 | 1 | 0 | 1 | 1 | 1 | 1 | 1 | 1 | 1 | 1 | 1 | 1 | 1 | 12 |
| 545 | 2325 | 2483 | 329 | 159 | 68.94415857 | 1 | 0 | 1 | 1 | 1 | 1 | 1 | 1 | 1 | 1 | 1 | 1 | 1 | 12 |
| 545 | 2534 | 2705 | 330 | 172 | 64.83439042 | 1 | 0 | 1 | 1 | 1 | 1 | 1 | 1 | 1 | 1 | 1 | 1 | 1 | 12 |
| 545 | 2708 | 2864 | 331 | 157 | 60.81837483 | 1 | 0 | 1 | 1 | 1 | 1 | 1 | 1 | 1 | 1 | 1 | 1 | 1 | 12 |
| 547 | 2166 | 2344 | 332 | 179 | 56.01828339 | 1 | 0 | 1 | 1 | 1 | 1 | 0 | 1 | 1 | 1 | 1 | 1 | 1 | 11 |
| 547 | 2940 | 3108 | 333 | 169 | 63.92684239 | 1 | 0 | 1 | 1 | 1 | 1 | 0 | 1 | 1 | 1 | 1 | 1 | 1 | 11 |
| 549 | 2671 | 2838 | 334 | 168 | 68.10064935 | 1 | 0 | 1 | 1 | 1 | 1 | 1 | 1 | 1 | 1 | 1 | 1 | 1 | 12 |
| 550 | 1098 | 1247 | 335 | 150 | 74.42735043 | 1 | 1 | 1 | 1 | 1 | 1 | 1 | 1 | 1 | 1 | 1 | 1 | 1 | 13 |
| 551 | 788 | 970 | 336 | 183 | 71.63373967 | 1 | 1 | 1 | 1 | 1 | 1 | 1 | 1 | 1 | 1 | 1 | 1 | 1 | 13 |
| 553 | 1101 | 1274 | 337 | 174 | 62.6610937 | 1 | 0 | 1 | 1 | 1 | 1 | 1 | 1 | 1 | 1 | 1 | 1 | 1 | 12 |
| 555 | 494 | 660 | 338 | 167 | 72.76356378 | 1 | 1 | 1 | 1 | 1 | 0 | 1 | 1 | 1 | 1 | 1 | 1 | 1 | 12 |
| 556 | 504 | 749 | 339 | 246 | 69.45676275 | 1 | 0 | 1 | 1 | 1 | 1 | 1 | 1 | 1 | 1 | 1 | 1 | 1 | 12 |
| 558 | 309 | 476 | 340 | 168 | 77.06529582 | 1 | 0 | 1 | 1 | 1 | 1 | 1 | 1 | 1 | 1 | 1 | 1 | 1 | 12 |
| 558 | 478 | 634 | 341 | 157 | 75.98919128 | 1 | 0 | 1 | 1 | 1 | 1 | 1 | 1 | 1 | 1 | 1 | 1 | 1 | 12 |
| 558 | 661 | 810 | 342 | 150 | 74.22222222 | 1 | 0 | 1 | 1 | 1 | 1 | 1 | 1 | 1 | 1 | 1 | 1 | 1 | 12 |
| 559 | 1168 | 1370 | 343 | 203 | 55.70980743 | 1 | 0 | 1 | 1 | 1 | 1 | 1 | 1 | 1 | 1 | 1 | 1 | 1 | 12 |
| 560 | 162 | 320 | 344 | 159 | 62.79632317 | 1 | 1 | 1 | 1 | 1 | 1 | 1 | 1 | 1 | 1 | 1 | 1 | 1 | 13 |
| 562 | 450 | 608 | 345 | 159 | 56.15589861 | 1 | 1 | 1 | 1 | 1 | 1 | 1 | 0 | 1 | 1 | 1 | 1 | 1 | 12 |
| 565 | 3280 | 3450 | 346 | 171 | 67.311441 | 1 | 1 | 1 | 1 | 1 | 1 | 1 | 1 | 1 | 1 | 1 | 1 | 1 | 13 |
| 566 | 855 | 1063 | 347 | 209 | 66.29247945 | 1 | 1 | 1 | 1 | 1 | 1 | 1 | 1 | 1 | 1 | 1 | 1 | 1 | 13 |
| 570 | 1478 | 1640 | 348 | 163 | 69.59472021 | 1 | 0 | 1 | 1 | 1 | 1 | 1 | 1 | 1 | 1 | 1 | 1 | 1 | 12 |
| 573 | 2721 | 2901 | 349 | 181 | 69.69117439 | 1 | 1 | 1 | 1 | 1 | 1 | 1 | 1 | 1 | 1 | 1 | 1 | 1 | 13 |
| 573 | 3596 | 3768 | 350 | 173 | 69.14925152 | 1 | 1 | 1 | 1 | 1 | 1 | 1 | 1 | 1 | 1 | 1 | 1 | 1 | 13 |
| 574 | 435 | 648 | 351 | 214 | 64.21639109 | 1 | 1 | 1 | 1 | 1 | 1 | 1 | 1 | 1 | 1 | 1 | 1 | 1 | 13 |
| 576 | 312 | 491 | 352 | 180 | 51.98005698 | 1 | 1 | 1 | 1 | 1 | 1 | 1 | 1 | 1 | 1 | 1 | 1 | 1 | 13 |
| 578 | 403 | 609 | 353 | 207 | 67.04728444 | 1 | 1 | 1 | 1 | 1 | 1 | 1 | 0 | 1 | 1 | 1 | 1 | 1 | 12 |
| 581 | 3270 | 3524 | 354 | 255 | 71.36066548 | 1 | 0 | 1 | 1 | 1 | 1 | 1 | 1 | 1 | 1 | 1 | 1 | 1 | 12 |
| 583 | 1491 | 1647 | 355 | 157 | 79.75665523 | 1 | 1 | 1 | 1 | 1 | 1 | 1 | 1 | 1 | 1 | 1 | 1 | 1 | 13 |
| 584 | 3894 | 4080 | 356 | 187 | 74.29265921 | 1 | 0 | 1 | 1 | 1 | 1 | 0 | 1 | 1 | 1 | 1 | 1 | 1 | 11 |
| 586 | 222 | 380 | 357 | 159 | 67.02877835 | 1 | 0 | 1 | 1 | 1 | 1 | 1 | 1 | 1 | 1 | 1 | 1 | 1 | 12 |
| 590 | 397 | 653 | 358 | 257 | 58.8845655 | 1 | 1 | 1 | 1 | 1 | 1 | 1 | 1 | 1 | 1 | 1 | 1 | 1 | 13 |
| 591 | 0 | 233 | 359 | 234 | 70.77032654 | 1 | 1 | 1 | 1 | 1 | 1 | 1 | 1 | 1 | 1 | 1 | 1 | 1 | 13 |
| 591 | 243 | 784 | 360 | 542 | 73.93320087 | 1 | 1 | 1 | 1 | 1 | 1 | 1 | 1 | 1 | 1 | 1 | 1 | 1 | 13 |
| 595 | 2238 | 2409 | 361 | 172 | 59.22832981 | 1 | 0 | 1 | 1 | 1 | 0 | 1 | 1 | 1 | 1 | 1 | 1 | 1 | 11 |
| 599 | 1754 | 1906 | 362 | 153 | 64.94355318 | 1 | 0 | 1 | 1 | 1 | 1 | 1 | 0 | 1 | 1 | 1 | 1 | 1 | 11 |
| 602 | 474 | 695 | 363 | 222 | 68.42226842 | 1 | 1 | 1 | 1 | 1 | 1 | 1 | 1 | 1 | 1 | 1 | 1 | 1 | 13 |
| 603 | 1844 | 2026 | 364 | 183 | 63.52809304 | 1 | 1 | 1 | 1 | 1 | 1 | 1 | 1 | 1 | 1 | 1 | 1 | 1 | 13 |
| 603 | 3214 | 3403 | 365 | 190 | 69.7705803 | 1 | 1 | 1 | 1 | 1 | 1 | 1 | 1 | 1 | 1 | 1 | 1 | 1 | 13 |
| 604 | 1016 | 1197 | 366 | 182 | 67.26542688 | 1 | 1 | 1 | 1 | 1 | 1 | 1 | 1 | 1 | 1 | 1 | 1 | 1 | 13 |
| 605 | 552 | 727 | 367 | 176 | 67.16942149 | 1 | 1 | 1 | 0 | 1 | 1 | 1 | 0 | 1 | 1 | 1 | 1 | 1 | 11 |
| 606 | 652 | 807 | 368 | 156 | 61.79098679 | 1 | 0 | 1 | 1 | 1 | 1 | 1 | 1 | 1 | 1 | 1 | 1 | 1 | 12 |
| 609 | 2257 | 2417 | 369 | 161 | 62.02420768 | 1 | 1 | 1 | 1 | 1 | 1 | 1 | 1 | 1 | 1 | 1 | 1 | 1 | 13 |
| 609 | 2460 | 2645 | 370 | 186 | 60.84229391 | 1 | 1 | 1 | 1 | 1 | 1 | 1 | 1 | 1 | 1 | 1 | 1 | 1 | 13 |
| 612 | 1974 | 2233 | 371 | 260 | 72.48251748 | 1 | 0 | 1 | 1 | 1 | 1 | 1 | 0 | 1 | 1 | 1 | 1 | 1 | 11 |
| 612 | 2235 | 2408 | 372 | 174 | 76.21734587 | 1 | 0 | 1 | 1 | 1 | 1 | 1 | 0 | 1 | 1 | 1 | 1 | 1 | 11 |
| 612 | 2410 | 2591 | 373 | 182 | 70.15984016 | 1 | 0 | 1 | 1 | 1 | 1 | 1 | 0 | 1 | 1 | 1 | 1 | 1 | 11 |
| 623 | 2497 | 2649 | 374 | 153 | 66.19132501 | 1 | 1 | 0 | 1 | 1 | 1 | 1 | 1 | 1 | 1 | 1 | 1 | 1 | 12 |
| 624 | 342 | 491 | 375 | 150 | 70.23931624 | 1 | 1 | 1 | 1 | 1 | 1 | 1 | 1 | 1 | 1 | 1 | 1 | 1 | 13 |
| 626 | 1005 | 1181 | 376 | 177 | 60.18397798 | 1 | 1 | 1 | 1 | 1 | 1 | 1 | 1 | 1 | 1 | 1 | 1 | 1 | 13 |
| 626 | 1524 | 1697 | 377 | 174 | 59.84379605 | 1 | 1 | 1 | 1 | 1 | 1 | 1 | 1 | 1 | 1 | 1 | 1 | 1 | 13 |
| 629 | 1692 | 1863 | 378 | 172 | 68.08491896 | 1 | 1 | 1 | 1 | 1 | 1 | 1 | 1 | 0 | 1 | 1 | 1 | 1 | 12 |
| 629 | 1865 | 2030 | 379 | 166 | 65.562249 | 1 | 1 | 1 | 1 | 1 | 1 | 1 | 1 | 0 | 1 | 1 | 1 | 1 | 12 |
| 631 | 362 | 527 | 380 | 166 | 55.72289157 | 1 | 0 | 1 | 1 | 1 | 1 | 1 | 1 | 1 | 1 | 1 | 1 | 1 | 12 |
| 635 | 3754 | 3917 | 381 | 164 | 67.22951845 | 1 | 1 | 1 | 1 | 1 | 1 | 1 | 1 | 1 | 1 | 1 | 1 | 1 | 13 |
| 635 | 4240 | 4425 | 382 | 186 | 65.52936311 | 1 | 1 | 1 | 1 | 1 | 1 | 1 | 1 | 1 | 1 | 1 | 1 | 1 | 13 |
| 638 | 1230 | 1450 | 383 | 221 | 70.77967282 | 1 | 1 | 1 | 1 | 1 | 1 | 1 | 1 | 1 | 1 | 1 | 1 | 1 | 13 |
| 639 | 1094 | 1251 | 384 | 158 | 61.69159954 | 1 | 0 | 1 | 1 | 1 | 1 | 1 | 1 | 0 | 1 | 1 | 1 | 1 | 11 |
| 641 | 2619 | 2799 | 385 | 181 | 76.85417713 | 1 | 1 | 1 | 1 | 1 | 0 | 1 | 1 | 1 | 1 | 1 | 1 | 1 | 12 |
| 641 | 2802 | 3019 | 386 | 218 | 72.29635808 | 1 | 1 | 1 | 1 | 1 | 0 | 1 | 1 | 1 | 1 | 1 | 1 | 1 | 12 |
| 641 | 3030 | 3255 | 387 | 226 | 73.57200322 | 1 | 1 | 1 | 1 | 1 | 0 | 1 | 1 | 1 | 1 | 1 | 1 | 1 | 12 |
| 641 | 3275 | 3430 | 388 | 156 | 72.83411033 | 1 | 1 | 1 | 1 | 1 | 0 | 1 | 1 | 1 | 1 | 1 | 1 | 1 | 12 |
| 641 | 3653 | 3851 | 389 | 199 | 66.69712197 | 1 | 1 | 1 | 1 | 1 | 0 | 1 | 1 | 1 | 1 | 1 | 1 | 1 | 12 |
| 643 | 1000 | 1162 | 390 | 163 | 70.40270568 | 1 | 1 | 1 | 1 | 1 | 1 | 1 | 1 | 1 | 1 | 1 | 1 | 1 | 13 |
| 645 | 384 | 548 | 391 | 165 | 58.69605142 | 1 | 1 | 1 | 1 | 1 | 1 | 1 | 0 | 1 | 1 | 1 | 1 | 1 | 12 |
| 650 | 2428 | 2614 | 392 | 187 | 66.56295576 | 1 | 0 | 1 | 1 | 1 | 1 | 0 | 1 | 1 | 1 | 1 | 1 | 1 | 11 |
| 656 | 7620 | 7786 | 393 | 167 | 68.51751043 | 1 | 0 | 1 | 1 | 1 | 1 | 1 | 1 | 1 | 1 | 1 | 1 | 1 | 12 |
| 656 | 8199 | 8385 | 394 | 187 | 75.48209366 | 1 | 0 | 1 | 1 | 1 | 1 | 1 | 1 | 1 | 1 | 1 | 1 | 1 | 12 |
| 656 | 8388 | 8618 | 395 | 231 | 68.43762298 | 1 | 0 | 1 | 1 | 1 | 1 | 1 | 1 | 1 | 1 | 1 | 1 | 1 | 12 |
| 656 | 8620 | 8862 | 396 | 243 | 71.38670657 | 1 | 0 | 1 | 1 | 1 | 1 | 1 | 1 | 1 | 1 | 1 | 1 | 1 | 12 |
| 657 | 929 | 1105 | 397 | 177 | 63.9616504 | 1 | 0 | 1 | 1 | 1 | 1 | 1 | 1 | 1 | 1 | 1 | 1 | 1 | 12 |
| 659 | 1809 | 1962 | 398 | 154 | 59.59858323 | 1 | 0 | 1 | 1 | 1 | 0 | 1 | 1 | 1 | 1 | 1 | 1 | 1 | 11 |
| 659 | 2009 | 2181 | 399 | 173 | 72.07566999 | 1 | 0 | 1 | 1 | 1 | 0 | 1 | 1 | 1 | 1 | 1 | 1 | 1 | 11 |
| 660 | 399 | 582 | 400 | 184 | 52.57107023 | 1 | 1 | 1 | 1 | 1 | 1 | 1 | 1 | 1 | 1 | 1 | 1 | 1 | 13 |
| 662 | 876 | 1027 | 401 | 152 | 70.6539075 | 1 | 0 | 1 | 1 | 1 | 1 | 1 | 1 | 1 | 1 | 1 | 1 | 1 | 12 |
| 662 | 1283 | 1436 | 402 | 154 | 67.04053522 | 1 | 0 | 1 | 1 | 1 | 1 | 1 | 1 | 1 | 1 | 1 | 1 | 1 | 12 |
| 664 | 581 | 796 | 403 | 216 | 62.46438746 | 1 | 1 | 1 | 1 | 1 | 1 | 1 | 1 | 1 | 1 | 1 | 1 | 1 | 13 |
| 665 | 646 | 801 | 404 | 156 | 61.71328671 | 1 | 0 | 1 | 1 | 1 | 0 | 1 | 1 | 1 | 1 | 1 | 1 | 1 | 11 |
| 667 | 885 | 1081 | 405 | 197 | 64.49469312 | 1 | 0 | 1 | 1 | 1 | 1 | 0 | 1 | 1 | 1 | 1 | 1 | 1 | 11 |
| 669 | 2790 | 2965 | 406 | 176 | 63.3953168 | 1 | 0 | 1 | 1 | 1 | 1 | 1 | 1 | 1 | 1 | 1 | 1 | 1 | 12 |
| 669 | 2968 | 3153 | 407 | 186 | 60.5083089 | 1 | 0 | 1 | 1 | 1 | 1 | 1 | 1 | 1 | 1 | 1 | 1 | 1 | 12 |
| 671 | 634 | 806 | 408 | 173 | 60.71466106 | 1 | 0 | 1 | 1 | 1 | 1 | 1 | 1 | 1 | 1 | 0 | 1 | 1 | 11 |
| 672 | 3991 | 4151 | 409 | 161 | 65.24562394 | 1 | 0 | 1 | 1 | 1 | 1 | 1 | 1 | 1 | 1 | 1 | 1 | 1 | 12 |
| 677 | 323 | 482 | 410 | 160 | 53.78205128 | 1 | 1 | 1 | 1 | 1 | 1 | 1 | 1 | 1 | 1 | 1 | 1 | 1 | 13 |
| 679 | 843 | 1005 | 411 | 163 | 77.51297782 | 1 | 1 | 1 | 1 | 1 | 1 | 1 | 1 | 1 | 1 | 1 | 1 | 1 | 13 |
| 683 | 609 | 785 | 412 | 177 | 69.1730868 | 1 | 0 | 1 | 1 | 1 | 0 | 1 | 1 | 1 | 1 | 1 | 1 | 1 | 11 |
| 683 | 817 | 968 | 413 | 152 | 71.41148325 | 1 | 0 | 1 | 1 | 1 | 0 | 1 | 1 | 1 | 1 | 1 | 1 | 1 | 11 |
| 690 | 880 | 1030 | 414 | 151 | 69.44302938 | 1 | 1 | 1 | 1 | 1 | 1 | 1 | 1 | 1 | 1 | 1 | 1 | 1 | 13 |
| 694 | 1159 | 1325 | 415 | 167 | 69.24612314 | 1 | 1 | 1 | 1 | 1 | 1 | 1 | 1 | 1 | 1 | 1 | 1 | 1 | 13 |
| 696 | 2015 | 2252 | 416 | 238 | 73.18563789 | 1 | 1 | 1 | 1 | 1 | 1 | 1 | 1 | 1 | 1 | 1 | 0 | 1 | 12 |
| 696 | 2443 | 2620 | 417 | 178 | 63.89172625 | 1 | 1 | 1 | 1 | 1 | 1 | 1 | 1 | 1 | 1 | 1 | 0 | 1 | 12 |
| 696 | 2643 | 2855 | 418 | 213 | 71.37572912 | 1 | 1 | 1 | 1 | 1 | 1 | 1 | 1 | 1 | 1 | 1 | 0 | 1 | 12 |
| 697 | 309 | 525 | 419 | 217 | 52.28051518 | 1 | 1 | 1 | 1 | 1 | 1 | 1 | 1 | 1 | 1 | 1 | 1 | 1 | 13 |
| 698 | 773 | 945 | 420 | 173 | 66.01856717 | 1 | 1 | 1 | 0 | 1 | 1 | 1 | 1 | 1 | 1 | 1 | 1 | 1 | 12 |
| 700 | 420 | 640 | 421 | 221 | 57.57628495 | 1 | 1 | 1 | 1 | 1 | 1 | 1 | 1 | 1 | 1 | 1 | 1 | 1 | 13 |
| 700 | 854 | 1025 | 422 | 172 | 59.37686345 | 1 | 1 | 1 | 1 | 1 | 1 | 1 | 1 | 1 | 1 | 1 | 1 | 1 | 13 |
| 701 | 377 | 533 | 423 | 157 | 69.13723219 | 1 | 0 | 1 | 1 | 1 | 1 | 1 | 1 | 0 | 1 | 1 | 1 | 1 | 11 |
| 702 | 241 | 395 | 424 | 155 | 67.59305211 | 1 | 1 | 1 | 1 | 1 | 1 | 1 | 1 | 1 | 1 | 1 | 1 | 1 | 13 |
| 704 | 2651 | 2817 | 425 | 167 | 74.58160602 | 1 | 1 | 1 | 1 | 1 | 1 | 1 | 1 | 1 | 1 | 1 | 1 | 1 | 13 |
| 707 | 660 | 865 | 426 | 206 | 58.25865073 | 1 | 1 | 1 | 1 | 1 | 1 | 1 | 1 | 1 | 1 | 1 | 1 | 1 | 13 |
| 709 | 339 | 527 | 427 | 189 | 78.28652829 | 1 | 1 | 1 | 1 | 1 | 1 | 1 | 1 | 1 | 1 | 1 | 1 | 1 | 13 |
| 710 | 2104 | 2255 | 428 | 152 | 67.95255183 | 1 | 0 | 1 | 1 | 1 | 1 | 1 | 1 | 1 | 1 | 1 | 1 | 1 | 12 |
| 714 | 313 | 463 | 429 | 151 | 75.90422822 | 1 | 1 | 1 | 1 | 1 | 1 | 1 | 1 | 1 | 1 | 1 | 1 | 1 | 13 |
| 719 | 552 | 766 | 430 | 215 | 61.86892178 | 1 | 0 | 1 | 1 | 1 | 1 | 1 | 1 | 1 | 1 | 0 | 1 | 1 | 11 |
| 719 | 768 | 1035 | 431 | 268 | 53.83989145 | 1 | 0 | 1 | 1 | 1 | 1 | 1 | 1 | 1 | 1 | 0 | 1 | 1 | 11 |
| 721 | 366 | 522 | 432 | 157 | 68.93456862 | 1 | 1 | 1 | 1 | 1 | 1 | 0 | 1 | 1 | 1 | 1 | 1 | 1 | 12 |
| 721 | 885 | 1131 | 433 | 247 | 72.39602503 | 1 | 1 | 1 | 1 | 1 | 1 | 0 | 1 | 1 | 1 | 1 | 1 | 1 | 12 |
| 723 | 1579 | 1728 | 434 | 150 | 67.21212121 | 1 | 0 | 1 | 1 | 1 | 1 | 1 | 1 | 1 | 1 | 1 | 1 | 1 | 12 |
| 723 | 2407 | 2613 | 435 | 207 | 68.32089006 | 1 | 0 | 1 | 1 | 1 | 1 | 1 | 1 | 1 | 1 | 1 | 1 | 1 | 12 |
| 723 | 2655 | 2808 | 436 | 154 | 75.20661157 | 1 | 0 | 1 | 1 | 1 | 1 | 1 | 1 | 1 | 1 | 1 | 1 | 1 | 12 |
| 723 | 3401 | 3552 | 437 | 152 | 70.49441786 | 1 | 0 | 1 | 1 | 1 | 1 | 1 | 1 | 1 | 1 | 1 | 1 | 1 | 12 |
| 724 | 719 | 871 | 438 | 153 | 66.20122797 | 1 | 0 | 1 | 1 | 1 | 1 | 1 | 1 | 1 | 1 | 1 | 1 | 1 | 12 |
| 727 | 267 | 440 | 439 | 174 | 74.76489028 | 1 | 1 | 1 | 1 | 1 | 1 | 1 | 0 | 1 | 1 | 1 | 1 | 1 | 12 |
| 729 | 3014 | 3175 | 440 | 162 | 62.48035915 | 1 | 0 | 1 | 1 | 1 | 0 | 1 | 1 | 1 | 1 | 1 | 1 | 1 | 11 |
| 730 | 3437 | 3606 | 441 | 170 | 71.80926916 | 1 | 0 | 1 | 1 | 1 | 1 | 1 | 1 | 1 | 1 | 1 | 1 | 1 | 12 |
| 732 | 329 | 506 | 442 | 178 | 54.18794688 | 1 | 0 | 1 | 1 | 1 | 1 | 1 | 1 | 1 | 1 | 1 | 1 | 1 | 12 |
| 732 | 548 | 735 | 443 | 188 | 55.18214055 | 1 | 0 | 1 | 1 | 1 | 1 | 1 | 1 | 1 | 1 | 1 | 1 | 1 | 12 |
| 735 | 999 | 1193 | 444 | 195 | 64.11188811 | 1 | 0 | 1 | 1 | 1 | 1 | 1 | 1 | 0 | 1 | 1 | 1 | 1 | 11 |
| 735 | 1877 | 2027 | 445 | 151 | 66.7910897 | 1 | 0 | 1 | 1 | 1 | 1 | 1 | 1 | 0 | 1 | 1 | 1 | 1 | 11 |
| 737 | 818 | 1009 | 446 | 192 | 76.12689394 | 1 | 0 | 1 | 1 | 1 | 1 | 1 | 1 | 1 | 1 | 1 | 0 | 1 | 11 |
| 738 | 1589 | 1789 | 447 | 201 | 74.03135836 | 1 | 0 | 1 | 1 | 1 | 1 | 1 | 1 | 1 | 1 | 1 | 1 | 1 | 12 |
| 738 | 2407 | 2582 | 448 | 176 | 67.55337466 | 1 | 0 | 1 | 1 | 1 | 1 | 1 | 1 | 1 | 1 | 1 | 1 | 1 | 12 |
| 747 | 58 | 250 | 449 | 193 | 75.39524379 | 1 | 1 | 1 | 1 | 1 | 1 | 1 | 1 | 1 | 1 | 1 | 1 | 1 | 13 |
| 747 | 378 | 544 | 450 | 167 | 74.88868417 | 1 | 1 | 1 | 1 | 1 | 1 | 1 | 1 | 1 | 1 | 1 | 1 | 1 | 13 |
| 747 | 546 | 719 | 451 | 174 | 76.71676982 | 1 | 1 | 1 | 1 | 1 | 1 | 1 | 1 | 1 | 1 | 1 | 1 | 1 | 13 |
| 750 | 459 | 624 | 452 | 166 | 70.44541804 | 1 | 1 | 1 | 1 | 1 | 1 | 0 | 1 | 1 | 1 | 1 | 1 | 1 | 12 |
| 750 | 628 | 860 | 453 | 233 | 74.09285993 | 1 | 1 | 1 | 1 | 1 | 1 | 0 | 1 | 1 | 1 | 1 | 1 | 1 | 12 |
| 750 | 862 | 1083 | 454 | 222 | 68.55719356 | 1 | 1 | 1 | 1 | 1 | 1 | 0 | 1 | 1 | 1 | 1 | 1 | 1 | 12 |
| 751 | 1834 | 2032 | 455 | 199 | 61.59890361 | 1 | 0 | 1 | 1 | 1 | 1 | 0 | 1 | 1 | 1 | 1 | 1 | 1 | 11 |
| 753 | 1049 | 1233 | 456 | 185 | 59.75744976 | 1 | 1 | 1 | 1 | 1 | 1 | 1 | 1 | 1 | 1 | 1 | 1 | 1 | 13 |
| 754 | 139 | 299 | 457 | 161 | 74.0086001 | 1 | 1 | 1 | 1 | 1 | 1 | 1 | 1 | 1 | 1 | 1 | 1 | 1 | 13 |
| 755 | 139 | 333 | 458 | 195 | 67.65285996 | 1 | 1 | 1 | 1 | 1 | 1 | 1 | 1 | 1 | 1 | 1 | 1 | 1 | 13 |
| 759 | 1086 | 1256 | 459 | 171 | 58.88439046 | 1 | 1 | 1 | 1 | 1 | 1 | 1 | 1 | 1 | 1 | 1 | 1 | 1 | 13 |
| 760 | 353 | 516 | 460 | 164 | 67.59053954 | 1 | 1 | 1 | 1 | 1 | 1 | 0 | 1 | 1 | 1 | 1 | 1 | 1 | 12 |
| 761 | 1758 | 1931 | 461 | 174 | 65.98746082 | 1 | 1 | 1 | 1 | 1 | 1 | 1 | 0 | 1 | 1 | 1 | 1 | 1 | 12 |
| 761 | 2078 | 2277 | 462 | 200 | 72.17424242 | 1 | 1 | 1 | 1 | 1 | 1 | 1 | 0 | 1 | 1 | 1 | 1 | 1 | 12 |
| 762 | 315 | 465 | 463 | 151 | 62.30182621 | 1 | 1 | 1 | 1 | 1 | 0 | 1 | 1 | 1 | 1 | 1 | 1 | 1 | 12 |
| 762 | 986 | 1139 | 464 | 154 | 59.14010232 | 1 | 1 | 1 | 1 | 1 | 0 | 1 | 1 | 1 | 1 | 1 | 1 | 1 | 12 |
| 773 | 337 | 932 | 465 | 596 | 67.03782794 | 1 | 1 | 1 | 1 | 1 | 0 | 1 | 1 | 0 | 1 | 1 | 1 | 1 | 11 |
| 780 | 1180 | 1332 | 466 | 153 | 69.01960784 | 1 | 0 | 1 | 1 | 1 | 1 | 0 | 1 | 1 | 1 | 1 | 1 | 1 | 11 |
| 781 | 257 | 443 | 467 | 187 | 56.84217743 | 1 | 1 | 1 | 1 | 1 | 1 | 1 | 1 | 1 | 1 | 1 | 1 | 1 | 13 |
| 784 | 875 | 1035 | 468 | 161 | 62.46000376 | 1 | 1 | 0 | 1 | 1 | 1 | 1 | 1 | 1 | 1 | 1 | 1 | 1 | 12 |
| 786 | 313 | 547 | 469 | 235 | 63.13147845 | 1 | 1 | 1 | 1 | 1 | 1 | 1 | 1 | 1 | 1 | 1 | 1 | 1 | 13 |
| 789 | 2115 | 2284 | 470 | 170 | 66.33484163 | 1 | 1 | 1 | 1 | 1 | 1 | 1 | 1 | 1 | 1 | 1 | 1 | 1 | 13 |
| 791 | 0 | 168 | 471 | 169 | 70.56592323 | 1 | 1 | 1 | 1 | 1 | 1 | 1 | 1 | 1 | 1 | 1 | 1 | 1 | 13 |
| 794 | 564 | 738 | 472 | 175 | 63.95604396 | 1 | 1 | 1 | 1 | 1 | 1 | 1 | 1 | 1 | 1 | 1 | 1 | 1 | 13 |
| 796 | 464 | 629 | 473 | 166 | 61.6739686 | 1 | 0 | 1 | 1 | 1 | 1 | 1 | 1 | 1 | 1 | 1 | 1 | 1 | 12 |
| 798 | 827 | 989 | 474 | 163 | 62.99498048 | 1 | 0 | 1 | 1 | 1 | 1 | 1 | 1 | 1 | 1 | 1 | 1 | 1 | 12 |
| 801 | 2331 | 2530 | 475 | 200 | 71.28030303 | 1 | 1 | 1 | 0 | 1 | 1 | 1 | 1 | 1 | 1 | 1 | 1 | 1 | 12 |
| 807 | 5119 | 5296 | 476 | 178 | 67.19101124 | 1 | 0 | 1 | 1 | 1 | 0 | 1 | 1 | 1 | 0 | 1 | 1 | 1 | 10 |
| 815 | 2789 | 2953 | 477 | 165 | 76.79522498 | 1 | 0 | 1 | 1 | 1 | 1 | 1 | 1 | 1 | 1 | 1 | 1 | 1 | 12 |
| 816 | 2272 | 2438 | 478 | 167 | 60.98469727 | 1 | 0 | 0 | 1 | 1 | 1 | 1 | 0 | 1 | 1 | 1 | 1 | 1 | 10 |
| 821 | 612 | 788 | 479 | 177 | 71.83701421 | 1 | 1 | 1 | 1 | 1 | 1 | 0 | 1 | 1 | 1 | 1 | 1 | 1 | 12 |
| 821 | 814 | 978 | 480 | 165 | 78.80624426 | 1 | 1 | 1 | 1 | 1 | 1 | 0 | 1 | 1 | 1 | 1 | 1 | 1 | 12 |
| 821 | 1004 | 1156 | 481 | 153 | 75.65854625 | 1 | 1 | 1 | 1 | 1 | 1 | 0 | 1 | 1 | 1 | 1 | 1 | 1 | 12 |
| 821 | 1158 | 1331 | 482 | 174 | 68.42563567 | 1 | 1 | 1 | 1 | 1 | 1 | 0 | 1 | 1 | 1 | 1 | 1 | 1 | 12 |
| 828 | 396 | 548 | 483 | 153 | 64.48801743 | 1 | 1 | 1 | 1 | 1 | 1 | 1 | 1 | 1 | 1 | 1 | 0 | 1 | 12 |
| 833 | 423 | 596 | 484 | 174 | 71.43155695 | 1 | 1 | 1 | 0 | 1 | 1 | 1 | 1 | 1 | 1 | 1 | 0 | 1 | 11 |
| 836 | 784 | 971 | 485 | 188 | 78.47356544 | 1 | 0 | 1 | 1 | 1 | 1 | 1 | 1 | 1 | 1 | 1 | 1 | 1 | 12 |
| 836 | 1037 | 1224 | 486 | 188 | 74.96776273 | 1 | 0 | 1 | 1 | 1 | 1 | 1 | 1 | 1 | 1 | 1 | 1 | 1 | 12 |
| 837 | 147 | 324 | 487 | 178 | 74.35308138 | 1 | 1 | 1 | 1 | 1 | 1 | 1 | 1 | 1 | 0 | 1 | 1 | 1 | 12 |
| 842 | 1444 | 1598 | 488 | 155 | 75.54177006 | 1 | 1 | 1 | 1 | 1 | 1 | 1 | 1 | 1 | 1 | 1 | 1 | 1 | 13 |
| 846 | 430 | 579 | 489 | 150 | 73.96581197 | 1 | 1 | 1 | 1 | 1 | 1 | 1 | 1 | 1 | 1 | 1 | 1 | 1 | 13 |
| 848 | 15 | 206 | 490 | 192 | 67.05395299 | 1 | 1 | 1 | 1 | 1 | 1 | 1 | 1 | 1 | 1 | 1 | 1 | 1 | 13 |
| 850 | 523 | 674 | 491 | 152 | 73.33839406 | 1 | 1 | 1 | 1 | 1 | 1 | 1 | 1 | 1 | 1 | 1 | 1 | 1 | 13 |
| 852 | 489 | 1007 | 492 | 519 | 63.82836816 | 1 | 1 | 1 | 1 | 1 | 1 | 1 | 1 | 1 | 1 | 1 | 1 | 1 | 13 |
| 854 | 1881 | 2034 | 493 | 154 | 68.27626919 | 1 | 0 | 1 | 1 | 1 | 1 | 0 | 1 | 1 | 1 | 1 | 1 | 1 | 11 |
| 855 | 457 | 608 | 494 | 152 | 69.00917065 | 1 | 0 | 1 | 1 | 1 | 1 | 1 | 1 | 1 | 1 | 1 | 1 | 1 | 12 |
| 855 | 705 | 882 | 495 | 178 | 56.83520599 | 1 | 0 | 1 | 1 | 1 | 1 | 1 | 1 | 1 | 1 | 1 | 1 | 1 | 12 |
| 856 | 2040 | 2205 | 496 | 166 | 55.68427556 | 1 | 1 | 1 | 1 | 1 | 1 | 1 | 1 | 1 | 1 | 1 | 1 | 1 | 13 |
| 858 | 936 | 1106 | 497 | 171 | 64.51355662 | 1 | 0 | 1 | 1 | 1 | 1 | 1 | 1 | 1 | 1 | 1 | 1 | 1 | 12 |
| 860 | 843 | 995 | 498 | 153 | 59.81230099 | 1 | 1 | 1 | 1 | 1 | 1 | 1 | 1 | 1 | 1 | 1 | 1 | 1 | 13 |
| 865 | 528 | 689 | 499 | 162 | 60.02244669 | 1 | 1 | 1 | 0 | 1 | 1 | 1 | 0 | 1 | 1 | 1 | 1 | 1 | 11 |
| 868 | 1026 | 1186 | 500 | 161 | 67.74964166 | 1 | 1 | 1 | 1 | 1 | 1 | 1 | 1 | 1 | 1 | 1 | 1 | 1 | 13 |
| 871 | 4360 | 4532 | 501 | 173 | 68.16362828 | 1 | 1 | 1 | 1 | 1 | 1 | 1 | 1 | 1 | 1 | 1 | 1 | 1 | 13 |
| 872 | 520 | 671 | 502 | 152 | 75.06977671 | 1 | 1 | 1 | 1 | 1 | 1 | 0 | 1 | 1 | 1 | 1 | 1 | 1 | 12 |
| 875 | 2283 | 2432 | 503 | 150 | 61.22222222 | 1 | 1 | 1 | 1 | 1 | 1 | 1 | 1 | 1 | 1 | 1 | 1 | 1 | 13 |
| 879 | 2442 | 2636 | 504 | 195 | 54.29980276 | 1 | 1 | 1 | 1 | 1 | 1 | 1 | 1 | 1 | 1 | 1 | 1 | 1 | 13 |
| 880 | 1579 | 1747 | 505 | 169 | 62.17569413 | 1 | 1 | 1 | 1 | 1 | 1 | 1 | 1 | 1 | 1 | 1 | 1 | 1 | 13 |
| 881 | 339 | 503 | 506 | 165 | 66.65112665 | 1 | 1 | 1 | 1 | 1 | 1 | 1 | 1 | 1 | 1 | 1 | 1 | 1 | 13 |
| 883 | 1362 | 1529 | 507 | 168 | 60.37087912 | 1 | 1 | 1 | 1 | 1 | 1 | 1 | 1 | 1 | 1 | 1 | 1 | 1 | 13 |
| 884 | 760 | 936 | 508 | 177 | 64.05186151 | 1 | 1 | 1 | 1 | 1 | 1 | 1 | 1 | 1 | 1 | 1 | 1 | 1 | 13 |
| 885 | 723 | 897 | 509 | 175 | 52.7965368 | 1 | 0 | 1 | 1 | 1 | 1 | 1 | 1 | 1 | 1 | 1 | 1 | 1 | 12 |
| 885 | 1389 | 1609 | 510 | 221 | 64.1025641 | 1 | 0 | 1 | 1 | 1 | 1 | 1 | 1 | 1 | 1 | 1 | 1 | 1 | 12 |
| 888 | 491 | 707 | 511 | 217 | 64.34855467 | 1 | 0 | 1 | 1 | 1 | 1 | 1 | 1 | 1 | 1 | 1 | 1 | 1 | 12 |
| 888 | 811 | 1008 | 512 | 198 | 60.8050199 | 1 | 0 | 1 | 1 | 1 | 1 | 1 | 1 | 1 | 1 | 1 | 1 | 1 | 12 |
| 890 | 767 | 931 | 513 | 165 | 56.90753691 | 1 | 1 | 1 | 1 | 1 | 1 | 1 | 1 | 1 | 1 | 1 | 1 | 1 | 13 |
| 890 | 934 | 1103 | 514 | 170 | 59.16289593 | 1 | 1 | 1 | 1 | 1 | 1 | 1 | 1 | 1 | 1 | 1 | 1 | 1 | 13 |
| 891 | 805 | 961 | 515 | 157 | 75.69818716 | 1 | 1 | 1 | 1 | 1 | 1 | 1 | 1 | 1 | 1 | 1 | 1 | 1 | 13 |
| 892 | 560 | 728 | 516 | 169 | 66.90942194 | 1 | 1 | 1 | 1 | 1 | 1 | 1 | 1 | 1 | 1 | 1 | 1 | 1 | 13 |
| 893 | 1123 | 1298 | 517 | 176 | 76.34032634 | 1 | 1 | 1 | 1 | 1 | 1 | 1 | 1 | 1 | 1 | 1 | 1 | 1 | 13 |
| 893 | 1695 | 1888 | 518 | 194 | 67.71081153 | 1 | 1 | 1 | 1 | 1 | 1 | 1 | 1 | 1 | 1 | 1 | 1 | 1 | 13 |
| 898 | 1951 | 2123 | 519 | 173 | 66.14993869 | 1 | 0 | 1 | 1 | 1 | 1 | 1 | 1 | 1 | 1 | 1 | 1 | 1 | 12 |
| 902 | 79 | 281 | 520 | 203 | 65.16357206 | 1 | 1 | 1 | 1 | 1 | 1 | 1 | 1 | 1 | 1 | 1 | 1 | 1 | 13 |
| 904 | 586 | 779 | 521 | 194 | 68.23949247 | 1 | 1 | 1 | 1 | 1 | 1 | 1 | 1 | 1 | 1 | 1 | 1 | 1 | 13 |
| 905 | 454 | 617 | 522 | 164 | 72.61569731 | 1 | 1 | 1 | 1 | 1 | 1 | 1 | 1 | 1 | 1 | 1 | 1 | 1 | 13 |
| 910 | 2929 | 3083 | 523 | 155 | 51.21212121 | 1 | 0 | 1 | 1 | 1 | 1 | 1 | 1 | 1 | 1 | 1 | 1 | 1 | 12 |
| 910 | 5967 | 6120 | 524 | 154 | 54.1322314 | 1 | 0 | 1 | 1 | 1 | 1 | 1 | 1 | 1 | 1 | 1 | 1 | 1 | 12 |
| 910 | 6123 | 6359 | 525 | 237 | 56.43140263 | 1 | 0 | 1 | 1 | 1 | 1 | 1 | 1 | 1 | 1 | 1 | 1 | 1 | 12 |
| 910 | 7626 | 7790 | 526 | 165 | 47.70431589 | 1 | 0 | 1 | 1 | 1 | 1 | 1 | 1 | 1 | 1 | 1 | 1 | 1 | 12 |
| 911 | 645 | 846 | 527 | 202 | 72.37242955 | 1 | 1 | 1 | 1 | 1 | 1 | 1 | 1 | 1 | 1 | 1 | 1 | 1 | 13 |
| 912 | 660 | 854 | 528 | 195 | 54.91064491 | 1 | 0 | 1 | 1 | 1 | 1 | 1 | 1 | 1 | 1 | 1 | 1 | 1 | 12 |
| 912 | 1546 | 1754 | 529 | 209 | 65.08626939 | 1 | 0 | 1 | 1 | 1 | 1 | 1 | 1 | 1 | 1 | 1 | 1 | 1 | 12 |
| 914 | 0 | 162 | 530 | 163 | 74.59564975 | 1 | 0 | 1 | 1 | 1 | 1 | 1 | 1 | 1 | 1 | 1 | 1 | 1 | 12 |
| 914 | 166 | 468 | 531 | 303 | 71.04710471 | 1 | 0 | 1 | 1 | 1 | 1 | 1 | 1 | 1 | 1 | 1 | 1 | 1 | 12 |
| 915 | 0 | 174 | 532 | 175 | 71.67765568 | 1 | 1 | 1 | 1 | 1 | 1 | 1 | 1 | 1 | 1 | 1 | 1 | 1 | 13 |
| 917 | 657 | 847 | 533 | 191 | 68.25321276 | 1 | 1 | 1 | 1 | 1 | 1 | 1 | 1 | 0 | 1 | 1 | 1 | 1 | 12 |
| 920 | 1373 | 1526 | 534 | 154 | 60.46453546 | 1 | 1 | 1 | 1 | 1 | 1 | 1 | 1 | 1 | 1 | 1 | 1 | 1 | 13 |
| 921 | 742 | 943 | 535 | 202 | 69.62427012 | 1 | 1 | 1 | 1 | 1 | 1 | 1 | 1 | 1 | 1 | 1 | 1 | 1 | 13 |
| 921 | 955 | 1121 | 536 | 167 | 69.74512513 | 1 | 1 | 1 | 1 | 1 | 1 | 1 | 1 | 1 | 1 | 1 | 1 | 1 | 13 |
| 923 | 215 | 377 | 537 | 163 | 70.89793642 | 1 | 1 | 1 | 1 | 1 | 1 | 0 | 1 | 0 | 1 | 1 | 1 | 1 | 11 |
| 925 | 142 | 300 | 538 | 159 | 70.13384938 | 1 | 1 | 1 | 1 | 1 | 1 | 1 | 1 | 1 | 1 | 1 | 1 | 1 | 13 |
| 925 | 378 | 530 | 539 | 153 | 63.61655773 | 1 | 1 | 1 | 1 | 1 | 1 | 1 | 1 | 1 | 1 | 1 | 1 | 1 | 13 |
| 926 | 342 | 501 | 540 | 160 | 58.80681818 | 1 | 0 | 1 | 1 | 1 | 1 | 1 | 1 | 1 | 1 | 1 | 1 | 1 | 12 |
| 926 | 615 | 785 | 541 | 171 | 50.78858763 | 1 | 0 | 1 | 1 | 1 | 1 | 1 | 1 | 1 | 1 | 1 | 1 | 1 | 12 |
| 927 | 1461 | 1613 | 542 | 153 | 60.41561924 | 1 | 1 | 1 | 1 | 1 | 1 | 1 | 1 | 1 | 1 | 1 | 1 | 1 | 13 |
| 928 | 1917 | 2066 | 543 | 150 | 53.94949495 | 1 | 0 | 1 | 1 | 1 | 1 | 1 | 1 | 1 | 1 | 1 | 1 | 1 | 12 |
| 934 | 573 | 818 | 544 | 246 | 64.97811132 | 1 | 1 | 1 | 1 | 1 | 1 | 1 | 1 | 1 | 1 | 1 | 1 | 1 | 13 |
| 937 | 525 | 689 | 545 | 165 | 67.7000777 | 1 | 1 | 1 | 1 | 1 | 1 | 1 | 1 | 1 | 1 | 1 | 1 | 1 | 13 |
| 937 | 696 | 862 | 546 | 167 | 76.19376631 | 1 | 1 | 1 | 1 | 1 | 1 | 1 | 1 | 1 | 1 | 1 | 1 | 1 | 13 |
| 938 | 1251 | 1406 | 547 | 156 | 70.46425796 | 1 | 0 | 1 | 1 | 1 | 1 | 1 | 1 | 1 | 1 | 1 | 1 | 1 | 12 |
| 938 | 1470 | 1619 | 548 | 150 | 63.13131313 | 1 | 0 | 1 | 1 | 1 | 1 | 1 | 1 | 1 | 1 | 1 | 1 | 1 | 12 |
| 939 | 404 | 557 | 549 | 154 | 63.78394333 | 1 | 0 | 1 | 1 | 1 | 1 | 1 | 1 | 1 | 1 | 1 | 1 | 1 | 12 |
| 940 | 352 | 516 | 550 | 165 | 71.77156177 | 1 | 1 | 1 | 1 | 1 | 1 | 1 | 1 | 1 | 1 | 1 | 1 | 1 | 13 |
| 942 | 1026 | 1232 | 551 | 207 | 71.4829454 | 1 | 0 | 1 | 1 | 1 | 1 | 1 | 1 | 1 | 1 | 1 | 1 | 1 | 12 |
| 942 | 1883 | 2042 | 552 | 160 | 58.52272727 | 1 | 0 | 1 | 1 | 1 | 1 | 1 | 1 | 1 | 1 | 1 | 1 | 1 | 12 |
| 946 | 1045 | 1213 | 553 | 169 | 69.91351843 | 1 | 1 | 1 | 1 | 1 | 1 | 1 | 1 | 1 | 1 | 1 | 1 | 1 | 13 |
| 949 | 1417 | 1573 | 554 | 157 | 55.29820498 | 1 | 0 | 1 | 1 | 1 | 1 | 1 | 1 | 1 | 1 | 1 | 1 | 1 | 12 |
| 950 | 551 | 701 | 555 | 151 | 60.63847852 | 1 | 1 | 1 | 1 | 1 | 1 | 1 | 1 | 1 | 1 | 1 | 1 | 1 | 13 |
| 950 | 829 | 1001 | 556 | 173 | 63.92470728 | 1 | 1 | 1 | 1 | 1 | 1 | 1 | 1 | 1 | 1 | 1 | 1 | 1 | 13 |
| 952 | 76 | 227 | 557 | 152 | 65.04723347 | 1 | 1 | 1 | 1 | 1 | 1 | 1 | 1 | 1 | 1 | 1 | 1 | 1 | 13 |
| 953 | 329 | 498 | 558 | 170 | 65.88235294 | 1 | 0 | 1 | 1 | 1 | 1 | 1 | 1 | 1 | 1 | 1 | 1 | 1 | 12 |
| 954 | 380 | 641 | 559 | 262 | 55.08905852 | 1 | 1 | 1 | 1 | 1 | 1 | 1 | 1 | 1 | 1 | 1 | 1 | 1 | 13 |
| 954 | 764 | 935 | 560 | 172 | 54.50954085 | 1 | 1 | 1 | 1 | 1 | 1 | 1 | 1 | 1 | 1 | 1 | 1 | 1 | 13 |
| 957 | 795 | 986 | 561 | 192 | 56.1832265 | 1 | 1 | 1 | 1 | 1 | 1 | 1 | 1 | 1 | 1 | 1 | 1 | 1 | 13 |
| 958 | 196 | 368 | 562 | 173 | 71.26871202 | 1 | 1 | 1 | 1 | 1 | 1 | 1 | 1 | 1 | 1 | 1 | 1 | 1 | 13 |
| 959 | 1764 | 1924 | 563 | 161 | 61.12365895 | 1 | 0 | 1 | 1 | 1 | 1 | 1 | 1 | 1 | 1 | 1 | 1 | 1 | 12 |
| 960 | 76 | 226 | 564 | 151 | 68.49210392 | 1 | 1 | 1 | 1 | 1 | 1 | 1 | 1 | 1 | 1 | 1 | 1 | 1 | 13 |
| 961 | 1389 | 1546 | 565 | 158 | 70.31070196 | 1 | 0 | 1 | 1 | 1 | 1 | 1 | 1 | 1 | 1 | 1 | 1 | 1 | 12 |
| 961 | 1590 | 1795 | 566 | 206 | 61.44454251 | 1 | 0 | 1 | 1 | 1 | 1 | 1 | 1 | 1 | 1 | 1 | 1 | 1 | 12 |
| 961 | 1827 | 1994 | 567 | 168 | 68.27200577 | 1 | 0 | 1 | 1 | 1 | 1 | 1 | 1 | 1 | 1 | 1 | 1 | 1 | 12 |
| 970 | 744 | 893 | 568 | 150 | 60.67521368 | 1 | 1 | 1 | 1 | 1 | 1 | 1 | 1 | 1 | 1 | 1 | 1 | 1 | 13 |
| 970 | 1291 | 1442 | 569 | 152 | 75.76754386 | 1 | 1 | 1 | 1 | 1 | 1 | 1 | 1 | 1 | 1 | 1 | 1 | 1 | 13 |
| 971 | 724 | 901 | 570 | 178 | 72.45750504 | 1 | 1 | 1 | 1 | 1 | 1 | 1 | 1 | 1 | 1 | 1 | 1 | 1 | 13 |
| 971 | 903 | 1071 | 571 | 169 | 71.4914277 | 1 | 1 | 1 | 1 | 1 | 1 | 1 | 1 | 1 | 1 | 1 | 1 | 1 | 13 |
| 973 | 825 | 1060 | 572 | 236 | 67.33436055 | 1 | 0 | 1 | 1 | 1 | 1 | 1 | 1 | 1 | 1 | 1 | 1 | 1 | 12 |
| 975 | 719 | 869 | 573 | 151 | 63.16012905 | 1 | 1 | 1 | 1 | 1 | 1 | 1 | 1 | 1 | 1 | 1 | 1 | 1 | 13 |
| 977 | 874 | 1100 | 574 | 227 | 64.02349486 | 1 | 0 | 1 | 1 | 1 | 1 | 1 | 1 | 1 | 1 | 1 | 1 | 1 | 12 |
| 979 | 1554 | 1730 | 575 | 177 | 69.53498479 | 1 | 1 | 1 | 1 | 1 | 1 | 1 | 1 | 1 | 1 | 1 | 1 | 1 | 13 |
| 979 | 1762 | 1967 | 576 | 206 | 66.56708987 | 1 | 1 | 1 | 1 | 1 | 1 | 1 | 1 | 1 | 1 | 1 | 1 | 1 | 13 |
| 979 | 1969 | 2129 | 577 | 161 | 65.84647237 | 1 | 1 | 1 | 1 | 1 | 1 | 1 | 1 | 1 | 1 | 1 | 1 | 1 | 13 |
| 983 | 10269 | 10586 | 578 | 318 | 46.80960549 | 1 | 0 | 1 | 0 | 1 | 1 | 1 | 1 | 1 | 1 | 1 | 1 | 1 | 11 |
| 984 | 1413 | 1568 | 579 | 156 | 63.79176379 | 1 | 0 | 1 | 1 | 1 | 1 | 1 | 1 | 1 | 1 | 1 | 1 | 1 | 12 |
| 986 | 1056 | 1275 | 580 | 220 | 73.25757576 | 1 | 1 | 1 | 1 | 1 | 1 | 1 | 1 | 1 | 1 | 1 | 1 | 1 | 13 |
| 986 | 1277 | 1461 | 581 | 185 | 68.51697852 | 1 | 1 | 1 | 1 | 1 | 1 | 1 | 1 | 1 | 1 | 1 | 1 | 1 | 13 |
| 986 | 1463 | 1747 | 582 | 285 | 67.89473684 | 1 | 1 | 1 | 1 | 1 | 1 | 1 | 1 | 1 | 1 | 1 | 1 | 1 | 13 |
| 987 | 90 | 261 | 583 | 172 | 57.33452594 | 1 | 1 | 1 | 1 | 1 | 1 | 1 | 1 | 1 | 1 | 1 | 1 | 1 | 13 |
| 987 | 543 | 695 | 584 | 153 | 56.41025641 | 1 | 1 | 1 | 1 | 1 | 1 | 1 | 1 | 1 | 1 | 1 | 1 | 1 | 13 |
| 989 | 2160 | 2318 | 585 | 159 | 67.8197065 | 1 | 1 | 1 | 1 | 1 | 1 | 1 | 1 | 1 | 1 | 1 | 1 | 1 | 13 |
| 990 | 966 | 1116 | 586 | 151 | 70.69960944 | 1 | 1 | 1 | 1 | 1 | 1 | 1 | 1 | 1 | 1 | 1 | 1 | 1 | 13 |
| 990 | 1185 | 1428 | 587 | 244 | 73.61286255 | 1 | 1 | 1 | 1 | 1 | 1 | 1 | 1 | 1 | 1 | 1 | 1 | 1 | 13 |
| 996 | 277 | 451 | 588 | 175 | 65.37728938 | 1 | 1 | 1 | 1 | 1 | 1 | 1 | 1 | 1 | 1 | 1 | 1 | 1 | 13 |
| 997 | 1305 | 1466 | 589 | 162 | 69.0487496 | 1 | 1 | 1 | 1 | 1 | 1 | 1 | 1 | 1 | 1 | 1 | 1 | 1 | 13 |
| 998 | 1291 | 1446 | 590 | 156 | 62.73310023 | 1 | 0 | 1 | 1 | 1 | 1 | 1 | 1 | 1 | 1 | 1 | 1 | 1 | 12 |
| 999 | 272 | 444 | 591 | 173 | 74.83325923 | 1 | 1 | 1 | 1 | 1 | 1 | 1 | 1 | 1 | 1 | 1 | 1 | 1 | 13 |
| 999 | 447 | 647 | 592 | 201 | 73.31292257 | 1 | 1 | 1 | 1 | 1 | 1 | 1 | 1 | 1 | 1 | 1 | 1 | 1 | 13 |
| 999 | 1213 | 1367 | 593 | 155 | 59.75186104 | 1 | 1 | 1 | 1 | 1 | 1 | 1 | 1 | 1 | 1 | 1 | 1 | 1 | 13 |
| 1001 | 2122 | 2274 | 594 | 153 | 69.73353444 | 1 | 1 | 1 | 1 | 1 | 1 | 1 | 1 | 1 | 1 | 1 | 1 | 1 | 13 |
| 1003 | 211 | 390 | 595 | 180 | 67.77777778 | 1 | 1 | 1 | 1 | 1 | 1 | 1 | 1 | 1 | 1 | 1 | 1 | 1 | 13 |
| 1006 | 3765 | 3987 | 596 | 223 | 67.3257236 | 1 | 0 | 1 | 1 | 1 | 1 | 1 | 1 | 1 | 1 | 1 | 1 | 1 | 12 |
| 1006 | 4656 | 4817 | 597 | 162 | 73.31649832 | 1 | 0 | 1 | 1 | 1 | 1 | 1 | 1 | 1 | 1 | 1 | 1 | 1 | 12 |
| 1007 | 1077 | 1257 | 598 | 181 | 63.17428428 | 1 | 1 | 1 | 1 | 1 | 1 | 0 | 1 | 1 | 0 | 1 | 1 | 1 | 11 |
| 1007 | 1259 | 1435 | 599 | 177 | 63.0097586 | 1 | 1 | 1 | 1 | 1 | 1 | 0 | 1 | 1 | 0 | 1 | 1 | 1 | 11 |
| 1007 | 1529 | 1886 | 600 | 358 | 71.02590147 | 1 | 1 | 1 | 1 | 1 | 1 | 0 | 1 | 1 | 0 | 1 | 1 | 1 | 11 |
| 1008 | 4282 | 4431 | 601 | 150 | 69.45299145 | 1 | 1 | 1 | 1 | 1 | 1 | 1 | 1 | 1 | 1 | 1 | 1 | 1 | 13 |
| 1010 | 1035 | 1225 | 602 | 191 | 69.08614945 | 1 | 0 | 1 | 1 | 1 | 1 | 1 | 1 | 1 | 1 | 1 | 1 | 1 | 12 |
| 1010 | 1231 | 1430 | 603 | 200 | 71.16666667 | 1 | 0 | 1 | 1 | 1 | 1 | 1 | 1 | 1 | 1 | 1 | 1 | 1 | 12 |
| 1010 | 1432 | 1662 | 604 | 231 | 65.89269317 | 1 | 0 | 1 | 1 | 1 | 1 | 1 | 1 | 1 | 1 | 1 | 1 | 1 | 12 |
| 1011 | 333 | 484 | 605 | 152 | 51.95374801 | 1 | 0 | 1 | 1 | 1 | 1 | 1 | 1 | 1 | 1 | 1 | 1 | 1 | 12 |
| 1013 | 348 | 516 | 606 | 169 | 66.04805451 | 1 | 0 | 1 | 1 | 1 | 1 | 1 | 1 | 1 | 1 | 1 | 1 | 1 | 12 |
| 1013 | 706 | 865 | 607 | 160 | 70.43560606 | 1 | 0 | 1 | 1 | 1 | 1 | 1 | 1 | 1 | 1 | 1 | 1 | 1 | 12 |
| 1014 | 719 | 879 | 608 | 161 | 50.35037426 | 1 | 1 | 1 | 1 | 1 | 1 | 1 | 1 | 1 | 1 | 1 | 1 | 1 | 13 |
| 1014 | 1149 | 1310 | 609 | 162 | 53.76701488 | 1 | 1 | 1 | 1 | 1 | 1 | 1 | 1 | 1 | 1 | 1 | 1 | 1 | 13 |
| 1017 | 265 | 442 | 610 | 178 | 53.4284068 | 1 | 1 | 1 | 1 | 1 | 1 | 1 | 1 | 1 | 1 | 1 | 1 | 1 | 13 |
| 1018 | 275 | 434 | 611 | 160 | 59.87980769 | 1 | 1 | 1 | 1 | 1 | 1 | 1 | 1 | 1 | 1 | 1 | 1 | 1 | 13 |
| 1018 | 436 | 594 | 612 | 159 | 62.71569102 | 1 | 1 | 1 | 1 | 1 | 1 | 1 | 1 | 1 | 1 | 1 | 1 | 1 | 13 |
| 1019 | 99 | 286 | 613 | 188 | 64.35488271 | 1 | 1 | 1 | 1 | 1 | 1 | 1 | 1 | 1 | 1 | 1 | 1 | 1 | 13 |
| 1019 | 288 | 523 | 614 | 236 | 66.24837027 | 1 | 1 | 1 | 1 | 1 | 1 | 1 | 1 | 1 | 1 | 1 | 1 | 1 | 13 |
| 1020 | 171 | 329 | 615 | 159 | 57.41376024 | 1 | 0 | 1 | 1 | 1 | 1 | 1 | 1 | 1 | 1 | 1 | 1 | 1 | 12 |
| 1021 | 168 | 377 | 616 | 210 | 72.21001221 | 1 | 1 | 1 | 1 | 1 | 1 | 1 | 1 | 1 | 1 | 1 | 1 | 1 | 13 |
| 1021 | 455 | 714 | 617 | 260 | 71.06015779 | 1 | 1 | 1 | 1 | 1 | 1 | 1 | 1 | 1 | 1 | 1 | 1 | 1 | 13 |
| 1021 | 730 | 885 | 618 | 156 | 71.43326759 | 1 | 1 | 1 | 1 | 1 | 1 | 1 | 1 | 1 | 1 | 1 | 1 | 1 | 13 |
| 1022 | 1026 | 1200 | 619 | 175 | 64.73260073 | 1 | 1 | 1 | 1 | 1 | 1 | 1 | 1 | 1 | 1 | 1 | 1 | 1 | 13 |
| 1023 | 621 | 821 | 620 | 201 | 55.05804312 | 1 | 0 | 1 | 1 | 1 | 1 | 1 | 1 | 1 | 1 | 1 | 1 | 1 | 12 |
| 1032 | 1563 | 1785 | 621 | 223 | 55.46165344 | 1 | 1 | 1 | 1 | 1 | 1 | 1 | 1 | 1 | 1 | 1 | 1 | 1 | 13 |
| 1042 | 85 | 259 | 622 | 175 | 72.7032967 | 1 | 1 | 1 | 1 | 1 | 1 | 1 | 1 | 1 | 1 | 1 | 1 | 1 | 13 |
| 1042 | 358 | 550 | 623 | 193 | 76.56436827 | 1 | 1 | 1 | 1 | 1 | 1 | 1 | 1 | 1 | 1 | 1 | 1 | 1 | 13 |
| 1043 | 597 | 811 | 624 | 215 | 62.64270613 | 1 | 0 | 1 | 1 | 1 | 1 | 1 | 1 | 1 | 1 | 1 | 1 | 1 | 12 |
| 1045 | 409 | 625 | 625 | 217 | 70.46501885 | 1 | 1 | 1 | 1 | 1 | 0 | 1 | 0 | 1 | 1 | 1 | 1 | 1 | 11 |
| 1046 | 318 | 494 | 626 | 177 | 68.20223091 | 1 | 1 | 1 | 1 | 1 | 1 | 1 | 1 | 1 | 1 | 1 | 1 | 1 | 13 |
| 1046 | 511 | 710 | 627 | 200 | 66.52564103 | 1 | 1 | 1 | 1 | 1 | 1 | 1 | 1 | 1 | 1 | 1 | 1 | 1 | 13 |
| 1046 | 816 | 997 | 628 | 182 | 66.94139194 | 1 | 1 | 1 | 1 | 1 | 1 | 1 | 1 | 1 | 1 | 1 | 1 | 1 | 13 |
| 1046 | 1000 | 1155 | 629 | 156 | 67.2008547 | 1 | 1 | 1 | 1 | 1 | 1 | 1 | 1 | 1 | 1 | 1 | 1 | 1 | 13 |
| 1048 | 1389 | 1549 | 630 | 161 | 65.79073101 | 1 | 1 | 1 | 1 | 1 | 1 | 1 | 1 | 1 | 1 | 1 | 1 | 1 | 13 |
| 1048 | 1833 | 1983 | 631 | 151 | 73.43352012 | 1 | 1 | 1 | 1 | 1 | 1 | 1 | 1 | 1 | 1 | 1 | 1 | 1 | 13 |
| 1050 | 578 | 735 | 632 | 158 | 61.31126258 | 1 | 1 | 1 | 1 | 1 | 1 | 1 | 1 | 1 | 1 | 1 | 1 | 1 | 13 |
| 1051 | 264 | 435 | 633 | 172 | 63.57334526 | 1 | 1 | 1 | 1 | 1 | 1 | 1 | 1 | 1 | 1 | 1 | 1 | 1 | 13 |
| 1051 | 850 | 1015 | 634 | 166 | 65.03707136 | 1 | 1 | 1 | 1 | 1 | 1 | 1 | 1 | 1 | 1 | 1 | 1 | 1 | 13 |
| 1051 | 1147 | 1309 | 635 | 163 | 68.08242882 | 1 | 1 | 1 | 1 | 1 | 1 | 1 | 1 | 1 | 1 | 1 | 1 | 1 | 13 |
| 1052 | 1269 | 1449 | 636 | 181 | 70.52698683 | 1 | 1 | 1 | 1 | 1 | 1 | 1 | 1 | 1 | 1 | 1 | 1 | 1 | 13 |
| 1052 | 2542 | 2727 | 637 | 186 | 61.67631652 | 1 | 1 | 1 | 1 | 1 | 1 | 1 | 1 | 1 | 1 | 1 | 1 | 1 | 13 |
| 1056 | 1059 | 1233 | 638 | 175 | 64.90842491 | 1 | 1 | 1 | 1 | 1 | 1 | 1 | 1 | 1 | 1 | 1 | 1 | 1 | 13 |
| 1059 | 603 | 778 | 639 | 176 | 69.22348485 | 1 | 1 | 1 | 1 | 1 | 1 | 1 | 1 | 1 | 1 | 1 | 1 | 1 | 13 |
| 1065 | 507 | 677 | 640 | 171 | 59.52166742 | 1 | 1 | 1 | 1 | 1 | 1 | 1 | 1 | 1 | 1 | 1 | 1 | 1 | 13 |
| 1066 | 50 | 234 | 641 | 185 | 65.78655579 | 1 | 1 | 1 | 1 | 1 | 1 | 1 | 1 | 1 | 1 | 1 | 1 | 1 | 13 |
| 1068 | 805 | 957 | 642 | 153 | 66.12200436 | 1 | 1 | 1 | 1 | 1 | 1 | 1 | 1 | 1 | 1 | 1 | 1 | 1 | 13 |
| 1068 | 960 | 1123 | 643 | 164 | 64.14165103 | 1 | 1 | 1 | 1 | 1 | 1 | 1 | 1 | 1 | 1 | 1 | 1 | 1 | 13 |
| 1071 | 90 | 253 | 644 | 164 | 68.98843027 | 1 | 1 | 1 | 1 | 1 | 1 | 1 | 1 | 1 | 1 | 1 | 1 | 1 | 13 |
| 1072 | 814 | 1046 | 645 | 233 | 76.5544184 | 1 | 1 | 1 | 1 | 1 | 1 | 1 | 1 | 1 | 1 | 1 | 1 | 1 | 13 |
| 1075 | 232 | 398 | 646 | 167 | 70.81222171 | 1 | 1 | 1 | 1 | 1 | 1 | 1 | 1 | 1 | 1 | 1 | 1 | 1 | 13 |
| 1076 | 0 | 248 | 647 | 249 | 69.33374524 | 1 | 1 | 1 | 1 | 1 | 1 | 1 | 1 | 1 | 1 | 1 | 1 | 1 | 13 |
| 1078 | 425 | 591 | 648 | 167 | 76.0206859 | 1 | 1 | 1 | 1 | 1 | 1 | 0 | 1 | 1 | 1 | 1 | 1 | 1 | 12 |
| 1079 | 654 | 835 | 649 | 182 | 56.46020646 | 1 | 0 | 1 | 1 | 1 | 1 | 1 | 1 | 1 | 1 | 1 | 1 | 1 | 12 |
| 1082 | 607 | 770 | 650 | 164 | 60.20168856 | 1 | 1 | 1 | 1 | 1 | 1 | 1 | 1 | 1 | 1 | 1 | 1 | 1 | 13 |
| 1083 | 147 | 341 | 651 | 195 | 67.31097962 | 1 | 1 | 1 | 1 | 1 | 1 | 1 | 1 | 1 | 1 | 1 | 1 | 1 | 13 |
| 1084 | 775 | 941 | 652 | 167 | 70.9964686 | 1 | 1 | 1 | 1 | 1 | 1 | 1 | 1 | 1 | 1 | 1 | 1 | 1 | 13 |
| 1086 | 1593 | 1768 | 653 | 176 | 70.42871901 | 1 | 0 | 1 | 1 | 1 | 1 | 1 | 1 | 1 | 1 | 1 | 1 | 1 | 12 |
| 1087 | 1888 | 2048 | 654 | 161 | 60.60606061 | 1 | 0 | 1 | 1 | 1 | 1 | 1 | 1 | 1 | 1 | 1 | 1 | 1 | 12 |
| 1087 | 2083 | 2251 | 655 | 169 | 62.7129281 | 1 | 0 | 1 | 1 | 1 | 1 | 1 | 1 | 1 | 1 | 1 | 1 | 1 | 12 |
| 1090 | 1811 | 1964 | 656 | 154 | 57.15118215 | 1 | 1 | 1 | 1 | 1 | 1 | 1 | 1 | 1 | 1 | 1 | 1 | 1 | 13 |
| 1091 | 650 | 804 | 657 | 155 | 71.53846154 | 1 | 1 | 1 | 1 | 1 | 1 | 1 | 1 | 1 | 1 | 1 | 1 | 1 | 13 |
| 1093 | 1195 | 1348 | 658 | 154 | 64.49383949 | 1 | 1 | 1 | 1 | 1 | 1 | 1 | 1 | 1 | 1 | 1 | 1 | 1 | 13 |
| 1095 | 644 | 803 | 659 | 160 | 72.32371795 | 1 | 1 | 1 | 1 | 1 | 1 | 1 | 1 | 1 | 1 | 1 | 1 | 1 | 13 |
| 1098 | 1614 | 1768 | 660 | 155 | 74.54545455 | 1 | 0 | 1 | 1 | 1 | 1 | 1 | 1 | 1 | 1 | 1 | 1 | 1 | 12 |
| 1099 | 30 | 221 | 661 | 192 | 64.97729701 | 1 | 1 | 1 | 1 | 1 | 1 | 1 | 1 | 1 | 1 | 1 | 1 | 1 | 13 |
| 1099 | 224 | 396 | 662 | 173 | 68.22291389 | 1 | 1 | 1 | 1 | 1 | 1 | 1 | 1 | 1 | 1 | 1 | 1 | 1 | 13 |
| 1100 | 124 | 275 | 663 | 152 | 70.80802969 | 1 | 1 | 1 | 1 | 1 | 1 | 1 | 1 | 1 | 1 | 1 | 1 | 1 | 13 |
| 1102 | 544 | 770 | 664 | 227 | 73.25200497 | 1 | 1 | 1 | 1 | 1 | 1 | 1 | 1 | 1 | 1 | 1 | 1 | 1 | 13 |
| 1103 | 469 | 623 | 665 | 155 | 64.22663358 | 1 | 1 | 1 | 1 | 1 | 1 | 1 | 1 | 1 | 1 | 1 | 1 | 1 | 13 |
| 1106 | 1721 | 1894 | 666 | 174 | 63.22709857 | 1 | 0 | 1 | 1 | 1 | 1 | 1 | 1 | 1 | 1 | 1 | 1 | 1 | 12 |
| 1111 | 1479 | 1662 | 667 | 184 | 65.96673254 | 1 | 0 | 1 | 1 | 1 | 1 | 1 | 1 | 1 | 1 | 1 | 1 | 1 | 12 |
| 1112 | 338 | 509 | 668 | 172 | 64.43053071 | 1 | 1 | 1 | 1 | 1 | 1 | 1 | 1 | 1 | 1 | 1 | 1 | 1 | 13 |
| 1114 | 588 | 782 | 669 | 195 | 69.60552268 | 1 | 1 | 1 | 1 | 1 | 1 | 1 | 1 | 1 | 1 | 1 | 1 | 1 | 13 |
| 1114 | 864 | 1352 | 670 | 489 | 72.03607572 | 1 | 1 | 1 | 1 | 1 | 1 | 1 | 1 | 1 | 1 | 1 | 1 | 1 | 13 |
| 1114 | 1356 | 1952 | 671 | 597 | 72.98458102 | 1 | 1 | 1 | 1 | 1 | 1 | 1 | 1 | 1 | 1 | 1 | 1 | 1 | 13 |
| 1117 | 2652 | 2837 | 672 | 186 | 58.03193223 | 1 | 0 | 1 | 1 | 1 | 1 | 1 | 1 | 1 | 1 | 1 | 1 | 1 | 12 |
| 1118 | 361 | 576 | 673 | 216 | 51.35921178 | 1 | 1 | 1 | 1 | 1 | 1 | 1 | 1 | 1 | 1 | 1 | 1 | 1 | 13 |
| 1119 | 3777 | 3938 | 674 | 162 | 73.41722064 | 1 | 1 | 1 | 1 | 1 | 1 | 1 | 1 | 1 | 1 | 1 | 1 | 1 | 13 |
| 1120 | 51 | 215 | 675 | 165 | 64.36674437 | 1 | 1 | 1 | 1 | 1 | 1 | 1 | 1 | 1 | 1 | 1 | 1 | 1 | 13 |
| 1122 | 208 | 360 | 676 | 153 | 76.8728004 | 1 | 1 | 1 | 1 | 1 | 1 | 1 | 1 | 1 | 1 | 1 | 1 | 1 | 13 |
| 1122 | 362 | 542 | 677 | 181 | 75.68352458 | 1 | 1 | 1 | 1 | 1 | 1 | 1 | 1 | 1 | 1 | 1 | 1 | 1 | 13 |
| 1123 | 289 | 582 | 678 | 294 | 66.67102739 | 1 | 1 | 1 | 1 | 1 | 1 | 1 | 1 | 1 | 1 | 1 | 1 | 1 | 13 |
| 1123 | 584 | 806 | 679 | 223 | 53.30573761 | 1 | 1 | 1 | 1 | 1 | 1 | 1 | 1 | 1 | 1 | 1 | 1 | 1 | 13 |
| 1126 | 923 | 1111 | 680 | 189 | 70.36358703 | 1 | 1 | 1 | 1 | 1 | 1 | 1 | 1 | 1 | 1 | 1 | 1 | 1 | 13 |
| 1128 | 906 | 1127 | 681 | 222 | 62.04088704 | 1 | 1 | 1 | 1 | 1 | 1 | 1 | 1 | 1 | 1 | 1 | 1 | 1 | 13 |
| 1130 | 96 | 341 | 682 | 246 | 63.10667652 | 1 | 0 | 1 | 1 | 1 | 1 | 1 | 1 | 1 | 1 | 1 | 1 | 1 | 12 |
| 1131 | 3522 | 3700 | 683 | 179 | 66.45179774 | 1 | 1 | 1 | 1 | 1 | 1 | 1 | 1 | 1 | 1 | 1 | 1 | 1 | 13 |
| 1135 | 1686 | 1835 | 684 | 150 | 60.75757576 | 1 | 0 | 1 | 1 | 1 | 1 | 1 | 1 | 1 | 1 | 1 | 1 | 1 | 12 |
| 1135 | 1848 | 2198 | 685 | 351 | 67.23215057 | 1 | 0 | 1 | 1 | 1 | 1 | 1 | 1 | 1 | 1 | 1 | 1 | 1 | 12 |
| 1136 | 1247 | 1441 | 686 | 195 | 75.35353535 | 1 | 0 | 1 | 1 | 1 | 1 | 1 | 1 | 1 | 1 | 1 | 1 | 1 | 12 |
| 1137 | 362 | 516 | 687 | 155 | 59.85938792 | 1 | 1 | 1 | 1 | 1 | 1 | 1 | 1 | 1 | 1 | 1 | 1 | 1 | 13 |
| 1139 | 498 | 680 | 688 | 183 | 69.1326888 | 1 | 1 | 1 | 1 | 1 | 1 | 1 | 1 | 1 | 1 | 1 | 1 | 1 | 13 |
| 1146 | 261 | 450 | 689 | 190 | 64.40620783 | 1 | 1 | 1 | 1 | 1 | 1 | 1 | 1 | 1 | 1 | 1 | 1 | 1 | 13 |
| 1148 | 2084 | 2265 | 690 | 182 | 52.12287712 | 1 | 0 | 1 | 1 | 1 | 1 | 1 | 1 | 1 | 1 | 1 | 1 | 1 | 12 |
| 1148 | 2610 | 2815 | 691 | 206 | 52.11091497 | 1 | 0 | 1 | 1 | 1 | 1 | 1 | 1 | 1 | 1 | 1 | 1 | 1 | 12 |
| 1149 | 240 | 400 | 692 | 161 | 72.14379823 | 1 | 0 | 1 | 1 | 1 | 1 | 1 | 1 | 1 | 1 | 1 | 1 | 1 | 12 |
| 1152 | 477 | 646 | 693 | 170 | 72.02111614 | 1 | 1 | 1 | 1 | 1 | 1 | 1 | 1 | 1 | 1 | 1 | 1 | 1 | 13 |
| 1154 | 777 | 1094 | 694 | 318 | 57.85687059 | 1 | 1 | 1 | 1 | 1 | 1 | 1 | 0 | 1 | 1 | 1 | 1 | 1 | 12 |
| 1156 | 180 | 343 | 695 | 164 | 67.43277048 | 1 | 1 | 1 | 1 | 1 | 1 | 1 | 1 | 1 | 1 | 1 | 1 | 1 | 13 |
| 1159 | 720 | 946 | 696 | 227 | 71.19877186 | 1 | 1 | 1 | 1 | 1 | 1 | 1 | 0 | 1 | 1 | 1 | 1 | 1 | 12 |
| 1159 | 948 | 1126 | 697 | 179 | 70.51802946 | 1 | 1 | 1 | 1 | 1 | 1 | 1 | 0 | 1 | 1 | 1 | 1 | 1 | 12 |
| 1160 | 1018 | 1214 | 698 | 197 | 66.20460758 | 1 | 1 | 1 | 1 | 1 | 1 | 1 | 1 | 1 | 1 | 1 | 1 | 1 | 13 |
| 1161 | 1266 | 1433 | 699 | 168 | 62.12121212 | 1 | 0 | 1 | 1 | 1 | 1 | 1 | 1 | 1 | 1 | 1 | 1 | 1 | 12 |
| 1161 | 1629 | 1792 | 700 | 164 | 59.85772358 | 1 | 0 | 1 | 1 | 1 | 1 | 1 | 1 | 1 | 1 | 1 | 1 | 1 | 12 |
| 1161 | 2437 | 2612 | 701 | 176 | 64.35089532 | 1 | 0 | 1 | 1 | 1 | 1 | 1 | 1 | 1 | 1 | 1 | 1 | 1 | 12 |
| 1164 | 1058 | 1237 | 702 | 180 | 62.84848485 | 1 | 0 | 1 | 1 | 1 | 1 | 1 | 0 | 1 | 1 | 1 | 1 | 1 | 11 |
| 1166 | 220 | 424 | 703 | 205 | 79.71857411 | 1 | 1 | 1 | 1 | 1 | 1 | 1 | 1 | 1 | 1 | 1 | 1 | 1 | 13 |
| 1167 | 1895 | 2046 | 704 | 152 | 67.68724696 | 1 | 1 | 1 | 1 | 1 | 1 | 1 | 1 | 1 | 1 | 1 | 1 | 1 | 13 |
| 1173 | 489 | 660 | 705 | 172 | 65.07223397 | 1 | 0 | 1 | 1 | 1 | 1 | 1 | 1 | 1 | 1 | 1 | 1 | 1 | 12 |
| 1173 | 684 | 897 | 706 | 214 | 61.20787312 | 1 | 0 | 1 | 1 | 1 | 1 | 1 | 1 | 1 | 1 | 1 | 1 | 1 | 12 |
| 1174 | 969 | 1133 | 707 | 165 | 66.28593629 | 1 | 1 | 1 | 1 | 1 | 1 | 1 | 1 | 1 | 1 | 1 | 1 | 1 | 13 |
| 1178 | 1185 | 1372 | 708 | 188 | 67.59410802 | 1 | 1 | 1 | 1 | 1 | 1 | 1 | 1 | 1 | 1 | 1 | 1 | 1 | 13 |
| 1179 | 1936 | 2113 | 709 | 178 | 71.21866897 | 1 | 1 | 1 | 1 | 1 | 1 | 1 | 1 | 1 | 1 | 1 | 1 | 1 | 13 |
| 1180 | 403 | 623 | 710 | 221 | 58.806126 | 1 | 1 | 1 | 1 | 1 | 1 | 1 | 1 | 1 | 1 | 1 | 1 | 1 | 13 |
| 1180 | 625 | 777 | 711 | 153 | 58.43807609 | 1 | 1 | 1 | 1 | 1 | 1 | 1 | 1 | 1 | 1 | 1 | 1 | 1 | 13 |
| 1183 | 1929 | 2145 | 712 | 217 | 60.67588326 | 1 | 0 | 1 | 1 | 1 | 1 | 1 | 1 | 1 | 1 | 1 | 1 | 1 | 12 |
| 1183 | 2148 | 2308 | 713 | 161 | 65.59382646 | 1 | 0 | 1 | 1 | 1 | 1 | 1 | 1 | 1 | 1 | 1 | 1 | 1 | 12 |
| 1191 | 408 | 635 | 714 | 228 | 71.99730094 | 1 | 1 | 1 | 1 | 1 | 1 | 1 | 1 | 1 | 1 | 1 | 1 | 1 | 13 |
| 1191 | 1771 | 1935 | 715 | 165 | 70.51282051 | 1 | 1 | 1 | 1 | 1 | 1 | 1 | 1 | 1 | 1 | 1 | 1 | 1 | 13 |
| 1192 | 634 | 806 | 716 | 173 | 67.55595079 | 1 | 1 | 1 | 1 | 1 | 1 | 1 | 1 | 1 | 1 | 1 | 1 | 1 | 13 |
| 1192 | 818 | 971 | 717 | 154 | 67.87379287 | 1 | 1 | 1 | 1 | 1 | 1 | 1 | 1 | 1 | 1 | 1 | 1 | 1 | 13 |
| 1197 | 276 | 427 | 718 | 152 | 71.79027113 | 1 | 1 | 1 | 1 | 1 | 1 | 0 | 1 | 1 | 1 | 1 | 1 | 1 | 12 |
| 1197 | 431 | 607 | 719 | 177 | 69.73121041 | 1 | 1 | 1 | 1 | 1 | 1 | 0 | 1 | 1 | 1 | 1 | 1 | 1 | 12 |
| 1202 | 1263 | 1449 | 720 | 187 | 67.37967914 | 1 | 1 | 1 | 1 | 1 | 1 | 1 | 1 | 1 | 1 | 1 | 1 | 1 | 13 |
| 1204 | 387 | 608 | 721 | 222 | 71.58697159 | 1 | 1 | 1 | 1 | 1 | 1 | 1 | 1 | 1 | 1 | 1 | 1 | 1 | 13 |
| 1208 | 1887 | 2073 | 722 | 187 | 50.64445359 | 1 | 1 | 1 | 1 | 1 | 1 | 1 | 1 | 1 | 1 | 1 | 1 | 1 | 13 |
| 1210 | 726 | 916 | 723 | 191 | 67.57954088 | 1 | 1 | 1 | 1 | 1 | 1 | 1 | 1 | 1 | 1 | 1 | 1 | 1 | 13 |
| 1211 | 110 | 331 | 724 | 222 | 73.46962347 | 1 | 1 | 1 | 1 | 1 | 1 | 1 | 1 | 1 | 1 | 1 | 1 | 1 | 13 |
| 1213 | 1107 | 1264 | 725 | 158 | 59.68546222 | 1 | 0 | 1 | 1 | 1 | 1 | 1 | 1 | 1 | 1 | 1 | 1 | 1 | 12 |
| 1216 | 666 | 824 | 726 | 159 | 55.91840026 | 1 | 1 | 1 | 1 | 1 | 1 | 1 | 1 | 1 | 1 | 1 | 1 | 1 | 13 |
| 1217 | 200 | 354 | 727 | 155 | 65.25227461 | 1 | 1 | 1 | 1 | 1 | 1 | 1 | 1 | 1 | 1 | 1 | 1 | 1 | 13 |
| 1218 | 846 | 998 | 728 | 153 | 53.85453327 | 1 | 1 | 1 | 1 | 1 | 1 | 1 | 1 | 1 | 1 | 1 | 1 | 1 | 13 |
| 1221 | 126 | 276 | 729 | 151 | 62.65919511 | 1 | 1 | 1 | 1 | 1 | 1 | 1 | 1 | 1 | 1 | 1 | 1 | 1 | 13 |
| 1223 | 1026 | 1191 | 730 | 166 | 71.46768894 | 1 | 0 | 1 | 1 | 1 | 1 | 1 | 1 | 1 | 1 | 1 | 1 | 1 | 12 |
| 1225 | 1558 | 1728 | 731 | 171 | 59.89721779 | 1 | 0 | 1 | 1 | 1 | 1 | 1 | 1 | 1 | 1 | 1 | 1 | 1 | 12 |
| 1225 | 1935 | 2153 | 732 | 219 | 69.15040819 | 1 | 0 | 1 | 1 | 1 | 1 | 1 | 1 | 1 | 1 | 1 | 1 | 1 | 12 |
| 1226 | 622 | 806 | 733 | 185 | 65.36445536 | 1 | 0 | 1 | 1 | 1 | 1 | 1 | 1 | 1 | 1 | 1 | 1 | 1 | 12 |
| 1229 | 2385 | 2553 | 734 | 169 | 56.42542861 | 1 | 1 | 1 | 1 | 1 | 1 | 1 | 1 | 1 | 1 | 1 | 1 | 1 | 13 |
| 1229 | 2721 | 3005 | 735 | 285 | 69.98650472 | 1 | 1 | 1 | 1 | 1 | 1 | 1 | 1 | 1 | 1 | 1 | 1 | 1 | 13 |
| 1232 | 184 | 360 | 736 | 177 | 70.72287411 | 1 | 1 | 1 | 1 | 1 | 1 | 1 | 1 | 1 | 1 | 1 | 1 | 1 | 13 |
| 1235 | 274 | 441 | 737 | 168 | 65.98748474 | 1 | 1 | 1 | 1 | 1 | 1 | 1 | 1 | 1 | 1 | 1 | 1 | 1 | 13 |
| 1235 | 445 | 656 | 738 | 212 | 67.32583454 | 1 | 1 | 1 | 1 | 1 | 1 | 1 | 1 | 1 | 1 | 1 | 1 | 1 | 13 |
| 1236 | 3816 | 4079 | 739 | 264 | 77.14876033 | 1 | 1 | 1 | 1 | 1 | 0 | 1 | 1 | 0 | 1 | 1 | 1 | 1 | 11 |
| 1244 | 140 | 290 | 740 | 151 | 64.37425709 | 1 | 1 | 1 | 1 | 1 | 1 | 1 | 1 | 1 | 1 | 1 | 1 | 1 | 13 |
| 1246 | 1745 | 1896 | 741 | 152 | 60.49641148 | 1 | 1 | 1 | 1 | 1 | 1 | 1 | 0 | 1 | 1 | 1 | 1 | 1 | 12 |
| 1247 | 458 | 664 | 742 | 207 | 70.19075932 | 1 | 1 | 1 | 1 | 1 | 1 | 1 | 1 | 1 | 1 | 1 | 1 | 1 | 13 |
| 1249 | 93 | 262 | 743 | 170 | 70.54298643 | 1 | 1 | 1 | 1 | 1 | 1 | 1 | 1 | 1 | 1 | 1 | 1 | 1 | 13 |
| 1254 | 1489 | 1645 | 744 | 157 | 74.96325331 | 1 | 1 | 1 | 1 | 1 | 1 | 1 | 1 | 1 | 1 | 1 | 1 | 1 | 13 |
| 1257 | 1479 | 1629 | 745 | 151 | 66.74308032 | 1 | 1 | 1 | 1 | 1 | 1 | 1 | 1 | 1 | 1 | 1 | 1 | 1 | 13 |
| 1257 | 1783 | 1956 | 746 | 174 | 65.31830239 | 1 | 1 | 1 | 1 | 1 | 1 | 1 | 1 | 1 | 1 | 1 | 1 | 1 | 13 |
| 1258 | 1386 | 1558 | 747 | 173 | 71.63183637 | 1 | 1 | 1 | 1 | 1 | 1 | 1 | 1 | 1 | 1 | 1 | 1 | 1 | 13 |
| 1258 | 1831 | 1997 | 748 | 167 | 74.49715953 | 1 | 1 | 1 | 1 | 1 | 1 | 1 | 1 | 1 | 1 | 1 | 1 | 1 | 13 |
| 1259 | 482 | 669 | 749 | 188 | 69.98090562 | 1 | 1 | 1 | 1 | 1 | 1 | 1 | 1 | 1 | 1 | 1 | 1 | 1 | 13 |
| 1259 | 672 | 899 | 750 | 228 | 67.89248763 | 1 | 1 | 1 | 1 | 1 | 1 | 1 | 1 | 1 | 1 | 1 | 1 | 1 | 13 |
| 1260 | 486 | 647 | 751 | 162 | 70.07755619 | 1 | 1 | 1 | 1 | 1 | 1 | 1 | 1 | 1 | 1 | 1 | 1 | 1 | 13 |
| 1261 | 1065 | 1260 | 752 | 196 | 69.97526283 | 1 | 0 | 1 | 1 | 1 | 1 | 1 | 1 | 1 | 1 | 1 | 1 | 1 | 12 |
| 1262 | 944 | 1143 | 753 | 200 | 63.78205128 | 1 | 1 | 1 | 1 | 1 | 1 | 1 | 1 | 1 | 1 | 1 | 1 | 1 | 13 |
| 1262 | 1160 | 1324 | 754 | 165 | 63.15462315 | 1 | 1 | 1 | 1 | 1 | 1 | 1 | 1 | 1 | 1 | 1 | 1 | 1 | 13 |
| 1262 | 1331 | 1559 | 755 | 229 | 67.22091591 | 1 | 1 | 1 | 1 | 1 | 1 | 1 | 1 | 1 | 1 | 1 | 1 | 1 | 13 |
| 1263 | 2004 | 2185 | 756 | 182 | 73.74292374 | 1 | 0 | 1 | 1 | 1 | 1 | 1 | 1 | 1 | 1 | 1 | 1 | 1 | 12 |
| 1265 | 411 | 567 | 757 | 157 | 72.47635592 | 1 | 1 | 1 | 1 | 1 | 1 | 1 | 1 | 1 | 0 | 1 | 1 | 1 | 12 |
| 1265 | 587 | 764 | 758 | 178 | 70.77800477 | 1 | 1 | 1 | 1 | 1 | 1 | 1 | 1 | 1 | 0 | 1 | 1 | 1 | 12 |
| 1268 | 1122 | 1282 | 759 | 161 | 69.74040452 | 1 | 1 | 1 | 1 | 1 | 1 | 1 | 1 | 1 | 1 | 1 | 1 | 1 | 13 |
| 1268 | 2041 | 2223 | 760 | 183 | 63.09373686 | 1 | 1 | 1 | 1 | 1 | 1 | 1 | 1 | 1 | 1 | 1 | 1 | 1 | 13 |
| 1273 | 1240 | 1437 | 761 | 198 | 73.2970733 | 1 | 1 | 1 | 1 | 1 | 1 | 1 | 1 | 1 | 1 | 1 | 1 | 1 | 13 |
| 1274 | 714 | 869 | 762 | 156 | 48.60289283 | 1 | 1 | 1 | 1 | 1 | 1 | 1 | 1 | 1 | 1 | 1 | 1 | 1 | 13 |
| 1274 | 942 | 1115 | 763 | 174 | 48.74005305 | 1 | 1 | 1 | 1 | 1 | 1 | 1 | 1 | 1 | 1 | 1 | 1 | 1 | 13 |
| 1278 | 1008 | 1167 | 764 | 160 | 76.41098485 | 1 | 1 | 1 | 1 | 1 | 1 | 1 | 1 | 1 | 1 | 1 | 0 | 1 | 12 |
| 1279 | 926 | 1099 | 765 | 174 | 68.68686869 | 1 | 1 | 1 | 1 | 1 | 1 | 0 | 1 | 1 | 1 | 1 | 1 | 1 | 12 |
| 1279 | 1125 | 1330 | 766 | 206 | 65.67372757 | 1 | 1 | 1 | 1 | 1 | 1 | 0 | 1 | 1 | 1 | 1 | 1 | 1 | 12 |
| 1284 | 576 | 745 | 767 | 170 | 74.24585219 | 1 | 1 | 1 | 1 | 1 | 1 | 1 | 1 | 1 | 1 | 1 | 1 | 1 | 13 |
| 1287 | 2063 | 2373 | 768 | 311 | 58.41784154 | 1 | 1 | 1 | 1 | 1 | 1 | 1 | 1 | 1 | 1 | 1 | 1 | 1 | 13 |
| 1292 | 405 | 602 | 769 | 198 | 51.97487697 | 1 | 1 | 1 | 1 | 1 | 1 | 1 | 1 | 1 | 1 | 1 | 1 | 1 | 13 |
| 1293 | 1302 | 1466 | 770 | 165 | 54.32789433 | 1 | 1 | 1 | 1 | 1 | 1 | 1 | 1 | 1 | 1 | 1 | 1 | 1 | 13 |
| 1294 | 339 | 497 | 771 | 159 | 60.25347818 | 1 | 0 | 1 | 1 | 1 | 1 | 1 | 1 | 1 | 1 | 1 | 1 | 1 | 12 |
| 1296 | 3802 | 3961 | 772 | 160 | 72.90277778 | 1 | 0 | 1 | 1 | 1 | 1 | 0 | 1 | 1 | 0 | 1 | 1 | 1 | 10 |
| 1297 | 269 | 429 | 773 | 161 | 71.69931518 | 1 | 1 | 1 | 1 | 1 | 1 | 1 | 1 | 1 | 1 | 1 | 1 | 1 | 13 |
| 1300 | 658 | 816 | 774 | 159 | 67.52943074 | 1 | 1 | 1 | 1 | 1 | 1 | 1 | 1 | 1 | 1 | 1 | 1 | 1 | 13 |
| 1301 | 3998 | 4153 | 775 | 156 | 71.40861275 | 1 | 1 | 1 | 1 | 1 | 1 | 1 | 1 | 1 | 1 | 1 | 1 | 1 | 13 |
| 1304 | 276 | 441 | 776 | 166 | 65.63526835 | 1 | 0 | 1 | 1 | 1 | 1 | 1 | 1 | 1 | 1 | 1 | 1 | 1 | 12 |
| 1304 | 1125 | 1276 | 777 | 152 | 69.28827751 | 1 | 0 | 1 | 1 | 1 | 1 | 1 | 1 | 1 | 1 | 1 | 1 | 1 | 12 |
| 1304 | 1440 | 1659 | 778 | 220 | 74.80716253 | 1 | 0 | 1 | 1 | 1 | 1 | 1 | 1 | 1 | 1 | 1 | 1 | 1 | 12 |
| 1305 | 561 | 727 | 779 | 167 | 53.78334241 | 1 | 1 | 1 | 1 | 1 | 1 | 0 | 1 | 1 | 1 | 1 | 1 | 1 | 12 |
| 1306 | 1781 | 1958 | 780 | 178 | 69.32243786 | 1 | 0 | 1 | 1 | 1 | 1 | 1 | 1 | 1 | 1 | 1 | 1 | 1 | 12 |
| 1307 | 918 | 1118 | 781 | 201 | 60.66462559 | 1 | 1 | 1 | 1 | 1 | 1 | 1 | 1 | 1 | 1 | 1 | 1 | 1 | 13 |
| 1309 | 1381 | 1538 | 782 | 158 | 67.24277832 | 1 | 1 | 1 | 1 | 1 | 1 | 1 | 1 | 1 | 1 | 1 | 1 | 1 | 13 |
| 1312 | 330 | 491 | 783 | 162 | 71.96106363 | 1 | 1 | 1 | 1 | 1 | 1 | 1 | 1 | 1 | 1 | 1 | 1 | 1 | 13 |
| 1314 | 1159 | 1330 | 784 | 172 | 76.17024448 | 1 | 1 | 1 | 1 | 1 | 1 | 1 | 1 | 1 | 1 | 1 | 1 | 1 | 13 |
| 1315 | 1461 | 1623 | 785 | 163 | 58.89570552 | 1 | 1 | 1 | 1 | 1 | 1 | 0 | 0 | 1 | 1 | 1 | 1 | 1 | 11 |
| 1317 | 679 | 850 | 786 | 172 | 66.74120453 | 1 | 1 | 1 | 1 | 1 | 1 | 1 | 1 | 1 | 1 | 1 | 1 | 1 | 13 |
| 1318 | 237 | 404 | 787 | 168 | 56.92918193 | 1 | 1 | 1 | 1 | 1 | 1 | 1 | 1 | 1 | 1 | 1 | 1 | 1 | 13 |
| 1320 | 864 | 1079 | 788 | 216 | 70.00237417 | 1 | 1 | 1 | 1 | 1 | 1 | 1 | 1 | 1 | 1 | 1 | 1 | 1 | 13 |
| 1320 | 1172 | 1326 | 789 | 155 | 78.32919768 | 1 | 1 | 1 | 1 | 1 | 1 | 1 | 1 | 1 | 1 | 1 | 1 | 1 | 13 |
| 1320 | 1328 | 1481 | 790 | 154 | 77.08957709 | 1 | 1 | 1 | 1 | 1 | 1 | 1 | 1 | 1 | 1 | 1 | 1 | 1 | 13 |
| 1321 | 1920 | 2071 | 791 | 152 | 59.81858054 | 1 | 0 | 1 | 1 | 1 | 1 | 1 | 1 | 1 | 1 | 1 | 1 | 1 | 12 |
| 1321 | 2181 | 2357 | 792 | 177 | 66.87211094 | 1 | 0 | 1 | 1 | 1 | 1 | 1 | 1 | 1 | 1 | 1 | 1 | 1 | 12 |
| 1321 | 2724 | 2879 | 793 | 156 | 69.5027195 | 1 | 0 | 1 | 1 | 1 | 1 | 1 | 1 | 1 | 1 | 1 | 1 | 1 | 12 |
| 1325 | 623 | 822 | 794 | 200 | 69.79487179 | 1 | 1 | 1 | 1 | 1 | 1 | 1 | 1 | 1 | 1 | 1 | 1 | 1 | 13 |
| 1325 | 988 | 1176 | 795 | 189 | 59.91724325 | 1 | 1 | 1 | 1 | 1 | 1 | 1 | 1 | 1 | 1 | 1 | 1 | 1 | 13 |
| 1327 | 1754 | 1944 | 796 | 191 | 73.66089408 | 1 | 1 | 1 | 1 | 1 | 1 | 1 | 1 | 1 | 1 | 1 | 1 | 1 | 13 |
| 1331 | 664 | 854 | 797 | 191 | 59.26970063 | 1 | 1 | 1 | 1 | 1 | 1 | 1 | 1 | 1 | 1 | 1 | 1 | 1 | 13 |
| 1333 | 1082 | 1251 | 798 | 170 | 63.08377897 | 1 | 0 | 1 | 1 | 1 | 1 | 1 | 1 | 1 | 1 | 1 | 1 | 1 | 12 |
| 1336 | 1215 | 1376 | 799 | 162 | 72.90497568 | 1 | 0 | 1 | 1 | 1 | 1 | 1 | 1 | 1 | 1 | 1 | 1 | 1 | 12 |
| 1343 | 1485 | 1661 | 800 | 177 | 72.55606917 | 1 | 0 | 1 | 1 | 1 | 1 | 1 | 1 | 1 | 1 | 1 | 1 | 1 | 12 |
| 1344 | 315 | 692 | 801 | 378 | 61.02292769 | 1 | 0 | 1 | 1 | 1 | 1 | 1 | 1 | 1 | 1 | 1 | 1 | 1 | 12 |
| 1344 | 861 | 1030 | 802 | 170 | 56.4973262 | 1 | 0 | 1 | 1 | 1 | 1 | 1 | 1 | 1 | 1 | 1 | 1 | 1 | 12 |
| 1344 | 1655 | 1871 | 803 | 217 | 60.7875995 | 1 | 0 | 1 | 1 | 1 | 1 | 1 | 1 | 1 | 1 | 1 | 1 | 1 | 12 |
| 1344 | 2449 | 2644 | 804 | 196 | 58.0550402 | 1 | 0 | 1 | 1 | 1 | 1 | 1 | 1 | 1 | 1 | 1 | 1 | 1 | 12 |
| 1346 | 603 | 785 | 805 | 183 | 73.37307501 | 1 | 1 | 1 | 1 | 1 | 1 | 0 | 1 | 1 | 1 | 1 | 1 | 1 | 12 |
| 1347 | 216 | 373 | 806 | 158 | 58.91829689 | 1 | 0 | 1 | 1 | 1 | 1 | 1 | 1 | 1 | 1 | 1 | 1 | 1 | 12 |
| 1348 | 4002 | 4228 | 807 | 227 | 68.34868509 | 1 | 0 | 1 | 1 | 1 | 1 | 1 | 1 | 1 | 1 | 1 | 1 | 1 | 12 |
| 1349 | 227 | 386 | 808 | 160 | 60.16826923 | 1 | 1 | 1 | 1 | 1 | 1 | 1 | 1 | 1 | 1 | 1 | 1 | 1 | 13 |
| 1350 | 525 | 676 | 809 | 152 | 69.28981107 | 1 | 1 | 1 | 1 | 1 | 1 | 1 | 1 | 1 | 1 | 1 | 1 | 1 | 13 |
| 1350 | 1052 | 1203 | 810 | 152 | 65.82321188 | 1 | 1 | 1 | 1 | 1 | 1 | 1 | 1 | 1 | 1 | 1 | 1 | 1 | 13 |
| 1355 | 915 | 1074 | 811 | 160 | 54.3349359 | 1 | 1 | 1 | 1 | 1 | 1 | 1 | 1 | 1 | 1 | 1 | 1 | 1 | 13 |
| 1356 | 473 | 626 | 812 | 154 | 62.02963703 | 1 | 1 | 1 | 1 | 1 | 1 | 1 | 1 | 1 | 1 | 1 | 1 | 1 | 13 |
| 1357 | 1809 | 2033 | 813 | 225 | 65.51566952 | 1 | 1 | 1 | 1 | 1 | 1 | 1 | 1 | 1 | 1 | 1 | 1 | 1 | 13 |
| 1357 | 2052 | 2214 | 814 | 163 | 68.34985056 | 1 | 1 | 1 | 1 | 1 | 1 | 1 | 1 | 1 | 1 | 1 | 1 | 1 | 13 |
| 1357 | 3846 | 4025 | 815 | 180 | 70.61965812 | 1 | 1 | 1 | 1 | 1 | 1 | 1 | 1 | 1 | 1 | 1 | 1 | 1 | 13 |
| 1359 | 744 | 902 | 816 | 159 | 57.70037091 | 1 | 1 | 1 | 1 | 1 | 1 | 1 | 1 | 1 | 1 | 1 | 1 | 1 | 13 |
| 1366 | 1593 | 1775 | 817 | 183 | 55.21609538 | 1 | 0 | 1 | 1 | 1 | 1 | 1 | 1 | 1 | 1 | 1 | 1 | 1 | 12 |
| 1371 | 1143 | 1296 | 818 | 154 | 63.99433899 | 1 | 1 | 1 | 1 | 1 | 1 | 1 | 1 | 1 | 1 | 1 | 1 | 1 | 13 |
| 1371 | 1332 | 1549 | 819 | 218 | 58.48623853 | 1 | 1 | 1 | 1 | 1 | 1 | 1 | 1 | 1 | 1 | 1 | 1 | 1 | 13 |
| 1373 | 1479 | 1628 | 820 | 150 | 49.63636364 | 1 | 1 | 1 | 1 | 1 | 1 | 0 | 1 | 1 | 1 | 1 | 1 | 1 | 12 |
| 1377 | 759 | 928 | 821 | 170 | 59.13273002 | 1 | 1 | 1 | 1 | 1 | 1 | 1 | 1 | 1 | 1 | 1 | 1 | 1 | 13 |
| 1383 | 291 | 506 | 822 | 216 | 60.51756885 | 1 | 1 | 1 | 1 | 1 | 1 | 1 | 1 | 1 | 1 | 1 | 1 | 1 | 13 |
| 1387 | 351 | 540 | 823 | 190 | 64.73684211 | 1 | 1 | 1 | 1 | 1 | 1 | 1 | 1 | 1 | 1 | 1 | 1 | 1 | 13 |
| 1392 | 576 | 772 | 824 | 197 | 60.15228426 | 1 | 1 | 1 | 1 | 1 | 1 | 1 | 1 | 1 | 1 | 1 | 1 | 1 | 13 |
| 1396 | 3507 | 3694 | 825 | 188 | 68.58478401 | 1 | 0 | 1 | 1 | 1 | 1 | 1 | 1 | 1 | 1 | 1 | 1 | 1 | 12 |
| 1396 | 3983 | 4136 | 826 | 154 | 58.40220386 | 1 | 0 | 1 | 1 | 1 | 1 | 1 | 1 | 1 | 1 | 1 | 1 | 1 | 12 |
| 1399 | 435 | 875 | 827 | 441 | 65.91371591 | 1 | 1 | 1 | 1 | 1 | 1 | 1 | 1 | 1 | 1 | 1 | 1 | 1 | 13 |
| 1399 | 877 | 1062 | 828 | 186 | 68.43810312 | 1 | 1 | 1 | 1 | 1 | 1 | 1 | 1 | 1 | 1 | 1 | 1 | 1 | 13 |
| 1399 | 1175 | 1535 | 829 | 361 | 72.11094538 | 1 | 1 | 1 | 1 | 1 | 1 | 1 | 1 | 1 | 1 | 1 | 1 | 1 | 13 |
| 1401 | 576 | 726 | 830 | 151 | 63.98369842 | 1 | 1 | 1 | 1 | 1 | 1 | 1 | 1 | 1 | 1 | 1 | 1 | 1 | 13 |
| 1401 | 729 | 893 | 831 | 165 | 57.32711733 | 1 | 1 | 1 | 1 | 1 | 1 | 1 | 1 | 1 | 1 | 1 | 1 | 1 | 13 |
| 1407 | 544 | 695 | 832 | 152 | 68.39114833 | 1 | 0 | 1 | 1 | 1 | 1 | 1 | 1 | 1 | 1 | 1 | 1 | 1 | 12 |
| 1407 | 766 | 957 | 833 | 192 | 65.7354798 | 1 | 0 | 1 | 1 | 1 | 1 | 1 | 1 | 1 | 1 | 1 | 1 | 1 | 12 |
| 1408 | 255 | 442 | 834 | 188 | 66.95308238 | 1 | 1 | 1 | 1 | 1 | 1 | 1 | 1 | 1 | 1 | 1 | 1 | 1 | 13 |
| 1410 | 333 | 539 | 835 | 207 | 45.05759941 | 1 | 1 | 1 | 1 | 1 | 1 | 1 | 1 | 1 | 1 | 1 | 1 | 1 | 13 |
| 1411 | 1402 | 1565 | 836 | 164 | 52.11069418 | 1 | 1 | 1 | 1 | 1 | 1 | 1 | 1 | 1 | 1 | 1 | 1 | 1 | 13 |
| 1412 | 1417 | 1574 | 837 | 158 | 59.67587265 | 1 | 0 | 1 | 1 | 1 | 1 | 1 | 1 | 1 | 1 | 1 | 1 | 1 | 12 |
| 1414 | 449 | 723 | 838 | 275 | 74.84848485 | 1 | 0 | 1 | 1 | 1 | 1 | 1 | 1 | 1 | 1 | 1 | 1 | 1 | 12 |
| 1414 | 725 | 909 | 839 | 185 | 70.32760033 | 1 | 0 | 1 | 1 | 1 | 1 | 1 | 1 | 1 | 1 | 1 | 1 | 1 | 12 |
| 1417 | 795 | 949 | 840 | 155 | 61.37303557 | 1 | 1 | 1 | 1 | 1 | 1 | 1 | 1 | 1 | 1 | 1 | 1 | 1 | 13 |
| 1418 | 1395 | 1566 | 841 | 172 | 69.93887895 | 1 | 1 | 1 | 1 | 1 | 1 | 1 | 1 | 1 | 1 | 1 | 1 | 1 | 13 |
| 1419 | 129 | 427 | 842 | 299 | 60.11920075 | 1 | 1 | 1 | 1 | 1 | 1 | 1 | 1 | 1 | 1 | 1 | 1 | 1 | 13 |
| 1420 | 405 | 577 | 843 | 173 | 64.56943827 | 1 | 1 | 1 | 1 | 1 | 1 | 1 | 1 | 1 | 1 | 1 | 1 | 1 | 13 |
| 1421 | 404 | 602 | 844 | 199 | 60.53987888 | 1 | 1 | 1 | 1 | 1 | 1 | 1 | 1 | 1 | 1 | 1 | 1 | 1 | 13 |
| 1421 | 615 | 809 | 845 | 195 | 63.86587771 | 1 | 1 | 1 | 1 | 1 | 1 | 1 | 1 | 1 | 1 | 1 | 1 | 1 | 13 |
| 1427 | 948 | 1136 | 846 | 189 | 70.52638719 | 1 | 1 | 1 | 1 | 1 | 1 | 1 | 1 | 1 | 1 | 1 | 1 | 1 | 13 |
| 1429 | 984 | 1158 | 847 | 175 | 73.61172161 | 1 | 1 | 1 | 1 | 1 | 1 | 1 | 1 | 1 | 1 | 1 | 1 | 1 | 13 |
| 1430 | 135 | 305 | 848 | 171 | 63.93762183 | 1 | 1 | 1 | 1 | 1 | 1 | 1 | 1 | 1 | 1 | 1 | 1 | 1 | 13 |
| 1438 | 1530 | 1711 | 849 | 182 | 54.7036297 | 1 | 0 | 1 | 1 | 1 | 1 | 1 | 1 | 1 | 1 | 1 | 1 | 1 | 12 |
| 1438 | 1713 | 1891 | 850 | 179 | 60.69070594 | 1 | 0 | 1 | 1 | 1 | 1 | 1 | 1 | 1 | 1 | 1 | 1 | 1 | 12 |
| 1446 | 2281 | 2441 | 851 | 161 | 51.14667941 | 1 | 1 | 1 | 1 | 1 | 1 | 1 | 1 | 1 | 1 | 1 | 1 | 1 | 13 |
| 1447 | 785 | 976 | 852 | 192 | 61.51175214 | 1 | 1 | 1 | 1 | 1 | 1 | 1 | 1 | 1 | 1 | 1 | 1 | 1 | 13 |
| 1449 | 935 | 1214 | 853 | 280 | 55.65934066 | 1 | 1 | 1 | 1 | 1 | 1 | 1 | 1 | 1 | 1 | 1 | 1 | 1 | 13 |
| 1450 | 9227 | 9393 | 854 | 167 | 62.26456179 | 1 | 0 | 1 | 1 | 1 | 1 | 0 | 1 | 1 | 1 | 1 | 1 | 1 | 11 |
| 1451 | 1019 | 1194 | 855 | 176 | 53.2369146 | 1 | 1 | 1 | 1 | 1 | 1 | 0 | 1 | 1 | 1 | 1 | 1 | 1 | 12 |
| 1452 | 340 | 503 | 856 | 164 | 70.4659162 | 1 | 1 | 1 | 1 | 1 | 1 | 1 | 1 | 1 | 1 | 1 | 1 | 1 | 13 |
| 1455 | 3444 | 3642 | 857 | 199 | 70.30607583 | 1 | 1 | 1 | 1 | 1 | 1 | 0 | 1 | 1 | 1 | 1 | 1 | 1 | 12 |
| 1459 | 438 | 592 | 858 | 155 | 67.44868035 | 1 | 0 | 1 | 1 | 1 | 1 | 0 | 1 | 1 | 1 | 1 | 1 | 1 | 11 |
| 1460 | 1801 | 1965 | 859 | 165 | 59.20661157 | 1 | 1 | 1 | 1 | 1 | 1 | 0 | 1 | 1 | 1 | 1 | 0 | 1 | 11 |
| 1462 | 1645 | 1802 | 860 | 158 | 49.48216341 | 1 | 1 | 1 | 1 | 1 | 1 | 1 | 1 | 1 | 1 | 1 | 0 | 1 | 12 |
| 1464 | 1792 | 1947 | 861 | 156 | 64.45221445 | 1 | 0 | 1 | 1 | 1 | 1 | 0 | 1 | 1 | 1 | 1 | 1 | 1 | 11 |
| 1466 | 810 | 1000 | 862 | 191 | 59.05759162 | 1 | 0 | 1 | 1 | 1 | 0 | 1 | 1 | 1 | 1 | 1 | 1 | 1 | 11 |
| 1469 | 2061 | 2252 | 863 | 192 | 55.46875 | 1 | 0 | 1 | 1 | 1 | 1 | 1 | 1 | 1 | 1 | 1 | 1 | 1 | 12 |
| 1470 | 4419 | 4623 | 864 | 205 | 74.33395872 | 1 | 1 | 1 | 1 | 1 | 1 | 1 | 1 | 1 | 1 | 1 | 1 | 1 | 13 |
| 1470 | 4626 | 4789 | 865 | 164 | 67.26860538 | 1 | 1 | 1 | 1 | 1 | 1 | 1 | 1 | 1 | 1 | 1 | 1 | 1 | 13 |
| 1472 | 111 | 262 | 866 | 152 | 63.81578947 | 1 | 0 | 1 | 1 | 1 | 1 | 1 | 1 | 1 | 1 | 1 | 1 | 1 | 12 |
| 1475 | 408 | 579 | 867 | 172 | 55.95607235 | 1 | 1 | 0 | 1 | 1 | 0 | 1 | 1 | 1 | 1 | 1 | 0 | 1 | 10 |
| 1477 | 4809 | 4994 | 868 | 186 | 63.97034865 | 1 | 0 | 1 | 1 | 1 | 1 | 1 | 1 | 1 | 1 | 1 | 1 | 1 | 12 |
| 1478 | 2846 | 3047 | 869 | 202 | 60.1260126 | 1 | 1 | 1 | 1 | 1 | 0 | 1 | 1 | 1 | 1 | 1 | 0 | 1 | 11 |
| 1478 | 3681 | 3876 | 870 | 196 | 59.95361781 | 1 | 1 | 1 | 1 | 1 | 0 | 1 | 1 | 1 | 1 | 1 | 0 | 1 | 11 |
| 1478 | 4018 | 4198 | 871 | 181 | 69.39226519 | 1 | 1 | 1 | 1 | 1 | 0 | 1 | 1 | 1 | 1 | 1 | 0 | 1 | 11 |
| 1478 | 4200 | 4532 | 872 | 333 | 69.03630904 | 1 | 1 | 1 | 1 | 1 | 0 | 1 | 1 | 1 | 1 | 1 | 0 | 1 | 11 |
| 1479 | 1560 | 1740 | 873 | 181 | 72.45360533 | 1 | 1 | 1 | 1 | 1 | 1 | 1 | 1 | 1 | 1 | 1 | 1 | 1 | 13 |
| 1479 | 1906 | 2119 | 874 | 214 | 70.12940331 | 1 | 1 | 1 | 1 | 1 | 1 | 1 | 1 | 1 | 1 | 1 | 1 | 1 | 13 |
| 1483 | 2350 | 2534 | 875 | 185 | 62.38820639 | 1 | 0 | 1 | 1 | 1 | 1 | 1 | 1 | 1 | 1 | 1 | 0 | 1 | 11 |
| 1483 | 2997 | 3187 | 876 | 191 | 71.71822941 | 1 | 0 | 1 | 1 | 1 | 1 | 1 | 1 | 1 | 1 | 1 | 0 | 1 | 11 |
| 1485 | 1714 | 1934 | 877 | 221 | 53.69806664 | 1 | 1 | 1 | 1 | 1 | 1 | 0 | 1 | 1 | 1 | 1 | 0 | 1 | 11 |
| 1488 | 0 | 224 | 878 | 225 | 69.38271605 | 1 | 0 | 1 | 0 | 1 | 0 | 1 | 1 | 1 | 1 | 1 | 0 | 1 | 9 |
| 1488 | 593 | 823 | 879 | 231 | 73.78547379 | 1 | 0 | 1 | 0 | 1 | 0 | 1 | 1 | 1 | 1 | 1 | 0 | 1 | 9 |
| 1488 | 1258 | 1412 | 880 | 155 | 70.53763441 | 1 | 0 | 1 | 0 | 1 | 0 | 1 | 1 | 1 | 1 | 1 | 0 | 1 | 9 |
| 1488 | 1818 | 1973 | 881 | 156 | 66.89814815 | 1 | 0 | 1 | 0 | 1 | 0 | 1 | 1 | 1 | 1 | 1 | 0 | 1 | 9 |
| 1488 | 3114 | 3325 | 882 | 212 | 66.61425577 | 1 | 0 | 1 | 0 | 1 | 0 | 1 | 1 | 1 | 1 | 1 | 0 | 1 | 9 |
| 1489 | 3619 | 3809 | 883 | 191 | 62.32270347 | 1 | 0 | 1 | 1 | 1 | 1 | 1 | 1 | 1 | 1 | 1 | 0 | 1 | 11 |
| 1489 | 4017 | 4180 | 884 | 164 | 48.99113082 | 1 | 0 | 1 | 1 | 1 | 1 | 1 | 1 | 1 | 1 | 1 | 0 | 1 | 11 |
| 1489 | 4797 | 4959 | 885 | 163 | 49.93865031 | 1 | 0 | 1 | 1 | 1 | 1 | 1 | 1 | 1 | 1 | 1 | 0 | 1 | 11 |
| 1490 | 607 | 835 | 886 | 229 | 74.76776499 | 1 | 0 | 1 | 1 | 1 | 1 | 1 | 1 | 1 | 1 | 1 | 0 | 1 | 11 |
| 1490 | 1190 | 1428 | 887 | 239 | 72.40015215 | 1 | 0 | 1 | 1 | 1 | 1 | 1 | 1 | 1 | 1 | 1 | 0 | 1 | 11 |
| 1490 | 1669 | 1869 | 888 | 201 | 72.95341474 | 1 | 0 | 1 | 1 | 1 | 1 | 1 | 1 | 1 | 1 | 1 | 0 | 1 | 11 |
| 1490 | 1873 | 2028 | 889 | 156 | 71.63170163 | 1 | 0 | 1 | 1 | 1 | 1 | 1 | 1 | 1 | 1 | 1 | 0 | 1 | 11 |
| 1490 | 2032 | 2192 | 890 | 161 | 61.95369848 | 1 | 0 | 1 | 1 | 1 | 1 | 1 | 1 | 1 | 1 | 1 | 0 | 1 | 11 |
| 1493 | 5010 | 5165 | 891 | 156 | 68.62470862 | 1 | 0 | 1 | 1 | 1 | 1 | 1 | 1 | 1 | 1 | 1 | 0 | 1 | 11 |
| 1497 | 1416 | 1567 | 892 | 152 | 66.14832536 | 1 | 0 | 1 | 1 | 1 | 1 | 1 | 1 | 1 | 1 | 1 | 0 | 1 | 11 |
| 1498 | 2901 | 3052 | 893 | 152 | 68.03827751 | 1 | 1 | 1 | 0 | 1 | 1 | 1 | 1 | 1 | 1 | 1 | 0 | 1 | 11 |
| 1498 | 3689 | 3863 | 894 | 175 | 51.03376623 | 1 | 1 | 1 | 0 | 1 | 1 | 1 | 1 | 1 | 1 | 1 | 0 | 1 | 11 |
| 1502 | 436 | 608 | 895 | 173 | 68.53389385 | 1 | 1 | 1 | 1 | 1 | 1 | 1 | 0 | 1 | 1 | 1 | 0 | 1 | 11 |
| 1502 | 699 | 856 | 896 | 158 | 64.63751438 | 1 | 1 | 1 | 1 | 1 | 1 | 1 | 0 | 1 | 1 | 1 | 0 | 1 | 11 |
| 1502 | 913 | 1067 | 897 | 155 | 55.84750733 | 1 | 1 | 1 | 1 | 1 | 1 | 1 | 0 | 1 | 1 | 1 | 0 | 1 | 11 |
| 1504 | 639 | 825 | 898 | 187 | 66.51434127 | 1 | 1 | 1 | 1 | 1 | 1 | 1 | 1 | 0 | 1 | 1 | 0 | 1 | 11 |
| 1504 | 902 | 1057 | 899 | 156 | 66.22377622 | 1 | 1 | 1 | 1 | 1 | 1 | 1 | 1 | 0 | 1 | 1 | 0 | 1 | 11 |
| 1507 | 108 | 271 | 900 | 164 | 65.10532151 | 1 | 1 | 1 | 1 | 1 | 1 | 1 | 0 | 1 | 1 | 1 | 1 | 1 | 12 |
| 1507 | 273 | 477 | 901 | 205 | 59.82261641 | 1 | 1 | 1 | 1 | 1 | 1 | 1 | 0 | 1 | 1 | 1 | 1 | 1 | 12 |
| 1509 | 0 | 188 | 902 | 189 | 78.02469136 | 1 | 1 | 0 | 0 | 1 | 1 | 1 | 0 | 1 | 1 | 1 | 1 | 1 | 10 |
| 1510 | 456 | 645 | 903 | 190 | 67.51196172 | 1 | 0 | 1 | 0 | 1 | 1 | 1 | 1 | 1 | 1 | 1 | 1 | 1 | 11 |
| 1512 | 390 | 564 | 904 | 175 | 78.24242424 | 1 | 1 | 0 | 1 | 1 | 1 | 1 | 1 | 1 | 1 | 1 | 1 | 1 | 12 |
| 1513 | 462 | 743 | 905 | 282 | 61.26155169 | 1 | 1 | 1 | 1 | 1 | 1 | 1 | 0 | 1 | 1 | 1 | 1 | 1 | 12 |
| 1513 | 838 | 1079 | 906 | 242 | 63.39844728 | 1 | 1 | 1 | 1 | 1 | 1 | 1 | 0 | 1 | 1 | 1 | 1 | 1 | 12 |
| 1515 | 204 | 408 | 907 | 205 | 75.04804139 | 1 | 1 | 0 | 1 | 1 | 1 | 1 | 1 | 1 | 1 | 1 | 1 | 1 | 12 |
| 1516 | 255 | 455 | 908 | 201 | 78.0943766 | 1 | 1 | 1 | 1 | 1 | 1 | 1 | 1 | 1 | 1 | 1 | 1 | 0 | 12 |
| 1516 | 540 | 692 | 909 | 153 | 74.06417112 | 1 | 1 | 1 | 1 | 1 | 1 | 1 | 1 | 1 | 1 | 1 | 1 | 0 | 12 |
| 1517 | 259 | 431 | 910 | 173 | 71.20336311 | 1 | 1 | 1 | 1 | 1 | 1 | 1 | 0 | 1 | 1 | 1 | 1 | 1 | 12 |
| 1517 | 442 | 647 | 911 | 206 | 75.67666961 | 1 | 1 | 1 | 1 | 1 | 1 | 1 | 0 | 1 | 1 | 1 | 1 | 1 | 12 |
| 1517 | 759 | 908 | 912 | 150 | 71.23232323 | 1 | 1 | 1 | 1 | 1 | 1 | 1 | 0 | 1 | 1 | 1 | 1 | 1 | 12 |
| 1525 | 157 | 312 | 913 | 156 | 59.88733489 | 1 | 1 | 1 | 0 | 1 | 1 | 1 | 1 | 1 | 1 | 1 | 1 | 1 | 12 |
| 1528 | 33 | 251 | 914 | 219 | 74.04870624 | 1 | 1 | 1 | 1 | 1 | 1 | 1 | 0 | 1 | 1 | 1 | 1 | 1 | 12 |
| 1529 | 342 | 524 | 915 | 183 | 64.64646465 | 1 | 1 | 1 | 1 | 1 | 1 | 1 | 0 | 1 | 1 | 1 | 1 | 1 | 12 |
| 1531 | 606 | 804 | 916 | 199 | 73.0318258 | 1 | 1 | 1 | 0 | 1 | 1 | 1 | 1 | 1 | 1 | 1 | 1 | 1 | 12 |
| 1531 | 807 | 977 | 917 | 171 | 66.66666667 | 1 | 1 | 1 | 0 | 1 | 1 | 1 | 1 | 1 | 1 | 1 | 1 | 1 | 12 |
| 1532 | 649 | 798 | 918 | 150 | 66.83838384 | 1 | 1 | 1 | 1 | 1 | 1 | 1 | 1 | 1 | 0 | 1 | 1 | 1 | 12 |
| 1534 | 549 | 700 | 919 | 152 | 65.10167464 | 1 | 1 | 1 | 1 | 1 | 1 | 1 | 0 | 1 | 1 | 1 | 1 | 1 | 12 |
| 1534 | 978 | 1230 | 920 | 253 | 72.19427476 | 1 | 1 | 1 | 1 | 1 | 1 | 1 | 0 | 1 | 1 | 1 | 1 | 1 | 12 |
| 1537 | 1075 | 1274 | 921 | 200 | 69.03636364 | 1 | 1 | 1 | 1 | 1 | 1 | 0 | 1 | 0 | 1 | 1 | 1 | 1 | 11 |
| 1541 | 2856 | 3005 | 922 | 150 | 51.45454545 | 1 | 0 | 1 | 1 | 1 | 1 | 1 | 0 | 1 | 1 | 1 | 1 | 1 | 11 |
| 1545 | 819 | 975 | 923 | 157 | 72.06137811 | 1 | 1 | 1 | 1 | 1 | 1 | 1 | 1 | 0 | 1 | 1 | 1 | 1 | 12 |
| 1548 | 1153 | 1349 | 924 | 197 | 57.81418243 | 1 | 1 | 1 | 1 | 1 | 1 | 1 | 0 | 1 | 1 | 1 | 1 | 1 | 12 |
| 1548 | 1351 | 1520 | 925 | 170 | 67.23707665 | 1 | 1 | 1 | 1 | 1 | 1 | 1 | 0 | 1 | 1 | 1 | 1 | 1 | 12 |
| 1548 | 1573 | 1759 | 926 | 187 | 60.69518717 | 1 | 1 | 1 | 1 | 1 | 1 | 1 | 0 | 1 | 1 | 1 | 1 | 1 | 12 |
| 1549 | 63 | 228 | 927 | 166 | 65.60788609 | 1 | 1 | 1 | 1 | 1 | 1 | 1 | 0 | 1 | 1 | 1 | 1 | 1 | 12 |
| 1549 | 315 | 573 | 928 | 259 | 69.21141921 | 1 | 1 | 1 | 1 | 1 | 1 | 1 | 0 | 1 | 1 | 1 | 1 | 1 | 12 |
| 1549 | 627 | 776 | 929 | 150 | 69.61616162 | 1 | 1 | 1 | 1 | 1 | 1 | 1 | 0 | 1 | 1 | 1 | 1 | 1 | 12 |
| 1550 | 625 | 792 | 930 | 168 | 54.84307359 | 1 | 1 | 1 | 1 | 1 | 1 | 1 | 1 | 1 | 0 | 1 | 1 | 1 | 12 |
| 1552 | 393 | 545 | 931 | 153 | 59.31867697 | 1 | 1 | 1 | 1 | 1 | 1 | 1 | 0 | 1 | 1 | 1 | 1 | 1 | 12 |
| 1552 | 645 | 798 | 932 | 154 | 71.7630854 | 1 | 1 | 1 | 1 | 1 | 1 | 1 | 0 | 1 | 1 | 1 | 1 | 1 | 12 |
| 1555 | 1599 | 1797 | 933 | 199 | 69.91015684 | 1 | 1 | 1 | 1 | 1 | 1 | 1 | 0 | 1 | 1 | 1 | 1 | 1 | 12 |
| 1555 | 2025 | 2187 | 934 | 163 | 69.27867633 | 1 | 1 | 1 | 1 | 1 | 1 | 1 | 0 | 1 | 1 | 1 | 1 | 1 | 12 |
| 1557 | 373 | 524 | 935 | 152 | 62.63955343 | 1 | 1 | 1 | 1 | 1 | 1 | 1 | 1 | 1 | 1 | 1 | 1 | 0 | 12 |
| 1558 | 207 | 493 | 936 | 287 | 55.63826417 | 1 | 1 | 1 | 1 | 1 | 1 | 1 | 0 | 1 | 1 | 1 | 1 | 1 | 12 |
| 1561 | 433 | 637 | 937 | 205 | 71.12195122 | 1 | 0 | 1 | 1 | 1 | 1 | 1 | 1 | 1 | 1 | 1 | 1 | 0 | 11 |
| 1564 | 1090 | 1263 | 938 | 174 | 69.79275514 | 1 | 1 | 1 | 1 | 1 | 0 | 1 | 1 | 1 | 1 | 1 | 1 | 1 | 12 |
| 1570 | 6 | 174 | 939 | 169 | 71.83073337 | 1 | 1 | 1 | 1 | 1 | 1 | 0 | 1 | 1 | 1 | 1 | 1 | 1 | 12 |
| 1574 | 1083 | 1283 | 940 | 201 | 71.0990502 | 1 | 0 | 1 | 1 | 1 | 1 | 0 | 1 | 1 | 1 | 1 | 1 | 1 | 11 |
| 1578 | 99 | 303 | 941 | 205 | 67.76940133 | 1 | 0 | 1 | 1 | 1 | 1 | 0 | 1 | 1 | 1 | 1 | 1 | 1 | 11 |
| 1584 | 2043 | 2206 | 942 | 164 | 75.27716186 | 1 | 0 | 1 | 1 | 1 | 1 | 1 | 1 | 0 | 1 | 1 | 1 | 1 | 11 |
| 1585 | 971 | 1166 | 943 | 196 | 73.55442177 | 1 | 1 | 1 | 1 | 1 | 1 | 1 | 1 | 1 | 1 | 1 | 0 | 1 | 12 |
| 1587 | 135 | 311 | 944 | 177 | 72.03389831 | 1 | 1 | 1 | 1 | 1 | 1 | 1 | 1 | 1 | 1 | 1 | 0 | 1 | 12 |
| 1588 | 1708 | 1921 | 945 | 214 | 60.43330501 | 1 | 0 | 1 | 1 | 1 | 1 | 1 | 1 | 1 | 1 | 1 | 0 | 1 | 11 |
| 1592 | 1397 | 1559 | 946 | 163 | 59.31399888 | 1 | 1 | 1 | 1 | 1 | 1 | 1 | 1 | 1 | 1 | 1 | 0 | 1 | 12 |
| 1599 | 1754 | 1913 | 947 | 160 | 62.64204545 | 1 | 1 | 1 | 1 | 1 | 1 | 1 | 1 | 1 | 1 | 1 | 0 | 1 | 12 |
| 1600 | 693 | 868 | 948 | 176 | 61.13980716 | 1 | 1 | 1 | 1 | 1 | 1 | 1 | 1 | 1 | 1 | 1 | 0 | 1 | 12 |
| 1602 | 765 | 921 | 949 | 157 | 63.74252075 | 1 | 1 | 1 | 1 | 1 | 1 | 1 | 1 | 1 | 1 | 1 | 0 | 1 | 12 |
| 1602 | 1430 | 1595 | 950 | 166 | 71.61372764 | 1 | 1 | 1 | 1 | 1 | 1 | 1 | 1 | 1 | 1 | 1 | 0 | 1 | 12 |
| 1605 | 2352 | 2522 | 951 | 171 | 64.24774056 | 1 | 1 | 1 | 1 | 1 | 1 | 1 | 1 | 1 | 1 | 1 | 0 | 1 | 12 |
| 1606 | 874 | 1040 | 952 | 167 | 67.4251497 | 1 | 0 | 1 | 1 | 1 | 1 | 1 | 1 | 1 | 1 | 1 | 0 | 1 | 11 |
| 1609 | 164 | 344 | 953 | 181 | 62.43093923 | 1 | 0 | 1 | 1 | 1 | 1 | 1 | 1 | 1 | 1 | 1 | 0 | 1 | 11 |
| 1611 | 1083 | 1301 | 954 | 219 | 67.01259167 | 1 | 1 | 1 | 1 | 1 | 1 | 1 | 1 | 1 | 1 | 1 | 0 | 1 | 12 |
| 1612 | 1146 | 1295 | 955 | 150 | 68.63703704 | 1 | 0 | 0 | 1 | 1 | 1 | 1 | 1 | 1 | 1 | 1 | 0 | 1 | 10 |
| 1612 | 1375 | 1524 | 956 | 150 | 59.40740741 | 1 | 0 | 0 | 1 | 1 | 1 | 1 | 1 | 1 | 1 | 1 | 0 | 1 | 10 |
| 1623 | 457 | 618 | 957 | 162 | 58.52974186 | 1 | 1 | 1 | 1 | 1 | 1 | 0 | 0 | 1 | 1 | 1 | 1 | 1 | 11 |
| 1625 | 231 | 442 | 958 | 212 | 65.13722127 | 1 | 1 | 1 | 1 | 1 | 1 | 0 | 0 | 1 | 1 | 1 | 1 | 1 | 11 |
| 1628 | 1101 | 1284 | 959 | 184 | 69.62450593 | 1 | 1 | 1 | 0 | 1 | 1 | 1 | 1 | 1 | 1 | 1 | 0 | 1 | 11 |
| 1629 | 2604 | 2810 | 960 | 207 | 72.07729469 | 1 | 1 | 1 | 1 | 1 | 1 | 1 | 1 | 0 | 1 | 1 | 0 | 1 | 11 |
| 1631 | 1539 | 1868 | 961 | 330 | 71.39393939 | 1 | 1 | 1 | 1 | 1 | 1 | 1 | 1 | 0 | 1 | 1 | 0 | 1 | 11 |
| 1637 | 882 | 1035 | 962 | 154 | 66.7027417 | 1 | 1 | 1 | 0 | 1 | 1 | 0 | 0 | 1 | 1 | 1 | 0 | 1 | 9 |
|  |  |  |  |  | **Number of enriched sites:** | **946** | **597** | **937** | **916** | **962** | **855** | **874** | **874** | **927** | **937** | **959** | **865** | **954** | **11603** |
